# Precision Automation of Cell Type Classification and Sub-Cellular Fluorescence Quantification from Laser Scanning Confocal Images

**DOI:** 10.3389/fpls.2016.00119

**Published:** 2016-02-09

**Authors:** Hardy C. Hall, Azadeh Fakhrzadeh, Cris L. Luengo Hendriks, Urs Fischer

**Affiliations:** ^1^Department of Forest Genetics and Plant Physiology, Umeå Plant Science Centre, Swedish University of Agricultural SciencesUmeå, Sweden; ^2^Centre for Image Analysis, Uppsala UniversityUppsala, Sweden

**Keywords:** automated image analysis, confocal microscopy, Arabidopsis, hypocotyl, automated phenotyping, code:matlab

## Abstract

While novel whole-plant phenotyping technologies have been successfully implemented into functional genomics and breeding programs, the potential of automated phenotyping with cellular resolution is largely unexploited. Laser scanning confocal microscopy has the potential to close this gap by providing spatially highly resolved images containing anatomic as well as chemical information on a subcellular basis. However, in the absence of automated methods, the assessment of the spatial patterns and abundance of fluorescent markers with subcellular resolution is still largely qualitative and time-consuming. Recent advances in image acquisition and analysis, coupled with improvements in microprocessor performance, have brought such automated methods within reach, so that information from thousands of cells per image for hundreds of images may be derived in an experimentally convenient time-frame. Here, we present a MATLAB-based analytical pipeline to (1) segment radial plant organs into individual cells, (2) classify cells into cell type categories based upon Random Forest classification, (3) divide each cell into sub-regions, and (4) quantify fluorescence intensity to a subcellular degree of precision for a separate fluorescence channel. In this research advance, we demonstrate the precision of this analytical process for the relatively complex tissues of Arabidopsis hypocotyls at various stages of development. High speed and robustness make our approach suitable for phenotyping of large collections of stem-like material and other tissue types.

## Introduction

Rapid and cheap sequencing technologies have dramatically changed plant breeding and functional genomics in the last decade. Availability of abundant genotyping data shifts the focus within the frame of genetic screening from more efficient genotyping to automated phenotyping technologies. Progress has been made on whole-plant phenotyping solutions, which for example record plant growth, photosynthesis rates, or stress markers (Furbank and Tester, 2011; Dhondt et al., 2013). Whole-plant phenotyping solutions have become commercially available for indoors and outdoors use and are now an integral part of numerous large breeding programs (Cobb et al., 2013; Rahaman et al., 2015). While such whole-plant phenotyping technologies are useful to facilitate breeding for higher yield many important qualitative and developmental traits cannot be assessed by these macroscopic approaches. Especially, genetic screens for chemical composition, anatomical, and mechanical properties of plant raw materials still rely on laborious low-throughput manual phenotyping. A wide range of molecular markers enable the spatially highly resolved study of such traits. Many of these markers can be fluorescently imaged, either through their own inherent fluorescence, via fluorescent fusion proteins, stains, probes, or through immunofluorescence. With the wide range of fluorescence tools, Laser Scanning Confocal Microscopy (LSCM) has become the method of choice to localize and quantify fluorescent markers. LSCM imaging provides fast, sensitive, inexpensive, spatially highly resolved images where pixel intensity reflects target abundance over a wide dynamic range. Fluorescent imaging of morphogen gradients in the Drosophila embryo and of auxin transport proteins in the Arabidopsis shoot and root tip have, for example, greatly contributed to our understanding of pattern formation and development (Gregor et al., 2007; Kierzkowski et al., 2013). Nevertheless, many of these studies rely on comparison of fluorescence intensity between manually defined regions of interest (ROI; Nilufar and Perkins, 2014), e.g., between different cell types. Obviously, manual segmentation into ROIs is labor-intensive and underlies human subjectivity and inconsistency that may severely limit the interpretability of LSCM data.

Computer-assisted quantification of fluorescent targets on a cellular scale over large spatial ranges requires both accurate automatic segmentation and quantification of fluorescence in each individual segment (Luengo Hendriks et al., 2006). Multiple fluorescence sources, including a counterstain for segmentation, can be imaged simultaneously within a single field of view enabling the correlation of the segmented image with a host of other fluorescence targets. Whereas, for animal tissues the application of automated imaging analysis has become popular in the last decade and several software packages for such an approach are freely available (Wiesmann et al., 2015), the adaptation of automated image analysis is lagging behind in plants. This may be related to the limited optical transparency of most plant tissues and therefore a need of thin sectioning of plant specimens resulting in low throughput. In animal tissues the predominant strategy to segment tissues automatically makes use of fluorescently labeled nuclei and region growing algorithms, such an approach may fall short when a fluorescent target is localized to the plasma membrane or the cell wall. In plants, the few attempts to automatically segment tissue have made use of the cell wall stain propidium iodide and the plasma membrane marker FM4-64 (Federici et al., 2012; Pound et al., 2012; Band et al., 2014; Yoshida et al., 2014). These live stains are suitable for embryonic and meristematic tissue but not for mature plant tissues, as the Arabidopsis hypocotyl and stem, which contain terminally differentiated, dead cells with disrupted plasma membranes and which are in comparison to animal tissues of limited optical penetration depth. As an alternative, Sankar et al. (2014) suggested a protocol based on non-fluorescent differential interference contrast images (DIC). However, applicability of this approach is limited by insufficient accuracy, extensively long computing times and incompatibility with confocal imaging.

In hypocotyl and stem, emerging models for wood formation and stem cell research in plants (Jouannet et al., 2015), derivatives of stem cells differentiate into several different cell types of the xylem (inner tissue) and phloem (outer tissue). New divisions of stem cells push daughter cells either toward the in- or out-side, and, with increasing distance from the stem cells, derivatives gradually differentiate. A morphogen-like gradient of the plant hormone auxin has been suggested to regulate stem cell activity and differentiation from cell expansion to cell wall thickening (Uggla et al., 1996; Bhalerao and Fischer, 2014). Changes in morphology and wall composition are indicative for the degree of differentiation and cell type (Liebsch et al., 2014). Compositional changes in the walls of stems have been successfully monitored with the help of monoclonal antibodies against specific wall epitopes (Hall et al., 2013). However, exploitation of such data is currently hampered by manual segmentation, classification and quantification of fluorescent signals. As a consequence, genetic improvement of woody feedstock, e.g., decreased lignin content in xylem fibers, is limited by the absence of automated high-throughput phenotyping tools.

Here, we provide an image analysis pipeline, which (i) accurately segments hypocotyls and stems into individual cells and subcellular regions, (ii) assigns each segment to a cell type, (iii) quantifies fluorescence intensity of the cell wall counterstain and, from a separate channel, quantifies several aspects of fluorescence intensity from cell wall epitopes for each individual segment and cell type, and also (iv) extracts a wealth of morphometric data. The pipeline is coded in a single software environment (MATLAB) and the data can easily be exported and, for example, be used in modeling or multivariate statistics. Short processing times permit large data sets, as required for mutant screens or association mapping, to be analyzed.

## Materials and methods

### Preparation of plant material

Wild-type (*Col-0*) and *knat1*^*bp*−9^ seeds were planted on soil with 18:6 h (light:dark) at 21 °C. Germination times were recorded and hypocotyls excised from plants at 21 and 31 days after germination (dag). Hypocotyls were identified as the 5 mm region below the cotyledons. The 5 mm hypocotyls were immersed in 150 μL 1X PME (stock 2X PME: 50 mM PIPES, 2 mM MgSO_4_, 2 mM EGTA) fixation buffer, within 0.2 ml dome-cap thermal cycler tubes (Thermo Scientific, www.thermoscientificbio.com). Hypocotyls were then subjected to three consecutive 21°C cycles of 5 min vacuum infiltration at 68 kPa, and washed three times in 1X PME (21°C, 68 kPa) prior to storage at 4°C in 1X PME. Segments were individually encased in 1 cm^3^ blocks of 5% agar at 65°C, and stored at 4°C to set. Transverse sections (40 μm thick) were cut from segments using a VT100S vibrating microtome (Leica), separated from agar encasement using a sable hair (“00”) brush, then blocked for at least 1 h in 5% bovine serum albumin in 1X TBST (10 mM Tris, 0.25 M NaCl, 0.1% Tween). Sections were mixed to randomize developmental difference, and randomly allocated from each biological replicate pool, together with 100 μl fresh blocking solution, to wells of a 96-well plate (Ibidi, www.bdbiosciences.com). Blocking solutions were swapped with 5 μl 1:36 dilutions of the LM10 antibody (Complex Carbohydrate Research Center, University of Georgia, US) using gel- loading tips, then sections were incubated at 4°C for 16 h. Hypocotyls were washed twice in 100 μL 1X TBST, then incubated for 1 h at 21°C in the dark in 10 μl of 2 μg/μl Alexa FluorTM 488 donkey anti-rat IgG (H + L) (Agrisera, Sweden). Sections were again washed twice in 40 μL 1X TBST prior to counter- staining with 0.015% Calcofluor White (Sigma-Aldrich, www.sigmaaldrich.com). Sections were again washed twice in 100 μL 1X TBST to remove excess counter-stain and unbound secondary antibody.

### Confocal imaging

Hypocotyl sections were imaged using a confocal laser scanning microscope Zeiss LSM780 point-scan system at 1024 × 1024 pixels (pixel size, 0.6–0.83 μm) with a 10X objective (a plan-apochromat objective with a numerical aperture of 0.45) within the 96-well plate (Ibidi, Germany) fitted with 180 μm-thick coverslip bottoms. Immunofluorescence of AlexaFluor 568 was excited with a 561 nm laser, and emitted light filtered at 575–600 nm. Calcufluor White was subsequently scanned on an independent channel with a 405 nm laser and emission observed at 420–430 nm. Images were saved as “LSM” files with file names that included plate location, antibody, genotype, tissue type, and biological replicate separated by underscores (ex. C08R6_LM10_Col_21-day-old_Hyp_BR1) to permit automated cataloging in the supplied MATLAB analysis pipeline.

### Image analysis–general

The following methods describe the analytical steps taken, and do not serve as an operation manual for processing images. Instead, refer to Supplemental Presentation 2 (“Precision Cell Classification and Quantification Manual”) and Supplemental Video 1 in Presentation 1 (https://vimeo.com/148871821, password: Matlab4Segment) for details on system configuration, experimental setup, parameter optimization and data processing. The image analysis pipeline was implemented in MATLAB using the MATLAB Image Processing toolbox and the DIPimage toolbox (http://www.diplib.org/).

### Experimental setup for image analysis

A working title for the experiment was entered to automatically generate a time-stamped folder to deposit analysis output (Supplemental Figure 5A). The target image files for the experiment that existed within a user-defined source folder were automatically cataloged within the database “ExperInfo” (Supplemental Figure 5). This database recorded file location and levels for experimental factors (age, tissue type, genotype) for each image file.

### Training set generation

Three to four training set images occupying a separate folder were selected that presented the range of morphological variation expected to be encountered in the experimental (testing) image set. These images were imported into MATLAB, and users prompted to enter parameters for image smoothing and segmentation (Supplemental Table 1). After data smoothing of the CFW channel (Supplemental Figure 5), tissue centers were manually selected via a user interface (Supplemental Figure 5), triggering the segmentation algorithm to generate ROIC (entire cell), ROIL (cell lumen), and ROIW (cell wall) for all objects. Prior to delineate the cell borders by Watershed segmentation, Gaussian filtering with a variance of 1 pixel was applied in order to remove background noise. Oversegmentation was corrected by merging regions where the difference in intensity between their minima and the first pixel on the watershed dam touching the two regions was < 10. Lumen boundaries within each watershed region were precisely identified by applying Otsu's thresholding (Otsu, 1979). In some cases, we cropped the image to restrict the amount of tissue or the range of cell types to be examined. This restricted the ROICs to those that fell within the cropped region, and also restricted computation of ROIL and ROIW to those within the cropped region (Supplemental Figure 1). Measurements for all ROIs were saved for later access by the classification algorithm. The number of cell types and their names were subsequently defined, then selected within each training image (Supplemental Figure 5E). A graphical output of the selections was recorded (example in **Figure 2**) along with a MAT-file containing the locations and dimensions of those ROIs. In an iterative process (Supplemental Figures 5F–G), features (Table 1) and cell classes were selected and Random Forest classification (Breiman, 2001) executed for the chosen training set (cell selections). This model was then used to generate class predictions and confidence interval scores for all ROIs in the training set images. An overlay of the entire classification on the original cell selections was generated for each training image (data not shown), along with classification result at varying confidence interval thresholds (as in **Figures 6D–F**). Each training set iteration was outputted to a distinct time-stamped folder for subsequent evaluation. The “ExperInfo” database was updated to include a record of all iterations, their parameters, and the locations of the model data sufficient for classification of test images. Quantitative data for the CFW channel were saved but not further utilized in the experiment. Immunofluorescence quantitation was not performed in the training set generation.

**Table 1:** **Features available in Random Forest classification analysis pipeline**.

**Feature^a^**	**Description^b^**
m.cx	X coordinate of cell center, origin top-left corner of image (μm)
m.cy	Y coordinate of cell center, origin top-left corner of image (μm)
Xnew	X coordinate of cell center, origin center of image (μm)
Ynew	Y coordinate of cell center, origin center of image (μm)
radialV	Radial coordinate of cell center, origin center of image (μm)
angleV	Angular coordinate of cell center, origin center of image (radian)
m.majoraxes	Length of major axis (first principal component axis) of cell (μm)
m.eccenticity	Square root of [1-(Length of minor axis (second principal component axis)^2/(Length of major axis)^2)]
m.theta	The angle between major axis and horizontal axis (radian)
s.area	Number of cell pixels (μm^2)
perimeter	Length of cell perimeter (μm)
s.radius.mean	Average value of radius of cell (distance from object border to the center of object) (μm)
s.radius.min	Minimum value radius of cell (μm)
s.radius.max	Maximum value radius of cell (μm)
extv	Multiplication of Length of major and minor axes (μm^2)
inclV	Acute angle between radial vector of cell (originating form center of image to center of cell) and major axis of cell (radian)
P2A	Circularity of the cell (ratio of Perimeter to Area)
MedianROIC	Median of ROIC intensity
MeanROIC	Average of ROIC intensity
m.theta.real	Angle between radial vector of cell (originating form center of image to center of cell) and first (major) principal component of cell (radian)
MedianROIW	Median of ROIC intensity
MeanROIW	Average of ROIC intensity

a Feature name as it appears in the diagnostic plotting.

b Brief description of the measurement, including appropriate units.

### Quantification of test images

**Figure 1:**
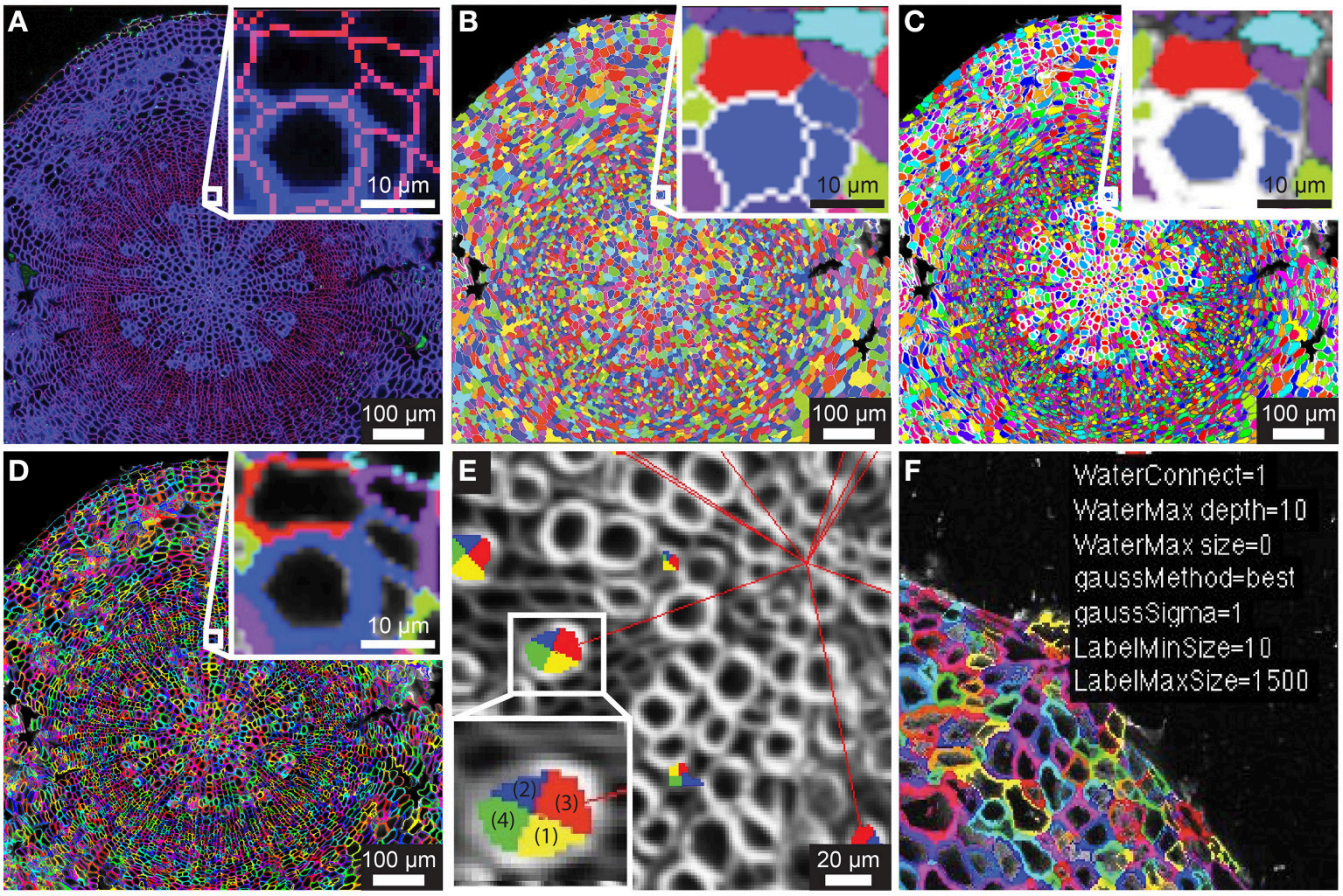
**Segmentation of counterstain channel**. **(A)** Two-channel confocal image of transverse section with watershed boundaries overlaid for entire image and xylem to cambium transition (inset) **(B)** Image segmentation into ROIC (entire cell) objects with random color assignment **(C)** Segmentation of the lumen of each cell (ROIL) with zoomed region depicted in (**A**; inset). **(D)** Cell wall regions (ROIW) derived from difference of ROIC and ROIL with zoomed region depicted in **(A)**. **(E)** Example of quadrant definition for ROIC, with anticlinal quadrants “1” and “2” (yellow and blue) and periclinal quadrants “3” and “4” (green and red) for ROIL being defined by radial axis (red lines to tissue origin). **(F)** Sample of overlay of smoothing and segmentation parameters for diagnostic purposes.

Test images that were suitable for classification by a common training set iteration were processed in a similar manner as the training set (smoothing, defining tissue centers, segmenting) based upon parameters defined for the chosen training set (Supplemental Figures 5H–I). In an iterative approach, classification of the testing set was explored with different training set iterations (as in **Figure 3**). Quantification data and diagnostic plotting of the CFW channel similar to the training set iterations were stored in time-stamped folders. To quantify the signal intensities attributable to the immunolabeling, immunofluorescence channel images were then segmented using ROIC, ROIL, and ROIW generated from the CFW channel segmentation which acted as a mask. In addition, the ROIs were divided into four quadrants (Figure 1E) for higher resolution-based quantification of signal as detailed in Table 2. These data were exported in to separate time-stamped folders for later access. Importantly, the locations of MAT-files containing quantification data for each image were stored in the “ExperInfo” database for access during data assembly and export.

**Table 2:** **Fluorescence measures available for quantification in analysis pipeline**.

**Measure^a^**	**Association^b^**	**Description^c^**
Mean/radial(1)	Each ROI (C,L, or W)	Mean signal of radial region “1” (see Figure 1E), computed for each of ROI (C,L, or W)
Mean/radial(2)	Each ROI (C,L, or W)	Mean signal of radial region “2” (see Figure 1E), computed for each of ROI (C,L, or W)
Mean/tangentrail(3)	Each ROI (C,L, or W)	Mean signal of tangential region “3” (see Figure 1E), computed for each of ROI (C,L, or W)
Mean/tangentrail(4)	Each ROI (C,L, or W)	Mean signal of tangential region “4” (see Figure 1E), computed for each of ROI (C,L, or W)
Std/radial(1)	Each ROI (C,L, or W)	Standard deviation signal of radial region “1” (see Figure 1E), computed for each of ROI (C,L, or W)
Std/radial(2)	Each ROI (C,L, or W)	Standard deviation signal of radial region “2” (see Figure 1E), computed for each of ROI (C,L, or W)
Std/tangentrail(3)	Each ROI (C,L, or W)	Standard deviation signal of tangential region “3” (see Figure 1E), computed for each of ROI (C,L, or W)
Std/tangentrail(4)	Each ROI (C,L, or W)	Standard deviation signal of tangential region “4” (see Figure 1E), computed for each of ROI (C,L, or W)
Size/radial(1)	Each ROI (C,L, or W)	Area signal of radial region “1” (see Figure 1E), computed for each of ROI (C,L, or W); a morphological measure
Size/radial(2)'	Each ROI (C,L, or W)	Area signal of radial region “2” (see Figure 1E), computed for each of ROI (C,L, or W); a morphological measure
Size/tangentrail(3)	Each ROI (C,L, or W)	Area signal of tangential region “3” (see Figure 1E), computed for each of ROI (C,L, or W); a morphological measure
Size/tangentrail(4)	Each ROI (C,L, or W)	Area signal of tangential region “4” (see Figure 1E), computed for each of ROI (C,L, or W); a morphological measure
LumenRPA	Derived, ROIL	Ratio of periclinal to anticlinal ROIL
Lumensignal	Derived, ROIL	Total signal per ROIL
PvD	Derived, ROIL	Punctateness vs. diffuseness of ROIL
WallRPA	Derived, ROIW	Ratio of periclinal to anticlinal ROIW
Wallsignal	Derived, ROIW	Total signal per ROIW
CellRPA	Derived, ROIC	Ratio of periclinal to anticlinal ROIC
Cellsignal	Derived, RO1C	Total signal per ROIC
LumenRPAmean	Derived, ROIL	Ratio of periclinal to anticlinal ROIL
Lumensignalmean	Derived, ROIL	Total signal per ROIL
WallRPAmean'	Derived, ROIW	Ratio of periclinal to anticlinal ROIW
Wallsignalmean	Derived, ROIW	Total signal per ROIW
CellRPAmean	Derived, ROIW	Ratio of periclinal to anticlinal ROIC

a *Name of fluorescence measure as it appears in diagnostic plotting*.

b *Regions of interest (ROIs) to which the measure applies. Those marked as “derived” are computed from other measures generated from the ROIs*.

c *Description of how each measure is computed*.

### Data review and assembly

The final step in the pipeline is to assemble the data for export based upon information stored in “ExperInfo” regarding which images were processed, and where the associated data is stored. From a user prompt, we selected the levels of each factor (ex. specific antibodies from the “antibody” factor), and files with those properties were concatenated into a common file (“DataRawCompile”) to be used in downstream (multivariate) analysis in MATLAB or another environment. We assembled a pipeline that facilitates iterative summary plotting of spatial maps of features for any image present in the assembled data set. “DataRawCompile” was then used to generate means and standard deviations for each cell class within each image (as in **Figures 5**, **6**). The pipeline also permits iterative comparative plotting of the summary statistics (bar plots) for any combination of images present in the output data set. The structure of the output file “DataRawCompile” is detailed in Supplemental File 2.

## Results

### Automated image segmentation of confocal counterstain channel

Embryonic and meristematic plant tissues have been successfully segmented with the help of propidium iodide. However, since propidium iodide is not retained in the cell wall of dead cells, this fluorescent stain is not suitable as a counterstain for segmentation of mature or fixed plant tissues. As an alternative to propidium iodide, we tested calcofluor white (CFW), which binds to cellulose and chitin in cell walls of plants, fungi and bacteria. In order to visualize boundaries between cells we counterstained cell walls of 21-day-old Arabidopsis hypocotyls with CFW and acquired images with a CLSM in a separate reference channel (Channel 1). CFW fluorescence was restricted to cell walls and not detected in cell lumen. After smoothing the reference channel, the watershed segmentation algorithm identified the outer boundaries of cells. Watershed dams matched cell-cell boundaries closely with little over-segmentation, and the ROIs for entire cells, denoted ROIC, matched morphology closely (Figures 1A,B). The lumen boundary within each watershed region was precisely identified using Otsu's threshold algorithm to define ROIL (Figure 1C). Cell wall regions, referred to as ROIW, could be derived as the set difference of ROIC and ROIL (Figures 1D,F), thus giving us two distinct regions of the cell (wall/ROIW and lumen/ROIL). With the help of a manually selected center of tissue, ROIC, L, and W were sub-divided into interior/exterior and left/right (lateral) quadrants in order to study polar or axial distribution of fluorescent signals (Figure 1E).

### Manual selection of training set cells for classification model

**Figure 2:**
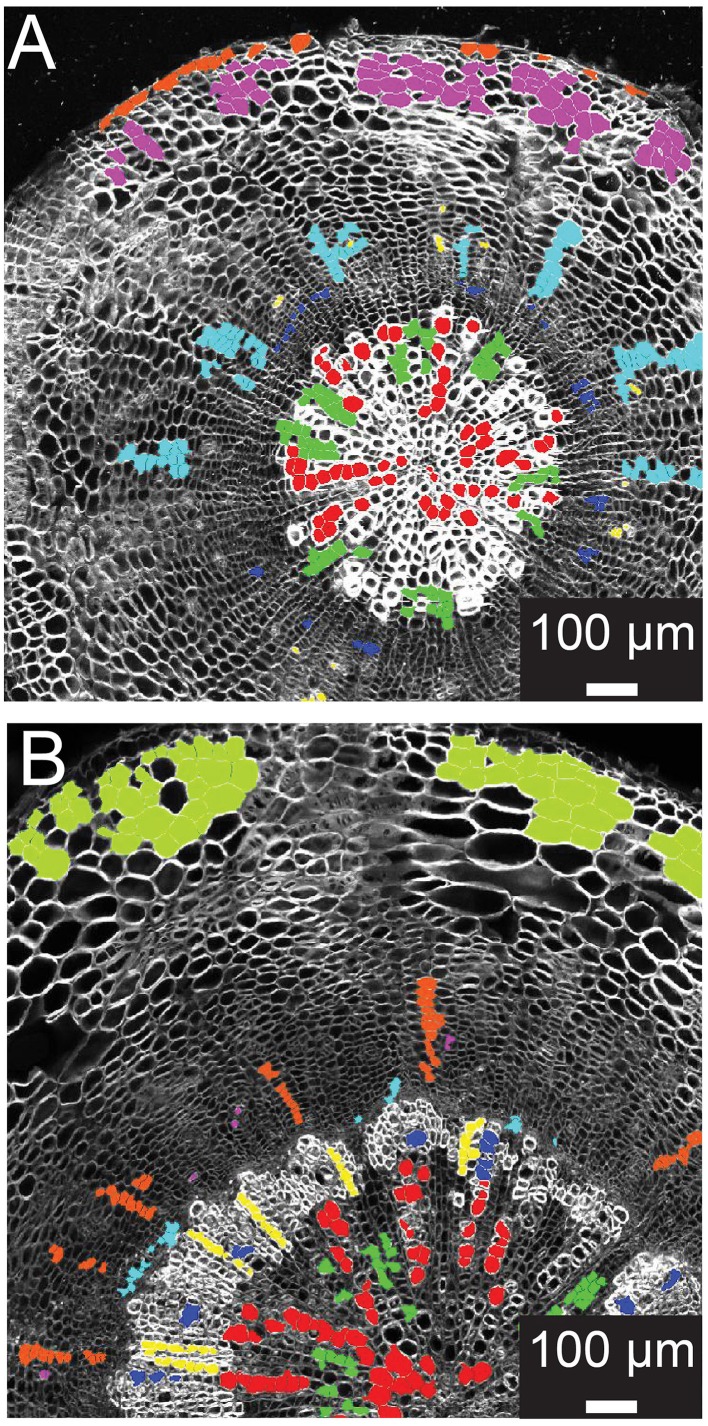
**Training set selection from ROIC segmentation result**. **(A)** Representative 21-day-old wild-type hypocotyl tissues showing selections for xylem I vessels (red), xylem I parenchyma (green), cambium (navy blue), phloem fibers (yellow), phloem parenchyma (light blue), cork (purple), and epidermis (orange). **(B)** Representative 31-day-old wild-type hypocotyl tissues showing xylem I vessels (red), xylem I parenchyma (green), xylem II vessels (navy blue), xylem II fibers (yellow), cambium (light blue), phloem fibers (purple), phloem parenchyma (orange), and cortex (olive).

Supervised learning algorithms have been shown successful for classifying image segments corresponding to individual cells into cell type categories (Field et al., 2010; Sankar et al., 2014). These algorithms require the creation of training sets, i.e., in our case manually assigned classification of cells into groups of user-defined cell types. ROICs of reference images provide sufficient context for manual classification of representative cells for each of the cell types. We defined six different cell types in 21-day-old hypocotyls: xylem vessels and parenchyma, cells of the cambial zone, phloem fibers, phloem, and cortical cells (Figure 2A). For each class, cells were chosen that best represented the class, avoiding selection of cells that lay on vague boundaries between cell types or exhibited morphology that were intermediate between cell classes.

During development, organ types such as the Arabidopsis hypocotyl undergo substantial change in tissue composition (additional cell types) and architecture (e.g., cell morphology and relative position in the tissue context). With Arabidopsis hypocotyls, there is added complexity in 31-day-old hypocotyls with the addition of new cell types in the outer xylem (xylem II), demanding another, optimized training. For 31-day-old hypocotyls, we defined eight different cell types that included xylem I vessels, xylem I parenchyma, xylem II vessels, xylem II fibers, cambium, phloem fibers, phloem parenchyma, and cortex (Figure 2B). These selections were used as the basis for subsequent trials to determine the combination of cell types and features to use for classification.

### Feature computation and pre-selection

Given accurate identification of cell boundaries (ROIC), lumen (ROIL), and cell wall (ROIW), abundant, precise morphometric data can be derived that collectively provide a rich set of features to classify segments into different cell type categories. Using the DIPimage package, we measured 22 different features that could be derived from ROIs, covering aspects of position within the tissue, shape, and fluorescence intensity for each segment (Table 1). Many of these ROI measures correlate well with tissue morphology while some of these features, such as “m.cx” and “m.cy” (xy-coordinates), provide non-sense information with respect to cell identity in a radially symmetrical organ (Supplemental Figures 2A,B). Feature selection is generally considered an important step to improving the accuracy of classification (Janecek et al., 2008). With the exception of excluding “m.cx” and “m.cy” measures, we lacked *a priori* evidence to eliminate other variables without first examining their importance to successful classification. It was therefore necessary to take an iterative approach of feature selection, based upon the output of the classification, to arrive at an optimal set of features.

### Classification

We then chose to compare two different supervised learning algorithms: Support Vector Machine (SVM), originally designed for binary classification problems, and Random Forest, developed specifically for multiclass problems. We tested the accuracy of the classification outputs employing all the above-mentioned features. Random Forest outperformed SVM using normalized measures, distance-scaled measures, and untransformed measures (Supplemental Figure 3). Interestingly, the Random Forest model with the untransformed data resulted in the best fit. We therefore focused on Random Forest for the optimization of the classification procedure.

**Figure 3:**
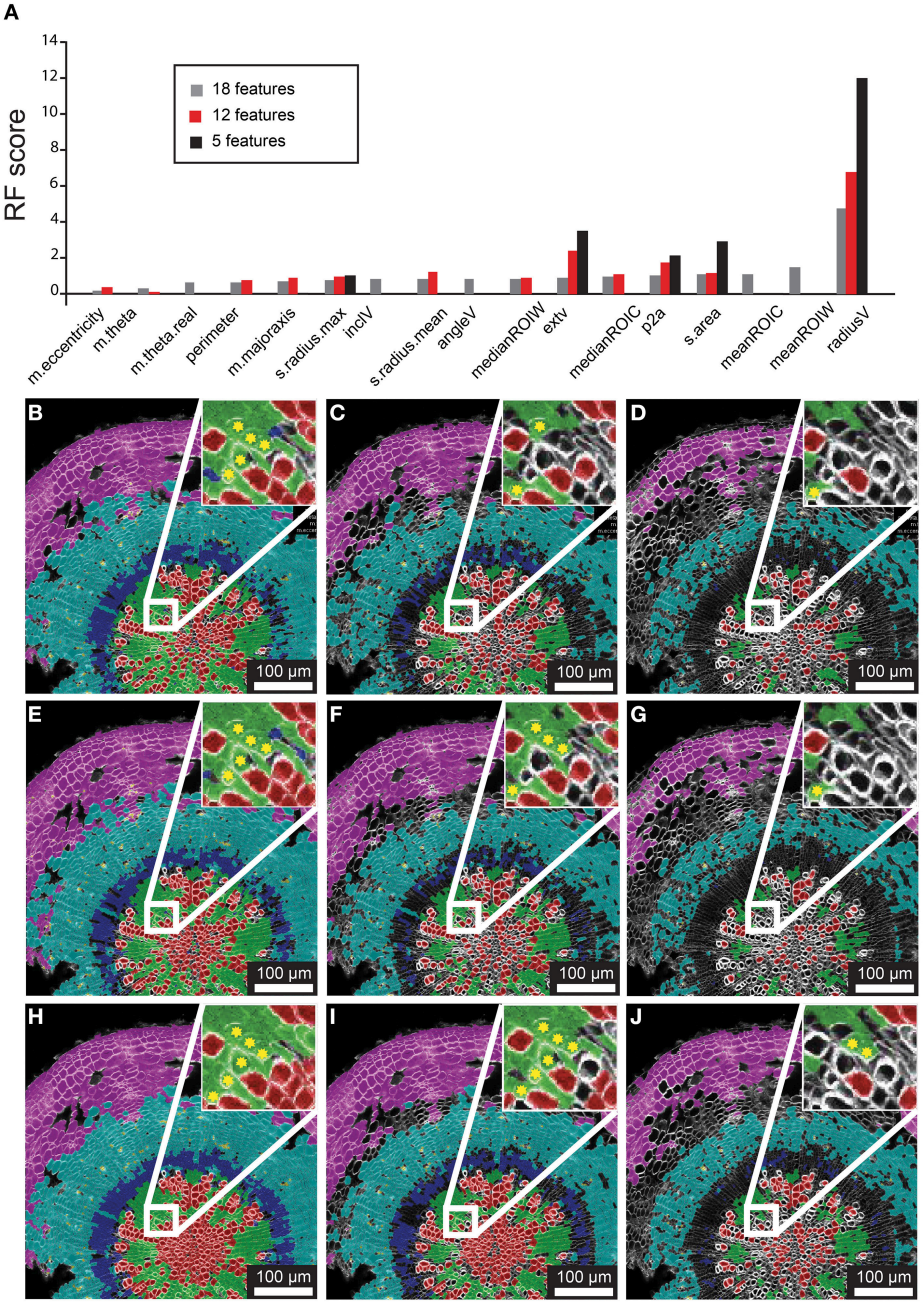
**Feature selection effects on classification for representative 21 dag wild-type hypocotyl**. **(A)** Random Forest scores for features chosen in 18-, 12-, and 5-feature classification iterations. **(B–D)** Classification results for 18-feature classification with 50, 70, and 90% confidence interval filtering, respectively. **(E–G)** Classification results for 12-feature classification with filtering as in **(B–D)**. **(H–J)** Classification result for 5-feature classification with filtering as in **(B–D)**, and **(E–G)** sets. Inset for **(B–J)** depict a sub-region of tissue where misclassifications (asterisks) of xylem-I vessel elements occurs.

As a first step of optimization we assessed the impact of removing features on the classification result, using 21-day-old hypocotyls as a guide. In the first case we admitted the 18 features into the model, all except the Cartesian coordinates (“m.cx,” “m.cy,” “Xnew,” and “Ynew”). The Random Forest model yielded rank scores of the importance of these features (Figure 3A), indicating that the radial displacement from the center of the tissue (“radialV”) was the most discriminate feature underlying the radial organization of the tissue types. Other features that contributed substantially to the discrimination between the different cell types were median fluorescence intensity of ROIC and ROIW (“medianROIC,” “medianROIW”) and the size of the luminal area (“s.area”). The incline angle (“inclV”), which was used as a discriminating feature by Sankar et al. (2014), played a minor role. We used spatial mapping of features (Supplemental Figure 2) to remove six features that we considered redundant with others. Again, “radialV” was dominant, followed by cell wall and cell intensity (“medianROIW,” “medianROIC,” respectively; Figure 3A). Finally, we reduced the selection to five features that were ranked highest in the 12-feature set. Again, “radial” was dominant, while rankings for the remaining features remained similar to those in the 12-feature set (Figure 3A).

The Random Forest algorithm, as with other classification methods, classifies all objects. This invariably results in misclassified objects. However, the Random Forest model assigns a “confidence interval score” to each object such that misclassifications can be largely avoided through filtering. We tested the performance of confidence filtering at 50, 70, and 90% confidence by examining misclassification in cells that were color-coded according to class in an overlay of the original reference channel, considering 18-, 12-, and 5-feature selection sets (Figures 3B–J). It is evident from these panels that increased confidence filtering reduces selection of cells in boundaries of differing cell types such as the cork and phloem parenchyma. The incidence of misclassifications is diminished by confidence filtering where cell types are interspersed, such as with xylem vessels and xylem parenchyma. Conversely, removing low-ranked and seemingly redundant features can lead to increased misclassification (inset of Figures 3B–J).

### Training set versatility

**Figure 4:**
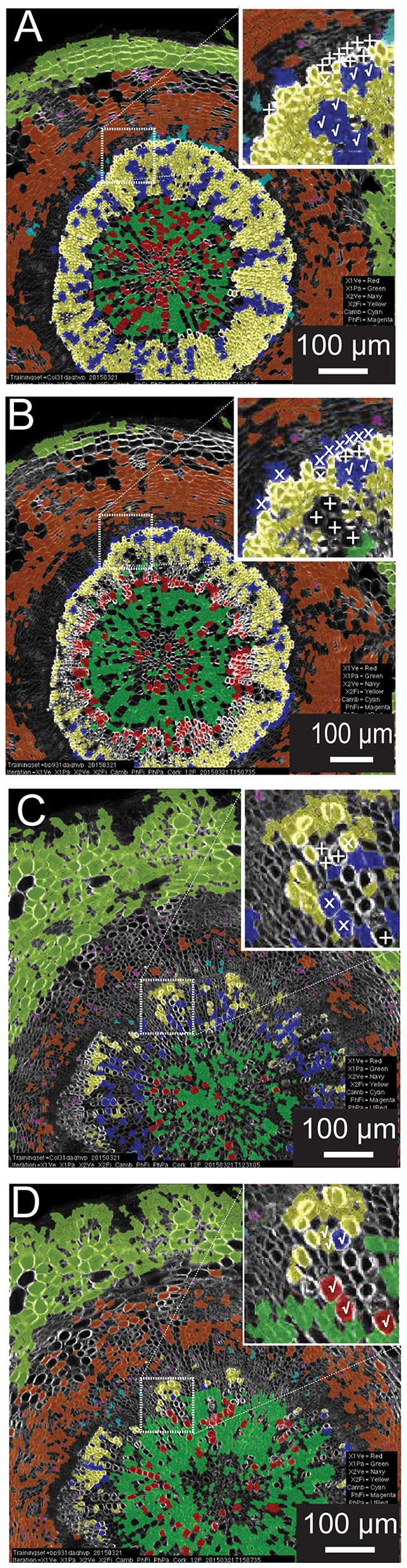
**Reciprocal classification of genotypes from training set iterations of either genotype**. Thirty-one-day-old hypocotyls of wild-type **(A,B)** and *knat1*^*bp*−9^ **(C,D)** genotypes classified with training sets of either *knat1*^*bp*−9^ **(B,D)** and wild-type **(A,C)** 31-day-old hypocotyls. **(A–D)** Classifications passing the 70% confidence interval threshold. Insets in wild type **(A,B)** and *knat1*^*bp*−9^ **(C,D)** are arbitrarily chosen regions at the boundary between Xylem I and II that demonstrate the effect of selection of training set iteration on classification result (classification of selected cells of clear identity are indicated as correct [checkmarks], not classified [“+” sign], and misclassified [“x”]).

A common scenario in developmental biology is the need to survey (to “phenotype”) many genotypes. Automated quantitative morphometrics and fluorescence channel screening offer a means to circumvent the logistic bottleneck in quantifying traits from microscopic tissues. Yet it is not efficient to develop distinct testing sets for each genotype (as with a screen of a mutagenized population). To examine the potential of using a common training set for genotypes with greatly differing tissue organization and morphometric characteristics, we chose to carry out a reciprocal examination between wild type (*Col-0*) and the *knat1*^*bp*−9^ mutant which exhibits irregular radial organization of tissues in the hypocotyl (e.g., reduced xylem fiber formation) and altered luminal areas of xylem vessels (Liebsch et al., 2014). In this examination, we produced 12-feature training set models for each, and then compared result for each genotype (Figure 4). For the wild-type test image (Figures 4A,B), the *knat1*^*bp*−9^ training set classified the vast majority of cortical, phloem parenchyma, phloem fiber, xylem I fiber and vessel cells correctly. However, at the boundary between xylem I and xylem II, relatively more misclassifications or absent classification (low confidence) of vessels and fibers occurred in *t*he wild-type tissue with the *knat1*^*bp*−9^ training set. Similarly, the wild-type training model on *knat1*^*bp*−9^ performed well on the classification of all cell types except for xylem fibers and vessels at the boundary between xylem I and II relative to the *knat1*^*bp*−9^ training set (Figures 4C,D). Generally, with the wild-type training set the boundary between xylem I and II is moved toward the outside of *knat1*^*bp*−9^ hypocotyls (Liebsch et al., 2014) and misclassification at the boundary most likely represent the dominant nature of “radius” in the classification model. Yet, such misclassifications compose a small proportion of the classified cells that present a wealth of information for comparative morphometrics and fluorescence quantification.

**Figure 5:**
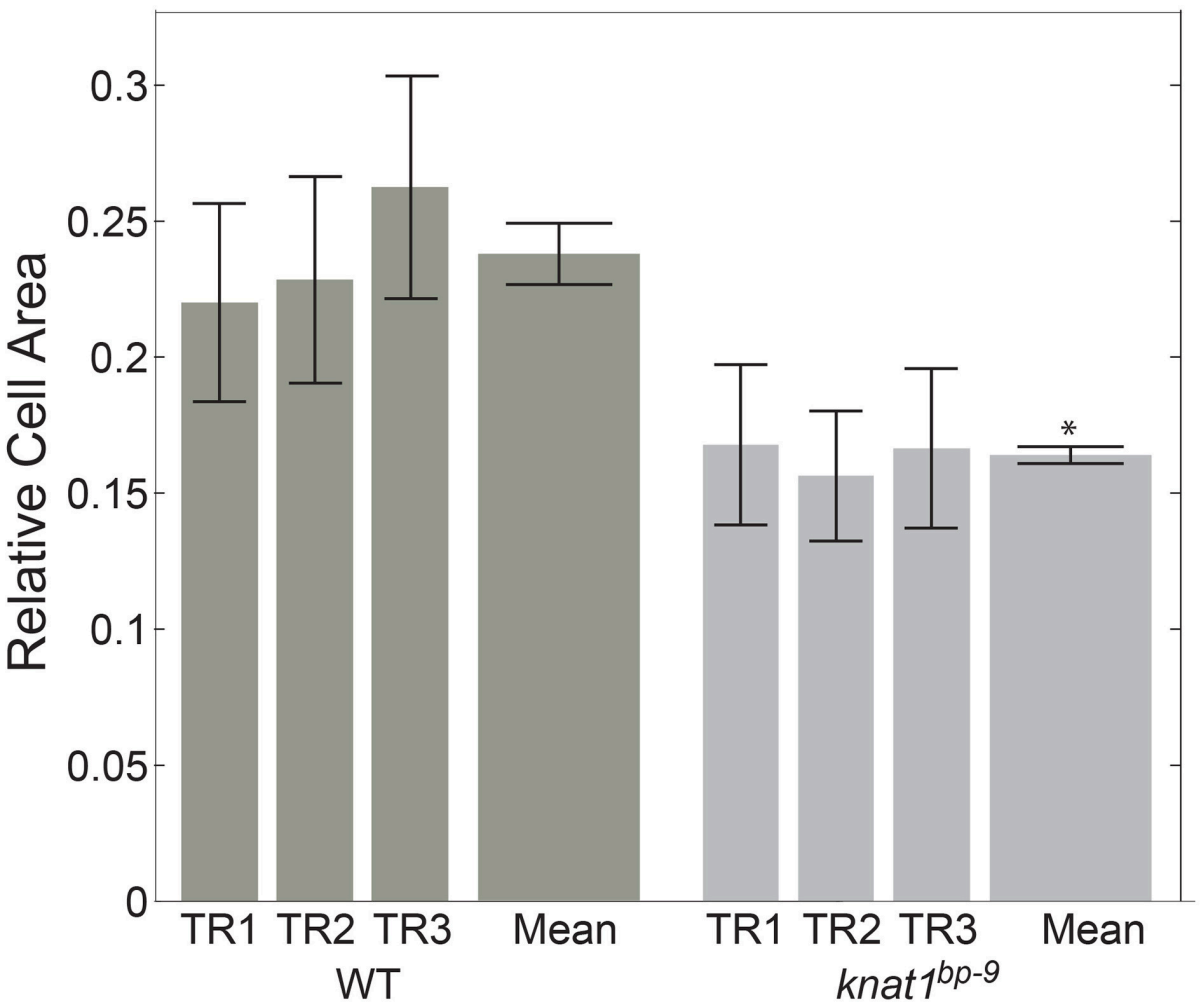
**Quantitative morphological difference between wild-type and *knat1*^*bp*−9^ genotypes**. Relative sizes of secondary cell-walled xylem II vessels (“X-II-Ve”) for technical replicates (separate immunolabeled sections) of representative 31-day-old hypocotyls of wild type and *knat1*^*bp*−9^, with 12-feature classification iteration. Genotype-specific training sets were applied for classifications. Error bars represent standard deviations of relative areas of all cells passing the 70% confidence interval filtering. ^*^t-test, wild type vs. mutant, *p* < 0.05.

In order to see if automated classification could be used as the basis to conduct comparative morphometrics of genotypes, we chose to examine cell area (“s.area”) of xylem vessels of wild type (*Col-0*) and the *knat1*^*bp*−9^ mutant, as *knat1*^*bp*−9^ is known to have smaller cell areas (Liebsch et al., 2014). In this case, three separate sections from the same specimen were quantified, filtered on 70% confidence interval, and the means of all remaining regions of “s.area” measures computed with standard deviations (Figure 5). In line with previously published data (Liebsch et al., 2014), Xylem-II vessels of *knat1*^*bp*−9^ were significantly smaller than in wild type providing evidence that multi-genotype morphometric comparisons are feasible.

### Fluorescence quantification

**Figure 6:**
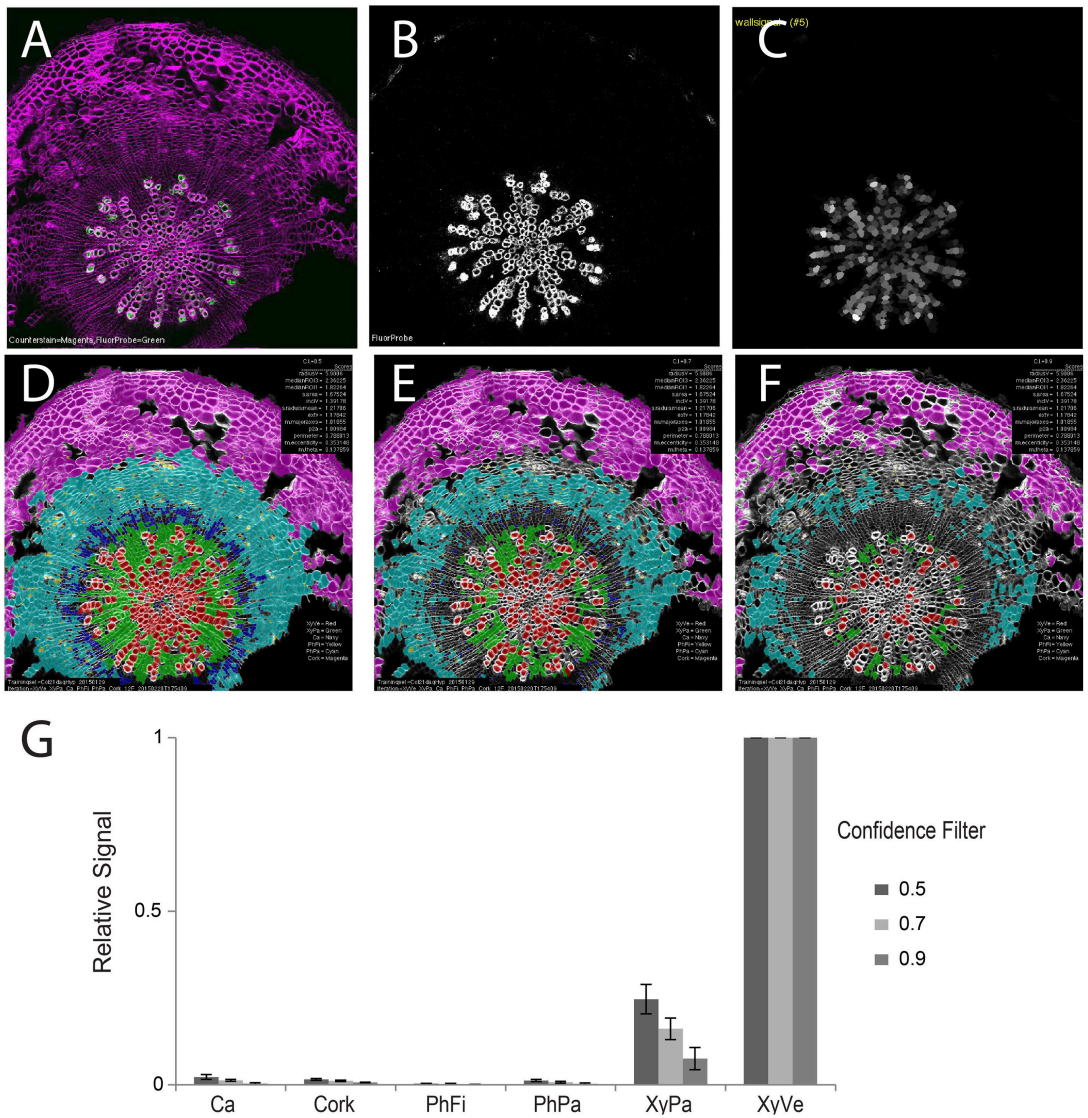
**Quantification of second channel (fluorescence) for a representative tissue (21-day-old wild-type hypocotyl) labeled with xylan-specific antibody (LM10)**. **(A)** Overlay image of CFW counterstain channel (magenta) and LM10 immunolabel (green) channel. **(B)** Isolated immunolabel channel after background fluorescence correction. **(C)** Heatmap of wall signal (grayscale; white, strong signal; black, no signal) for ROIL objects. Classification result and relative wall signal for 50 **(D)**, 70 **(E)**, and 90% **(F)** confidence filtering. **(G)** Quantification of relative fluorescence intensity. Error bars represent the standard deviations of means of relative signal intensities. Three biological replicates.

With robust, accurate classification and subdivision of ROIs to predefined classes, each ROI (and sub-ROI) provides a mask to conduct a variety of measurements of fluorescence (Table 2). While sub-region-specific ROIs provide a high resolution of fluorescence distribution, relative or summative distribution of these intensities can be more informative. As a result, “derived” measures are computed from values in sub-regions (1–4) of various ROIs. For the purpose of demonstration, we probed tissues with an antibody specific to xylan in the secondary cell wall. A fluorescent secondary antibody permitted visualization of epitope localization. Fluorescence was quantified by computing pixel-wise intensities of each ROI, then “derived” measures were subsequently plotted as spatial maps (Supplemental Figure 4). In 21-day-old hypocotyls, secondary cell walls occur exclusively in the xylem vessels, thereby providing a clear case of cell type-specific fluorescence to validate the quantification methodology (Figure 6). Comparison of fluorescence channel (Figure 6B) with a spatial heatmap of fluorescence intensity (“derived_wallsignal”) mapped to ROIC (Figure 6C) demonstrates that the quantification replicates the spatial distribution of fluorescence in the source image. As evidence that the signal is predominantly in the walls, the “derived_lumensignal” values are only moderate in the outermost (youngest) vessels and completely absent in older vessels (Supplemental Figure 4). This is likely due to presence of epitope within the lumen, as it is defined by the segmentation process, of living xylem vessel cells during wall assembly.

As proof-of-concept that the classification provides a meaningful basis to group cells for fluorescence characterization, we next examined the means of fluorescence intensities (“derived_wallsignal”) values for all cells of each cell class, filtering at 50, 70, and 90% (Figures 6D–G, respectively). From this series, it is clear that the xylem vessels are the dominant cell type that exhibits a fluorescence signal. Not evident with visual examination of the spatial map of “derived_wallsignal” (Figure 6C), the xylem parenchyma exhibits a weak signal, too. As increasing stringency reduces the contribution of the xylem parenchyma to the overall signal, we presume that misclassification is the primary cause of signal bleed into that cell type. Yet, stringent filtering (Figure 6G) does not eliminate this signal entirely and thus parenchymal cells of the xylem still retain signal where we expect none (no xylan in this cell type). One explanation for the signal is that there is inaccuracy in establishing the watershed boundaries between cell types that differ greatly in cell wall thickness (Figure 1A, inset).

### High throughput data processing package

**Figure 7:**
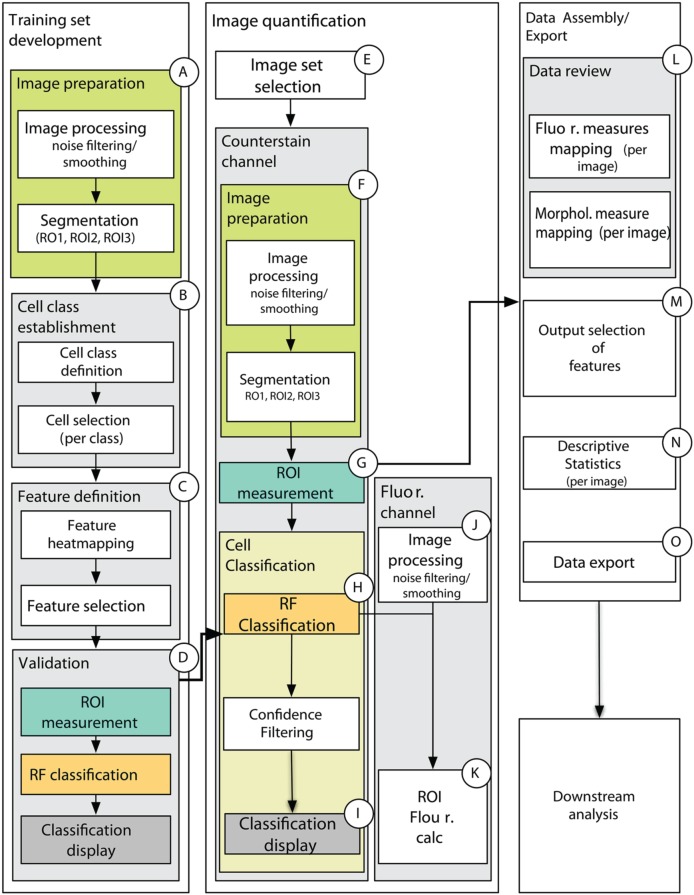
**Schematic of data processing pipeline showing main processing steps for training set development (A–D), image quantification (E–K), and data assembly and export (L–O). (A)** Counterstain channel of two-channel image is first smoothed and segmented on one of the training set images. **(B)** The user then defines the number of cell classes, provides names for cell classes, and select cells for each class in each of the training set images. The cell selections are stored for the experiment. **(C)** The user is presented with spatial maps of the features and chooses which features to use in the classification. This data is stored as an “iteration” for the chosen training set. **(D)** The classification is carried out on the chosen features to produce a model to be used in classifying images of an experimental treatment class in **(E–K)**. **(E)** The user selects a set of images that will use a common training set iteration, then **(F)** images are similarly processed (as in **A**) and **(G)** measurements for the features chosen in the training set iteration are computed for all ROIs obtained by the segmentation in **(F)**. **(H)** Cell classification is carried out using the model generated by the chosen training set iteration and **(I)** spatial maps are generated for 50, 70, and 90% confidence intervals. Classification and confidence scores are stored for each image along with morphological measures used in classification. **(J)** After similar image pre-processing as the counterstain channel, fluorescence measures listed in ROIC.

This methodological proposal presents the reader with a MATLAB-based data analysis pipeline (Supplemental File 1) that provides the complete set of scripts necessary to prepare quantitative data from a complete fluorescence imaging experiment for downstream (multivariate statistical) analysis, while also providing a wealth of images and plots of diagnostic value (Figure 7, detailed in Supplemental Figure 5). Importantly, this set of scripts employs the MATLAB Image Processing toolbox as well as the DIPimage toolbox (http://www.diplib.org/) together providing flexibility and efficiency in a variety of standard image analysis procedures. However, it is the unique assemblage of novel custom scripts using the Random Forest classification model that provide the core analytical steps for generating quantitative data from raw images of plant tissue. Importantly, the core scripts are nested within a graphical user interface that minimizes command line interaction and prompts the user with all of the important parameters for image processing, segmentation, classification and filtering (Supplemental Table 1).

## Discussion

Here, we provide an image analysis toolkit to accurately segment all the cells in transverse sections of hypocotyls, to precisely classify the individual segments into 6 to 8 cell type classes with a specified degree of certainty (confidence interval scores) and to extract precise morphometric data and fluorescence intensity of two different channels for each cell type. By analyzing the distribution of fluorescence emitted by a xylem-vessel-specific marker we obtained accurate segmentation and classification of xylem vessels and parenchymatic cells. This is a relevant improvement over previous attempts of automated cell type classification, which could not distinguish between xylem vessels from parenchyma (Sankar et al., 2014; Montenegro-Johnson et al., 2015). These recent methods relied heavily on spatial localization of cell types within the tissue. However, using coordinates as a sole criterion for the classification can obviously not discriminate between different spatially dispersed cell types, as xylem vessels. Furthermore, the relative location of a cell to the manually selected origin of coordinates can change during development, e.g., the cambium is progressively pushed away from the center of the hypocotyl. Our approach is not only based on the position of each cell within the tissue context but also on features such as cell wall thickness or shape and is therefore expected to be less sensitive to variation in growth than strategies relying on coordinates only.

Automated image analysis is required for large screens involving hundreds of samples, like mutant screens and association mapping. Whereas, segmentation should not be affected by the morphologic variation across the samples, classification might require time-consuming extension of trainings sets. Application of a training set derived from wild type on hypocotyls of *knat1*^*bp*−9^, a mutant which is characterized by severely distorted radial organization, indicated that applying stringent confidence filtering can reduce misclassifications in the mutant efficiently. However, the boundaries between xylem I and II cell types were inaccurately recognized when using a wild-type training set on mutant hypocotyls. This underlies the dominant nature of the radius feature, which measures the radial displacement of a segment from the center of the hypocotyl. Omission of the radius feature, improvements to training sets (image and cell choices) or simply scaling of “radius” are promising means to improve the classification of cells at the boundary between xylem I and II.

Although plant cell walls are considered to be dynamic structures, deposition of wall components is usually stable and irreversible; as opposed to changes in the abundance of short-lived gene products, which can occur in the range of minutes. This, together with easy fixation and efficient preservation of walls as compared to the cytoplasm, makes chemical cell wall properties a good marker for irreversible decisions in cell differentiation. The onset of cell death in xylem vessels is, for example, marked by the lignification of secondary vessel walls (Smith et al., 2013). Our image analysis toolkit permits the analysis of large numbers of commercially available antibodies against different cell wall epitopes. Alternative methods, which offer theoretically the same spatial resolution, to assess the chemical composition of walls of single cells are vibrational spectroscopy and ToF-SIMS (Gorzsás et al., 2011; Gerber et al., 2014; Felten et al., 2015). However, long acquisition times, rather poor image quality and absence of automated image analysis are obstacles that have not yet been overcome by these spectroscopic methods. While in specific cases methods of spatially resolved spectroscopy can provide important chemical information they are, in contrast to the here presented method, not suitable for larger genetic screens or highly resolved time courses.

Here, we present proof-of-concept studies employing images from Arabidopsis hypocotyls. While we expect that automated segmentation of similarly processed plant material, irrespective of the species, should be feasible with no or little adaptation to the script, there may be a need to optimize tissue fixation, sectioning and mounting for other materials than transverse sections of Arabidopsis hypocotyls or stems. On the other hand, application of our pipeline in the analysis of epitope distribution in whole-mount samples, e.g., root tip, or in live imaging of fluorescent markers should be within reach. Images derived from whole-mount samples or live imaging may, however, be more difficult to segment due to lower signal to noise ratios.

In its current form, our data analysis pipeline efficiently and accurately provides a wealth of morphometric data for automatically categorizing cell types of transverse sections of Arabidopsis hypocotyls at various growth stages. Furthermore, our pipeline provides a robust means to accurately quantify immunofluorescence for specific cell types, filterable by confidence scores for individual cells. This manuscript, along with the accompanying script package, thus presents an initial exploration into the application of this MATLAB-based analytical approach of segmentation, classification and quantification of confocal images; one that foreseeably quantifies any number of fluorescence targets on separate channels in more sophisticated fluorescence-based experiments on living or fixed tissues.

## Author contributions

HH and UF designed the research. HH prepared the specimens and captured the images. HH, AF and CL wrote the code and analyzed the data. HH and UF wrote the manuscript.

### Conflict of interest statement

The authors declare that the research was conducted in the absence of any commercial or financial relationships that could be construed as a potential conflict of interest.
