# Supplementary material for: Precision Automation of Cell Type Classification and Sub-Cellular Fluorescence Quantification from Laser Scanning Confocal Images
Source: Front Plant Sci. 2016 Feb 9;7:119. doi: 10.3389/fpls.2016.00119 (PMC4746258; doi:10.3389/fpls.2016.00119)
Supplement: Supplementary file 1 [file Presentation1.PDF]

frontiers

Supplementary Material

Precision automation of cell type classification and sub-cellular fluorescence quantification from laser scanning confocal images

Hardy Hall[1, 3,*], Azadeh Fakhrzadeh[2], Cris L. Luengo Hendriks[2], Urs Fischer[1]

[1] Department of Forest Genetics and Plant Physiology, Umeå Plant Science Centre, Swedish University of Agricultural Sciences, Umeå, Sweden

[2] Centre for Image Analysis, Uppsala University, Uppsala, Sweden

*** Correspondence:** Hardy Hall, Department of Forest Genetics and Plant Physiology, Umeå Plant Science Centre, Swedish University of Agricultural Sciences, SE-901 83 Umeå, Sweden.

hardy.hall@umu.se

[3]Present address: Department of Plant Physiology, Umeå Plant Science Centre, Umeå University, SE-901 87 Umeå, Sweden

Link to demonstration video:

https://youtu.be/BIsxuNKIDBk

1 Supplementary Data

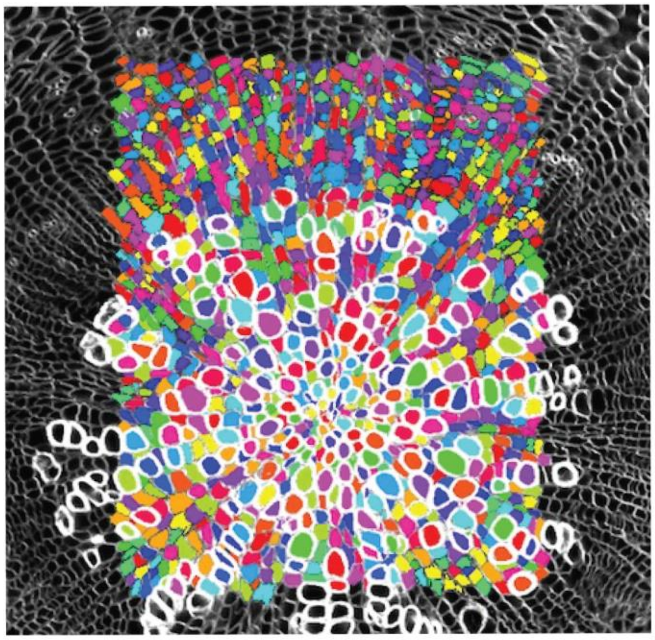

Supplemental Figure 1. Demonstration of cropping feature with ROIL as an example, depicting the spatial limit in the scope of segmentation for ROIL and ROIW (hence classification).

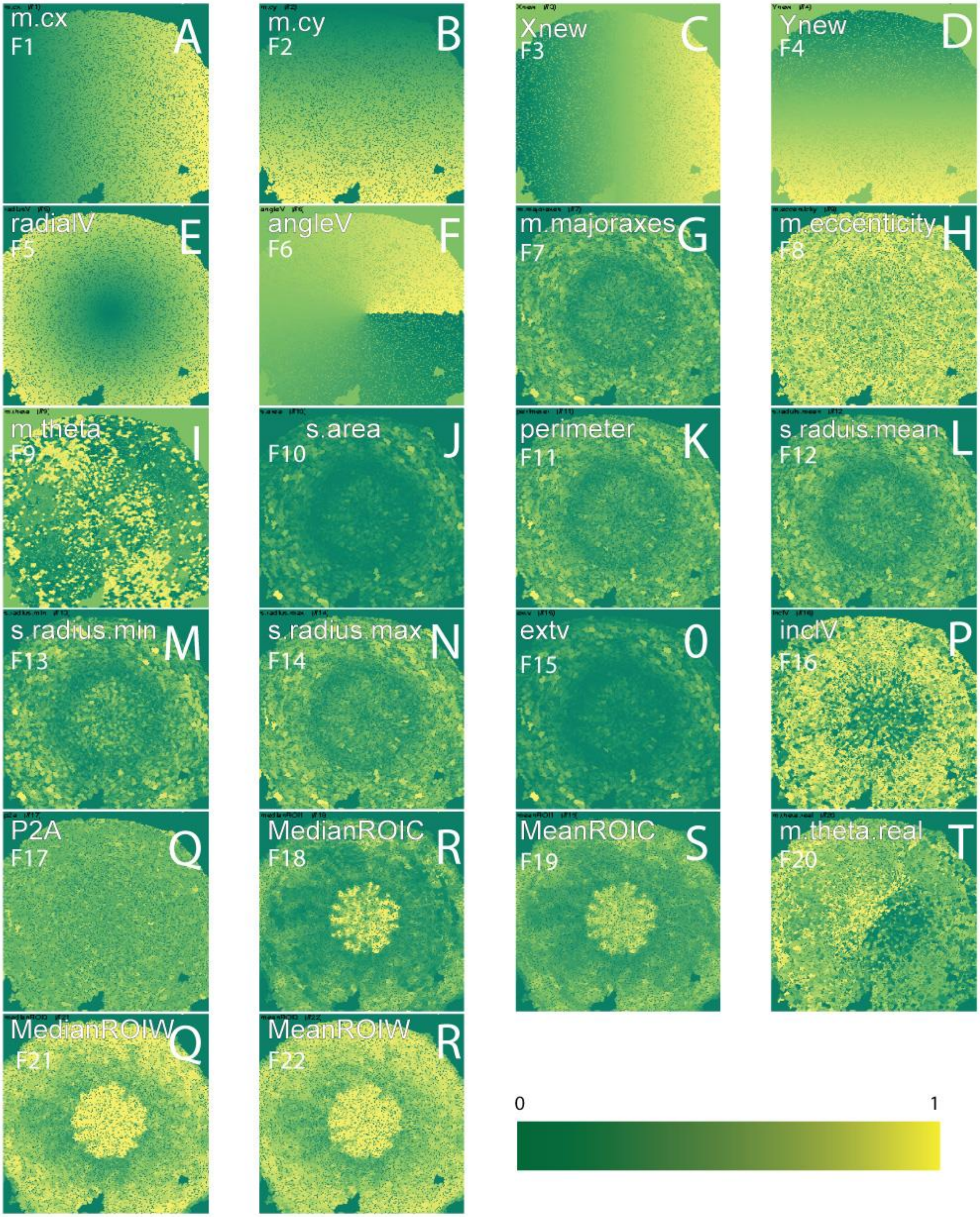

Supplemental Figure 2. Spatial heatmaps of features available for classification. Relative values (scale 0-1) of 22 features available to Random Forest classification model (A-R)_, mapped to ROICs

for a single image. Top left text overlay presents the feature name as it appears in Table 1, as well as the reference number of each features according to the script.

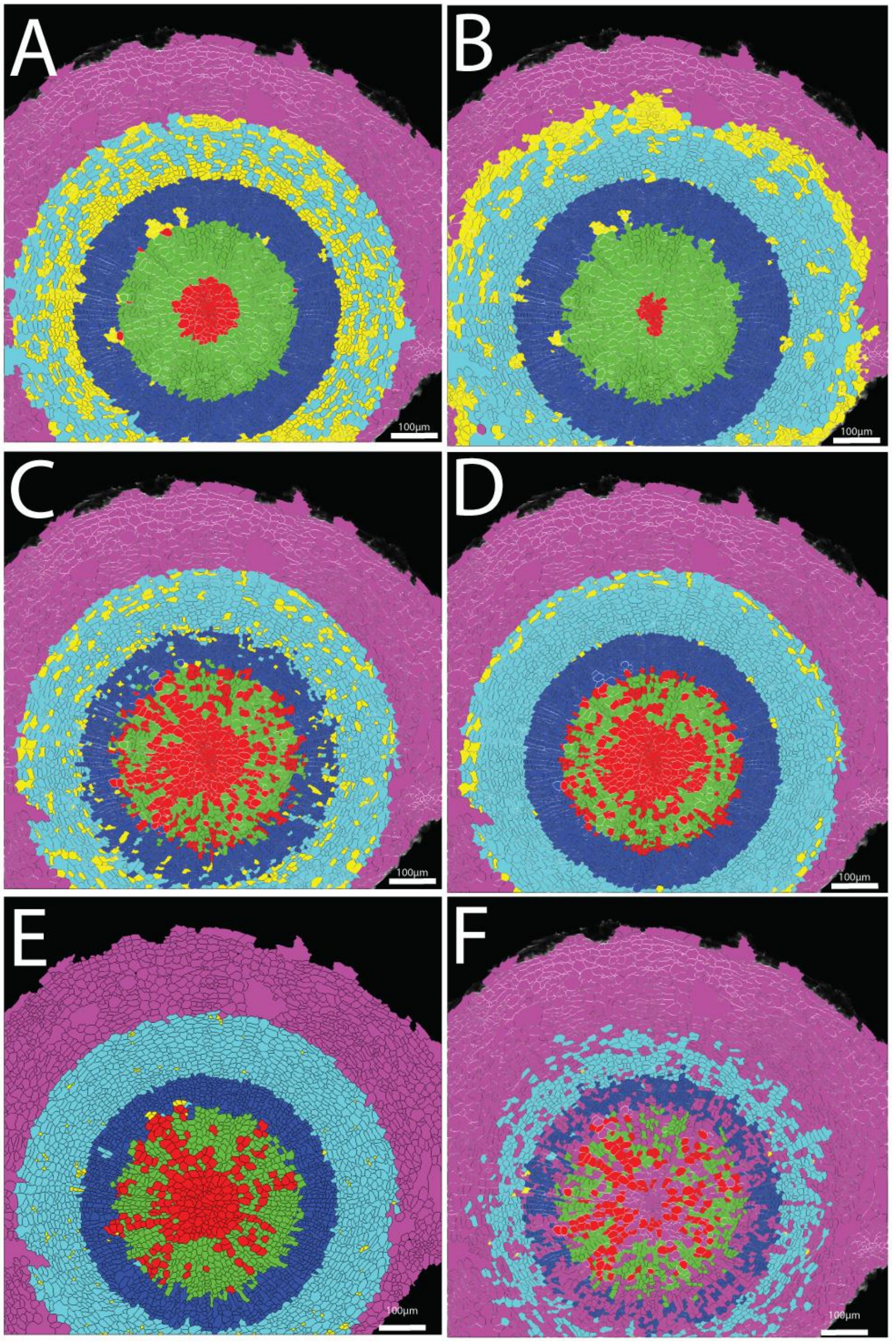

Supplemental Figure 3. Comparison of Random Forest and SVM classification models using three biological replicates for 21-day-old wild-type hypocotyls, where A,C, and E represent the Random Forest product, and B,D, and F display the SVM classification using either A-B) normalized features, C-D) 'SVMOnDistance', and E-F) 'SVMOriginalfeature'.

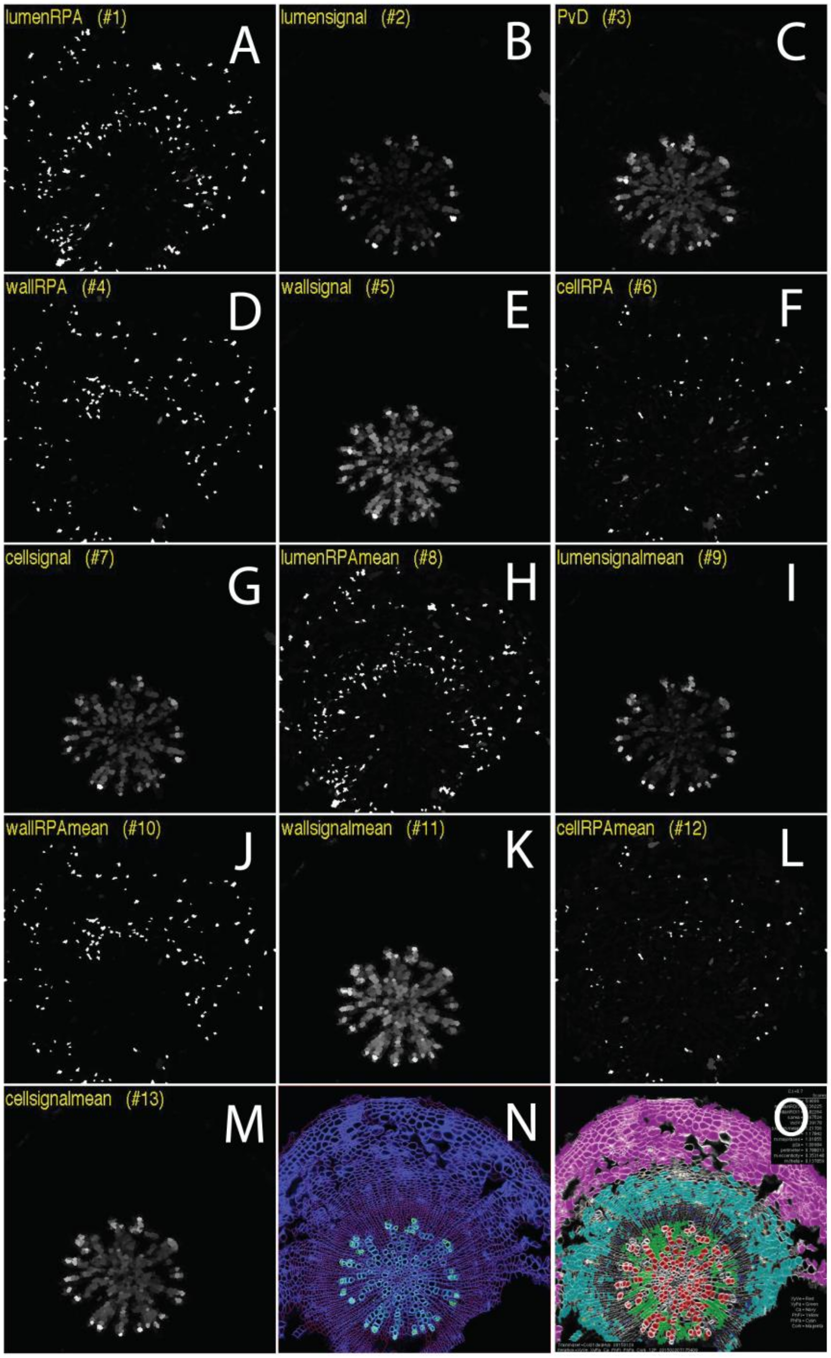

Supplemental Figure 4. Spatial heatmaps of 'derived' fluorescence measures to be exported with classification result and morphometric measures (A-M). N) Segmented reference channel. O) 70% confidence filtered classification result for the corresponding image.

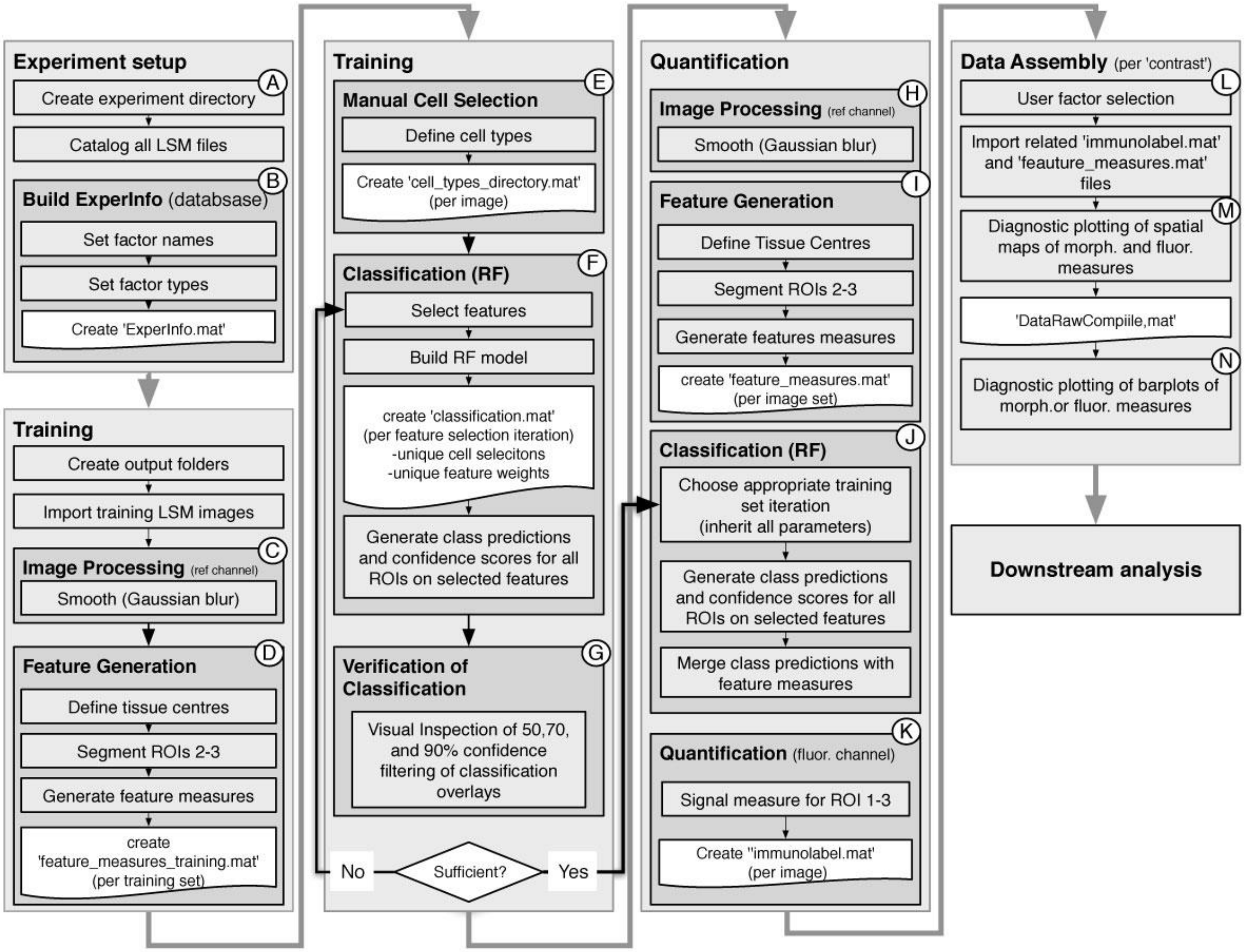

Supplemental Figure 5. Overview of the programmatic steps in the MATLAB-based image quantification pipeline. The process has been organized into four main steps; building the experiment (A-B), developing the training set (C-G), quantifying the target images (H-K), and assimilating the desired quantification data for statistical analysis (L-N). The data is then available for various downstream statistical analysis methods, either in MATLAB, or other environments capable of importing MATLAB data.

Supplemental Table 1. Parameters available to user during training set generation. A) Name of parameter as it appears to user in code and diagnostic plotting. B) The general purpose of the parameter, C) the function to which the parameter applies, D) the package containing the function and E) a general description of the parameter as it is defined in the package.

**Parameter**[A]	**General purpose**[B]	**Function**[C]	**Package**[D]	**Description**[E]
gaussSigma	Denoing/preprocessing	gaussf	DipImage	Gaussian standard deviation
WaterConnect	Segmentation	watershed	DIPimage	A WaterConnect of 1 indicates 4-connected neighbours , a WaterConnect of 2 indicates 8-connected neighbourhood in 2D
WaterMax_depth	Segmentation	watershed	DIPimage	regions up to 'WaterMax_depth ' intensity differences will be merged after segmentation
WaterMax_size	Segmentation	watershed	DIPimage	regions up to 'WaterMax_size 'pixels will be merged after segmentation
LabelConnect	Segmentation	label	DIPimage	A LabelConnect of 1 indicates 4-connected neighbours , a LabelConnect of 2 indicates 8-connected neighbourhood in
LabelMinSize	Segmentation	label	DIPimage	Minimum size of objects to be labeled.
LabelMaxSize	Segmentation	label	DIPimage	Maximum size of objects to be labeled.
NTrees	Classification	TreeBagger	Matlab	Number of decision trees
ConfThreshold (for classification)	Display/Classification	diplayconfidence	Matlab	Coincidence of classification result
Score (for classification)	Output of random forest and is used for displaying classification result	diplayconfidence	Matlab	The probability of every object belonging to every class
