# Supplementary material for: Precision Automation of Cell Type Classification and Sub-Cellular Fluorescence Quantification from Laser Scanning Confocal Images
Source: Front Plant Sci. 2016 Feb 9;7:119. doi: 10.3389/fpls.2016.00119 (PMC4746258; doi:10.3389/fpls.2016.00119)
Supplement: Supplementary file 2 [file Presentation2.PDF]

Precision Cell Classification and Quantification Manual

Table of Contents

General Introduction 2
System Preparation 3
User requirements 3
Software requirements 3
Hardware requirements 3
Data Preparation 3
Experimental Setup 4
Training set overview 7
Training Approach .... **Error! Bookmark not defined.**
Protocol to Generate a Training Set 9
Segmentation, Classification, Fluorescence Quantification Protocol 10

General Introduction

Users can access the full functionality of the pipeline through a single executable file, 'Initiate.m', that provides buttons to initiate the five main activities as outlined in:

1. Setting up the experiment
2. Choosing cells for the training set images
3. Choosing features for classification, and testing the random forest model
4. Processing, segmenting, classifying and quantifying target image sets.
5. Collecting the data for export

These activities define five of the sections in this manual, and are preceded by 'System Preparation' and 'Data Preparation' chapters. 'System Preparation' chapter defines the user background, necessary software, and recommended hardware necessary to execute the scripts. 'Data Preparation' provides the file format requirements, as well as recommendations for the selection of target folders for data output from the pipeline.

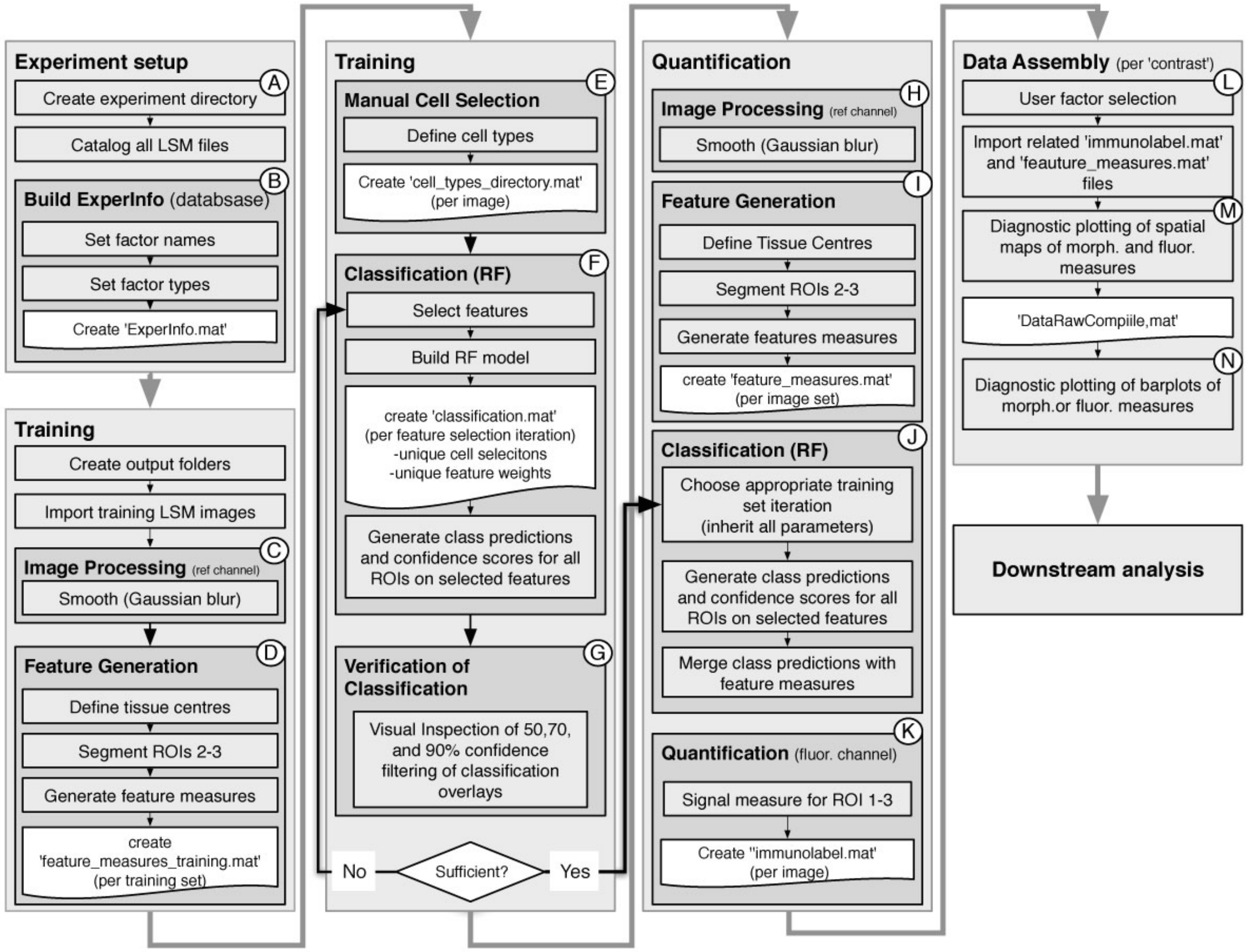

Figure A. (Supplemental Figure 5). Overview of the programmatic steps in the MATLAB-based image quantification pipeline. The process has been organized into four main steps; building the experiment (A-B), developing the training set (C-G), quantifying the target images (H-K), and assimilating the desired quantification data for statistical analysis (L-N). The data is then available for various downstream statistical analysis methods, either in MATLAB, or other environments capable of importing MATLAB data.

System Preparation

User requirements

The pipeline is intended for use by biologists who may not have programming experience but are comfortable handling moderately complex data structures. Yet, this documentation assumes the user has a background in the basic principals of image analysis including image capture, thresholding, and segmentation. For more information, read __cites__. Additionally, users should be aware of the concept of feature-based classification in order to understand the strengths and limitations of the chosen methodology. For more information, read __cites__.

Software requirements

This pipeline was developed in Matlab (Version 2013b), and has been confirmed to run on Mac OS X 10+ and Windows 7+.

We avoided using any Matlab functions that are marked as deprecated (not supported), reducing the likelihood of such forward compatibility issues arising for years to come. This pipeline extensively utilizes the DIPImage toolbox for Matlab (Version 2.6) for specialized image analysis functions that are not found in Matlab's own "image analysis toolkit". The DIPImage libraries must be installed through their own installation package in Windows from the website (link), or manually in Mac OS X. In either case, consult 'dipimage_user_manual.pdf' for instructions. Finally, DIPImage utilizes Java, so the user machine should have the latest available of the Java Runtime environment loaded.

Hardware requirements

Through monitoring CPU and memory loads during processing, we have learned that the time to measure and classify thousands of regions of interest (ROIs) depends largely upon the processing rate where RAM is at least four gigabytes. Matlab can utilize the multiple cores on a typical Mac or PC. 2.4Ghz Intel Core2 Duo with Mac OS X 10.9.5 is up to 1.7 times slower in our tests (6 target images plus 4 training set images) than Intel Core i5 running the same OS (and Matlab version). In either case, memory pressure was low according to the 'Activity Monitor' of OSX. We have not experimented with larger image sets to see if RAM can become a limiting factor on either system.

Data Preparation

This pipeline has been developed to accept confocal images with multiple channels that are of two dimensions (thus excluding z- and time-series). The quality of the segmentation algorithm is a function of the spatial resolution of the image and the contrast of the cell walls (or other cell boundary marker) with cell interiors. The accuracy of the quantitation of ROIs as well as the accuracy of the classification will also be greatly affected by the quality of the images and their segmentation. It is thus critical to optimize and standardize image acquisition across the entire set of experimental images prior to processing images in this pipeline.

This pipeline is designed to carry out batch processes on target image folders. As such, processing and memory limits may be restrictive. Partition your data into sub-folders of less than twenty images prior to starting the experiment. You do not have the option to establish new filepaths for these images once the experimental analysis has been initiated. With a system comparable to our Corei7 and 16GB of RAM, less than twenty images per folder (batch) is strongly recommended.

A graphical user interface (GUI) 'Initiate.m' can be initiated by 'right-click/run', or 'Initiate.fig' by 'double-click'.

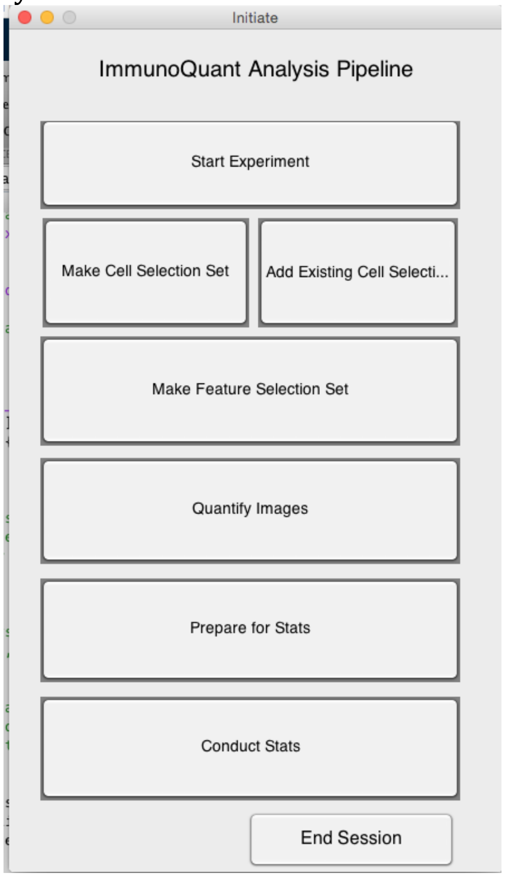

Figure B. 'Initiate' GUI. Note 'add existing cell selecti…' and 'Conduct Stats' have not yet been implemented.

Experimental Setup

The first step in the pipeline is to provide a name for the experiment, and a location for the output directory. A folder is created of the form 'YYYY-MM-DD_[your experiment name]_analysis' with the current date. Repeating the generation of the experiment on the same day will produce a warning message and files within will be overwritten.

You are next prompted to choose a directory where all of the target .LSM files are stored. These files must be of the form:

[element1]_[element2]_[element3]_[elementn].extension, where:

- Element 1-n are either
    - experimental factors (age, tissue type, fluorescent probe, etc…)
    - biological replicates (designated BR1, BR2, etc…)
    - technical replicates (designated TR1, TR2, etc…)
    - other (some archiving convention that is not relevant to the experiment)
- Extension: can be either LSM or TIFF formats that DipImage can read.

It is important that the order of these elements is consistent throughout the set of images since the script establishes the 'levels' (unique names) of each of factor strictly from each filename element. For example, at (2:23;07) of the demo video 'element2' in our experiment was designated 'mAb' (for monoclonal antibody), and contains all of the unique mAb names such as CM07, CM14, etc… The filenames are decomposed into a table of factors ('splitnames') that is used to automatically generate output data based upon user selection of factors and features throughout the pipeline.

This pipeline generates several '.mat' files that are referenced by the pipeline at various images for diagnostic purposes. These are written to two main output locations

1. An output path related to the training images, created in the parent folder to those images, and named according to a) the name you gave the training set and b) the date for generating the training set.
2. An output path related to each target image batch, created in a subfolder of the experiment directory specific to the a) the creation date of the experiment and b) the name of the experiment.

These parent folders cannot be moved during the experimental analysis as several sub-folders contain information required by downstream scripts. In particular, do not move/delete '.mat' files in '01_experiment_matlabfiles' as these are inevitably used by the pipeline.

This stage of the pipeline establishes a database 'ExperInfo' (Figure C) that stores;

1. File paths to all target image files as various cells
    a. filepaths: paths to files
    b. fullpaths: filepaths plus the filenames
2. Factor information
    a. factortype: list of all factor names derived from filename elements
    b. Categorical matrices for each factor containing a list of levels for each factor (eg. mAb, Genotype,…
    c. ProcessedList: a checklist of which of the archived images has been segmented, classified and quantified. Marks them as available for data assimilation, so null values to begin with.
3. Pipeline parameters (made during generation of first training set)

a. Params: stores parameters used in the generation of EACH training set; image processing and segmentation)

The database is created during the ‘Experimental Setup’ stage as ‘YYYY-MM-DD_[your experiment name]_ExperDesign.mat’ within ‘01_experiment_matlabfiles’ of the experiment directory. It is overwritten/updated upon completion of each of the main pipeline stages (as listed in graphical user interface (GUI) triggered by ‘Initiate.m’. The view in Figure C is not accessible through running ‘Initiate.m’ but can be imported into the Matlab workspace for inspection when not executing ‘Initiate.m’ or scripts that it calls.

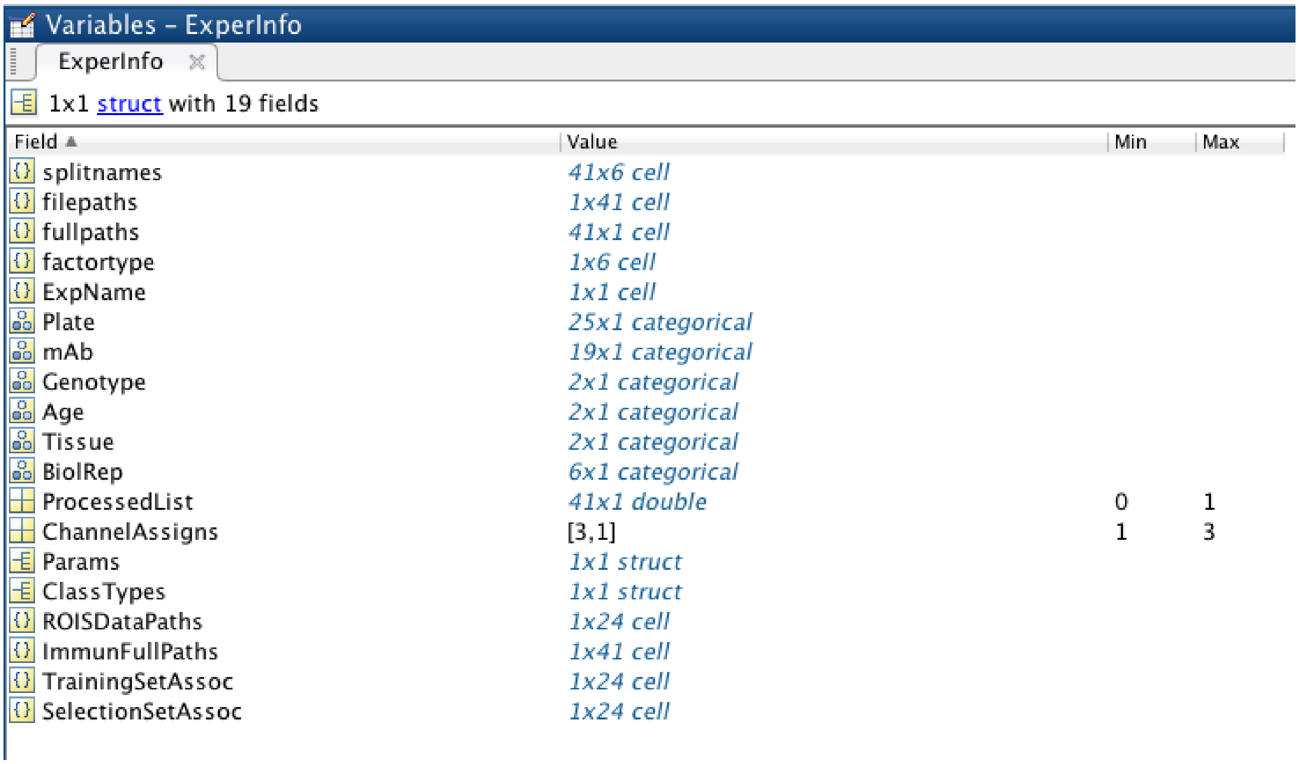

Variables – ExperInfo

ExperInfo

1x1 struct with 19 fields

Field ▲	Value	Min	Max
splitnames	41x6 cell		
filepaths	1x41 cell		
fullpaths	41x1 cell		
factortype	1x6 cell		
ExpName	1x1 cell		
Plate	25x1 categorical		
mAb	19x1 categorical		
Genotype	2x1 categorical		
Age	2x1 categorical		
Tissue	2x1 categorical		
BiolRep	6x1 categorical		
ProcessedList	41x1 double	0	1
ChannelAssigns	[3,1]	1	3
Params	1x1 struct		
ClassTypes	1x1 struct		
ROISDataPaths	1x24 cell		
ImmunFullPaths	1x41 cell		
TrainingSetAssoc	1x24 cell		
SelectionSetAssoc	1x24 cell		

Figure C. ExperInfo directory. Shows the information that is stored in ‘YYYY-MM-DD_[your experiment name]_ExperDesign.mat’ during the course of the experiment.

Training set generation

Following the creation of the experiment, training sets (data matrices as .mat files) are generated from representative training images (.LSM files) of sample sets of a common developmental stage. The 'create_training-set.m' algorithm triggered by 'initate.m' provides the user an interface to select cells that represent cell types that they wish to identify with the classification algorithm. For each image, the user ideally chooses approximately 60 cells per cell type. These selections are indicated in .TIF image, and also in a .MAT file that both match the original image in filename. Measurement of these cells, and classification of the remaining cells in those images, is carried out in the next phase through execution of 'main.m' via 'initate.m'.

Training image selection

Training set type

The number of separate training sets will vary between experiments. Generally, multiple training sets may be required where cell morphologies and positions vary greatly. This is mainly because the classification model establishes which of the feature measures are most relevant for separating cells into classes, and this can vary significantly between treatment classes. In our case, training sets tailored for specific genotypes performed better than those generated for either.

Image Quality

It is important to build a training set that will provide the best possible reference for cell shapes to be recognized in the target images. The most critical factors that will affect the classification model are the accuracy cell property measurements produced during the generation of feature measurements (Figure A.D) as well as the accuracy of the manual classifications during cell selection. As such, images that are chosen for training should ideally be the best available from the experimental images in terms of histology. That is, the cell types should be relatively free from physical distortions produced by sectioning, handling and imaging. Further, confocal imaging quality should have sufficient contrast without over-saturation so that the cell walls are clear relative to lumen. You will need to copy these files into a separate folder.

Number of cell types

The model can tolerate any number of cell types, but we have constrained the number to 13 due to the number of colours available for summary plotting. The model will perform best by selecting fewer, more extreme cell types. Additional cell types are likely to be intermediate forms of more extreme cell types, and likely be misclassified with higher occurrence. You can explore two opposite approaches:

1. User defines the boundaries by selecting cells in a tissue type that span the full range of possible morphologies and positions
2. User selects cells of each cell type that are the most distinct from other cell types, thereby letting the classification algorithm establish the boundaries.

One can observe the output of the classification on the test (and other) images to see if the boundaries themselves provide meaningful information. Filtering on the confidence of the classifications could be used to compare which method is better at providing anatomically

accurate classification, but the boundary still remains an artificial/arbitrary one if the biologist cannot rationalize the position of the boundary.

Protocol to Generate a Training Set
=====================

Introduction

This procedure covers the generation of training sets for classification from a set of reference images for a specific developmental stage and tissue type (stem, hypocotyl). The procedure requires that LSM files that were created with two-channels; one for the counterstain channel upon which segmentation is done, and another channel of immuno data, although only the counterstain channel is relevant. Essentially, they must be collected within the same experiment as the images that users will classify with the 'main.m' algorithm. Consult 'User-documentation/Training_overview.txt' for notes on general application, including some important considerations on how to select cell types.

Materials

-LSM files (>4 recommended) that have approx. 60 cells for each cell type (for classification model robustness)
-Scripts: 'create_training_set.m' algorithm within the provided matlab package in '../scripts' folder.

Procedure
----
1. Generate a folder with the name of the stage and tissue type for which we want to classify cells (ex. '20140929_training_set_21dag_Hyp'). Within it, make a folder called 'LSM_files_and_output'.

2. Populate the 'LSM_files_and_output' folder with files that capture the dynamic range of morphology (tissue arrangement, image quality). (omit: "Place the latest script 'create_training_set.m' in that folder." It is now in main folder)

3
4. Setup the working directory by navigating in the path window of Matlab to the folder where the script package resides.

5. Run the 'create_training_set.m' script by executing the 'run' command from within the text editor, or right-clicking on the 'create_training_set.m' file in the current folder browser of Matlab. Do drag the '.m' file into the command line of Matlab. You may be prompted to 'add to path' or 'add folder' (either is ok).

6. Decide on the number of cells you'll try to identify; you must be able to find (or designate) an instance of each cell type in all images you select. Choose 60 per set... look at the command line for a report of how many cells you've selected. Try to be consistent across cell types and training set images.

7. Decide on the order in which the cell type will be identified in the series of images, and keep a record of this order, perhaps making notes of important qualities of these cells.
-since the output is color-coded, be consistent in the order in which cell types are collected from one image to the next, and one timepoint/genotype to the next. Currently, for 21dag hypocotyls:
(1)Red=vessels
(2)Green=parenchyma
(3)Blue=vascular cambium
(4)Yellow=phloem fibres
(5)Cyan=phloem
(6)Magenta=Cork
Add additional cell types after this, regardless of their position.

8. Choose which image you will import when you are prompted to do so. That image, along with the segmentation image (showing all cells as randomly coloured regions of interest) will load. The segmentation image is an aid to seeing which cells were correctly identified, and the image upon which your cell selections are indicated (by symbols unique to each cell class that you've designated.

9. Left click to make selections of cells for the first cell type; try to select about 20 cells of each category
-Choose ROIs that seem to be good descriptors of those cell types, but incorporating the full range of shapes and
positions associated with each cell type. Avoid cases of obvious over- or under-segmentation results. If you make an error in selecting an ROI, you can left-click on it again to remove it from the list, even if it was not the last one you selected.
-Work with an appropriate level of zoom to make accurate selections. Progress is slower, though, and it may be faster and sufficient for selecting some cell types to work at a zoomed out scale.
-Zoom in ('i') and out ('o') by selecting the image panel (CFW counterstain) rather than the segmented image.

10. Move on to the next cell type by right-clicking once. Confirm in the command line that 'click on object type [#]' appears and indicates the correct cell type number. Repeat steps 9 and 10 until all cell types are finished. The script will end and a file with the original filename.mat will be generated in the same directory as the source images. This file will be utilized in the classification script to define the learning set for SVM (or other).
All images should close, and two files should be generated:
-.MAT file containing the classification info for the selected ROIs, appended by the original .LSM filename
-.TIF file showing the original CFW channel overlaid with the cells shaded by category

Segmentation, Classification, Fluorescence Quantification Protocol

===

Overview

After the training set has been generated (see 'Training_set_protocol.txt'), the 'main.m' algorithm performs the following tasks;
1. Manual identification of the centers of the tissues in the target images using
 the 'generate_centers.m' algorithm
2. Regions of interest (ROIs) are generated for the experimental image set.
3. Features measures are generated from the training set
4. Training set image ROIs are loaded
5. Imports the list of ROIS chosen in the training set for each cell category (a subset of the total ROIs)

Materials

-LSM files for a given experimental set. They should be taken from the same experimental group sharing a common factor with the training set such as developmental age and tissue type.
-'main.m' and associated scripts in

Procedure

The main function calls all functions, and you need to run it.
All the paths which are used by other functions is in main function and you need to correct it accordingly.

1) The first step is to run generate_trainingset.m. This function reads images in the training folder, and generates features for the objects which are manually marked. The results are saved as .mat file are later used for the classification.
For generate_trainingset you must be careful that parameters are exactly the same as the parameters used before for manually detection.
It then calculates features. For feature calculation we need to have center of image. When you run generate_trainingset an image is pop up and you have to click on center, press enter to open the next image. It also does classification and the result is saved in the same folder that the training examples are.

2) generate_centers.m, saves the centre of images in the data directory. Click on the center and then press enter to go to the next image.

3) segmenation.m : segment ROIS

4) Features4ROI.m: generates features for every cells

5) classification: use the training set generated in the first step to train the classifier and then classifies the cells.

The result of every step is saved as .mat file. You need to run all the blocks of the algorithm once. For the second run if you for example want to change classification parameters and want to only run this part, you can go to the main_resume function and run the script up to classification part. 'main_resume' makes all variables from the previous steps available for the classification.
