# Supplementary material for: Precision Automation of Cell Type Classification and Sub-Cellular Fluorescence Quantification from Laser Scanning Confocal Images
Source: Front Plant Sci. 2016 Feb 9;7:119. doi: 10.3389/fpls.2016.00119 (PMC4746258; doi:10.3389/fpls.2016.00119)
Supplement: Supplementary file 3 [file DataSheet1.ZIP › 20151214_MatlabFiles/scripts/03_supplementary/libPLS_1.95/description_libPLS.pdf]

libPLS: an Integrated Library for Partial Least Squares Regression and Discriminant Analysis

Research Center of Modernization of Chinese Medicines, College of Chemistry and Chemical Engineering Central South University, Changsha 410083, P.R. China

Advisor: Professor Yi-Zeng Liang, yizeng_liang@263.net
Coder: Hong-Dong Li, lhdcsu@gmail.com
Date: Apr. 1, 2013.

Copyright & License

The libPLS software is supplied subject to version 2 of the "GNU General Public License". See the GNU General Public License for more details. libPLS can be freely available to all non-commercial users. It can be redistributed and/or modified under the terms of the GNU. The latest version and detailed documents are freely available at: www.libpls.net

Platform

The current version is coded and tested in MATLAB 7.10.0(R2010a). If any questions about running this software, please feel free to contact us.

If you use libPLS in your scientific work, please cite as:

[1] Li H.-D., Xu Q.-S., Liang Y.-Z. (2014) libPLS: An Integrated Library for Partial Least Squares Regression and Discriminant Analysis. PeerJ PrePrints 2:e190v1, source codes available at www.libpls.net.
