# Supplementary material for: Precision Automation of Cell Type Classification and Sub-Cellular Fluorescence Quantification from Laser Scanning Confocal Images
Source: Front Plant Sci. 2016 Feb 9;7:119. doi: 10.3389/fpls.2016.00119 (PMC4746258; doi:10.3389/fpls.2016.00119)
Supplement: Supplementary file 3 [file DataSheet1.ZIP › 20151214_MatlabFiles/UserDocs/2014-09-25_Pipeline_overview.pptx]

## Slide 1
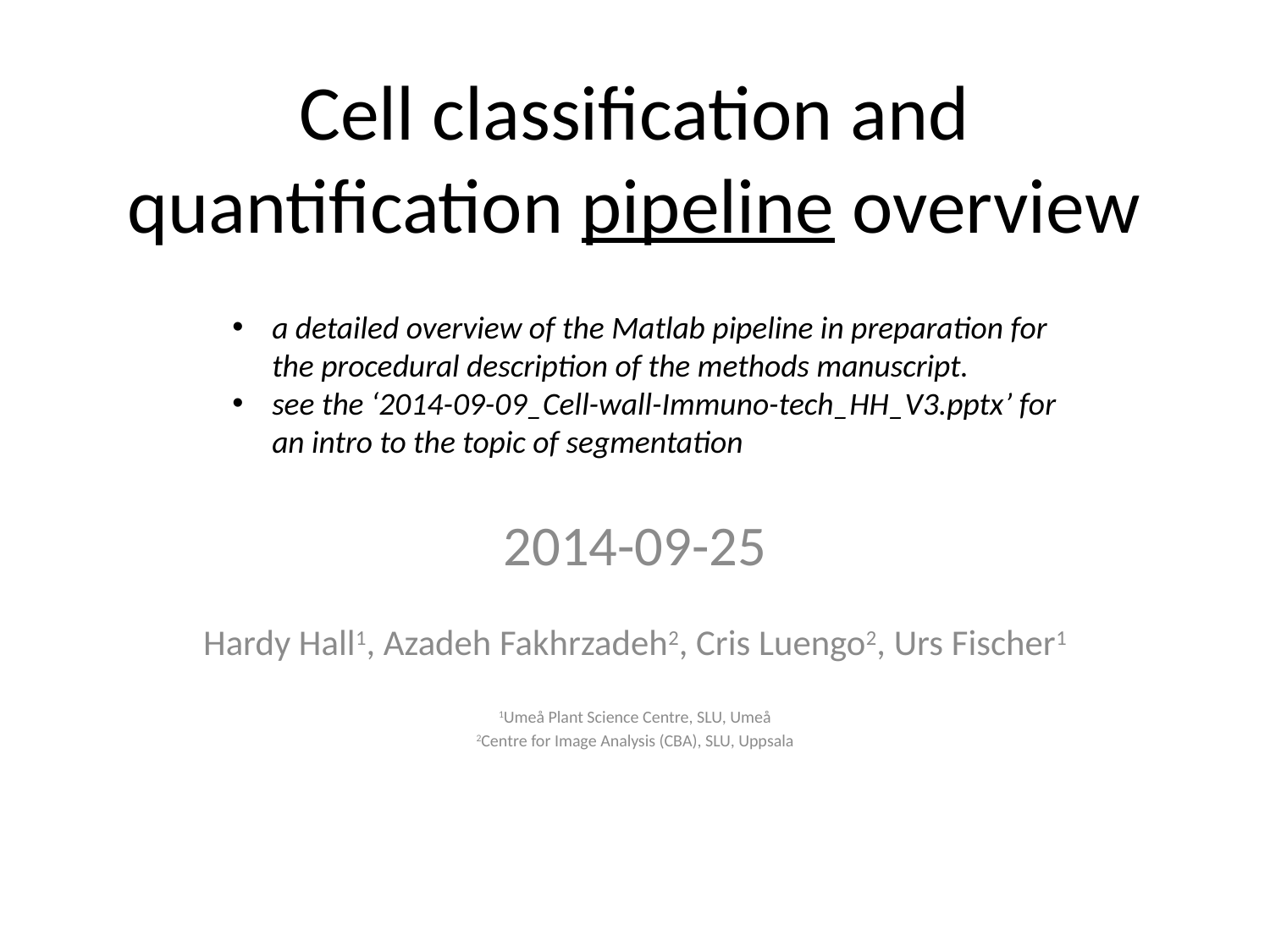

# Cell classification and quantification pipeline overview
a detailed overview of the Matlab pipeline in preparation for the procedural description of the methods manuscript.
see the ‘2014-09-09_Cell-wall-Immuno-tech_HH_V3.pptx’ for an intro to the topic of segmentation
2014-09-25
Hardy Hall1, Azadeh Fakhrzadeh2, Cris Luengo2, Urs Fischer1
1Umeå Plant Science Centre, SLU, Umeå
2Centre for Image Analysis (CBA), SLU, Uppsala

## Slide 2
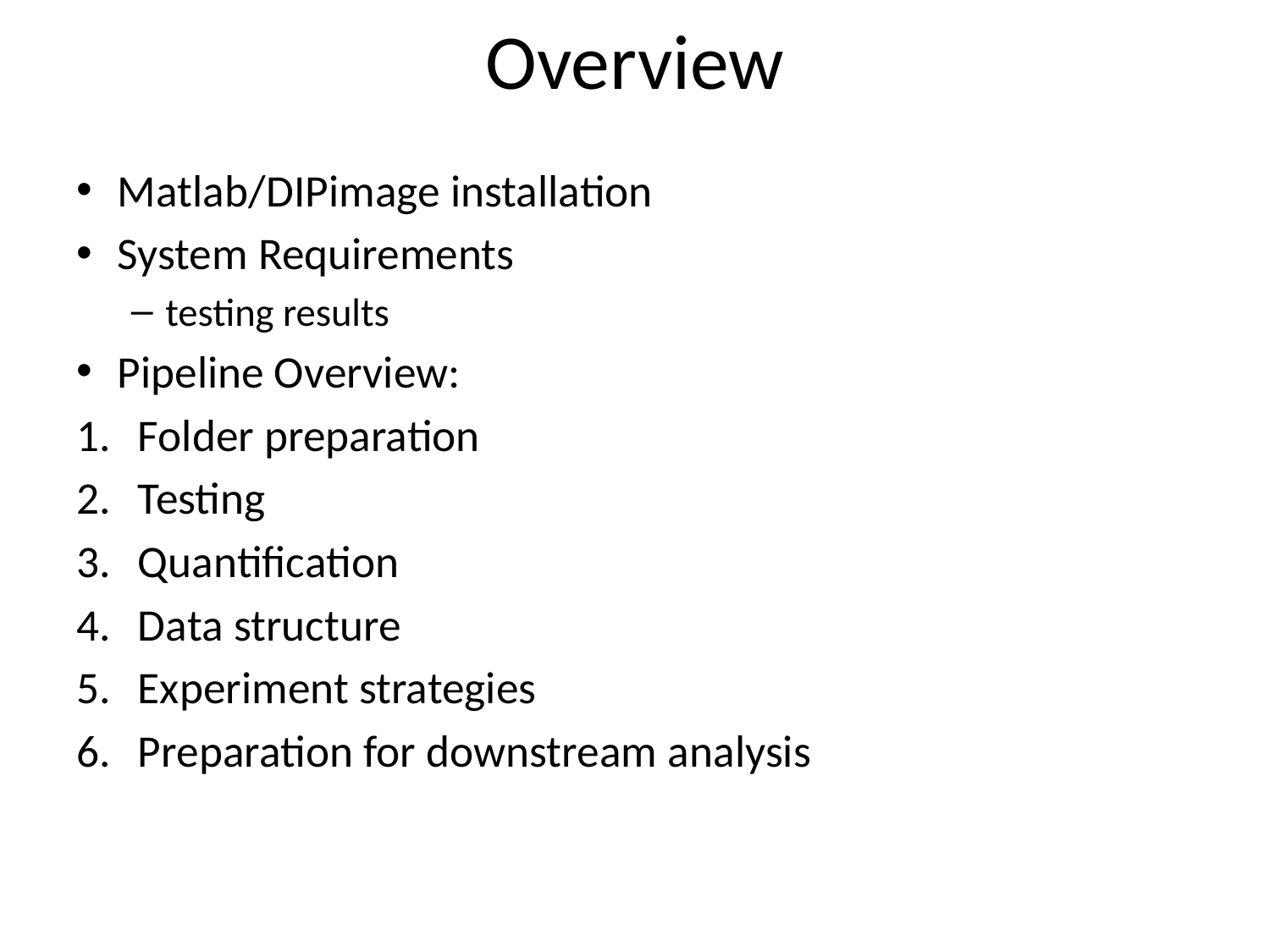

# Overview
Matlab/DIPimage installation
System Requirements
testing results
Pipeline Overview:
Folder preparation
Testing
Quantification
Data structure
Experiment strategies
Preparation for downstream analysis

## Slide 3
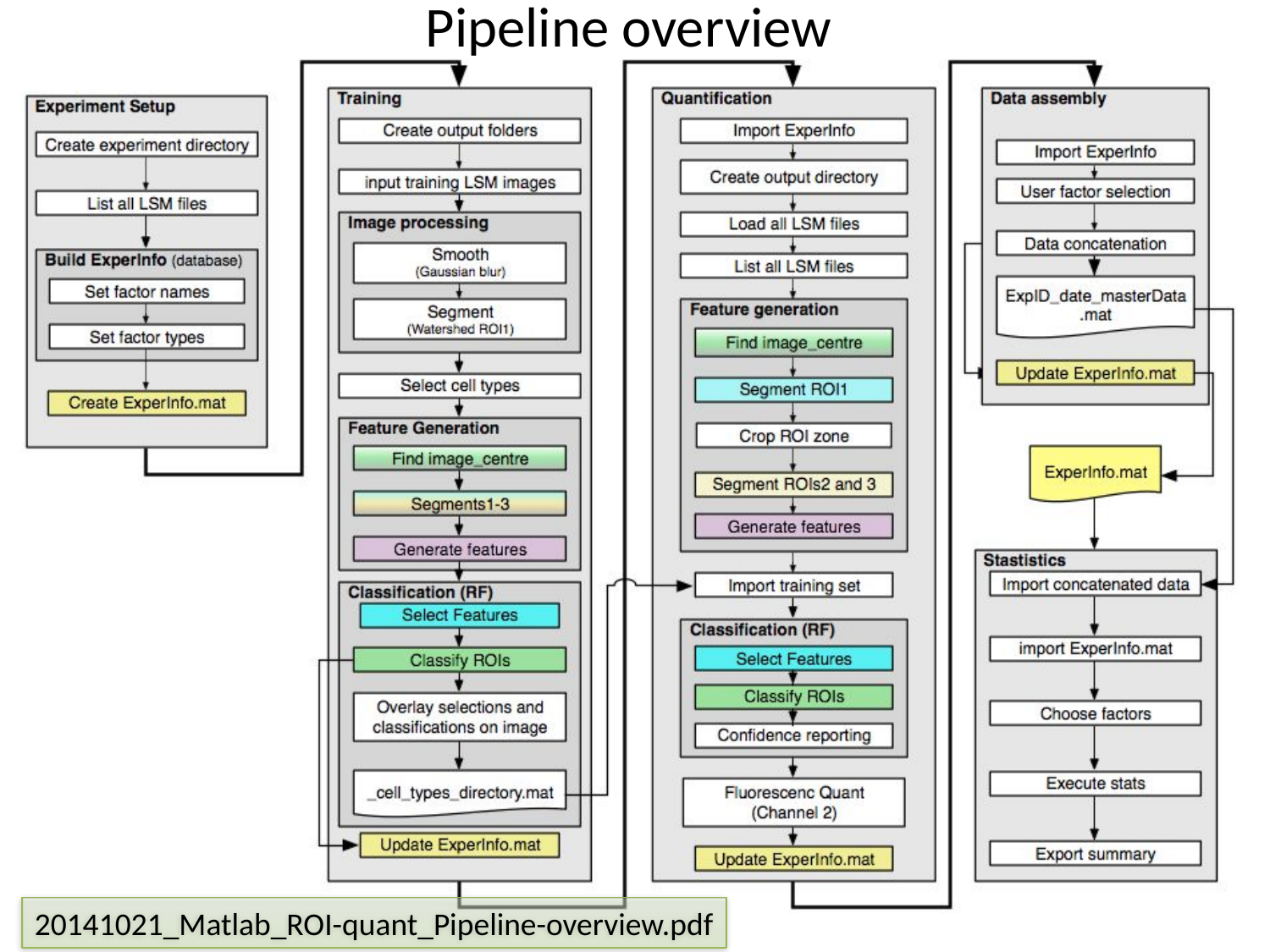

# Pipeline overview
20141021_Matlab_ROI-quant_Pipeline-overview.pdf

## Slide 4
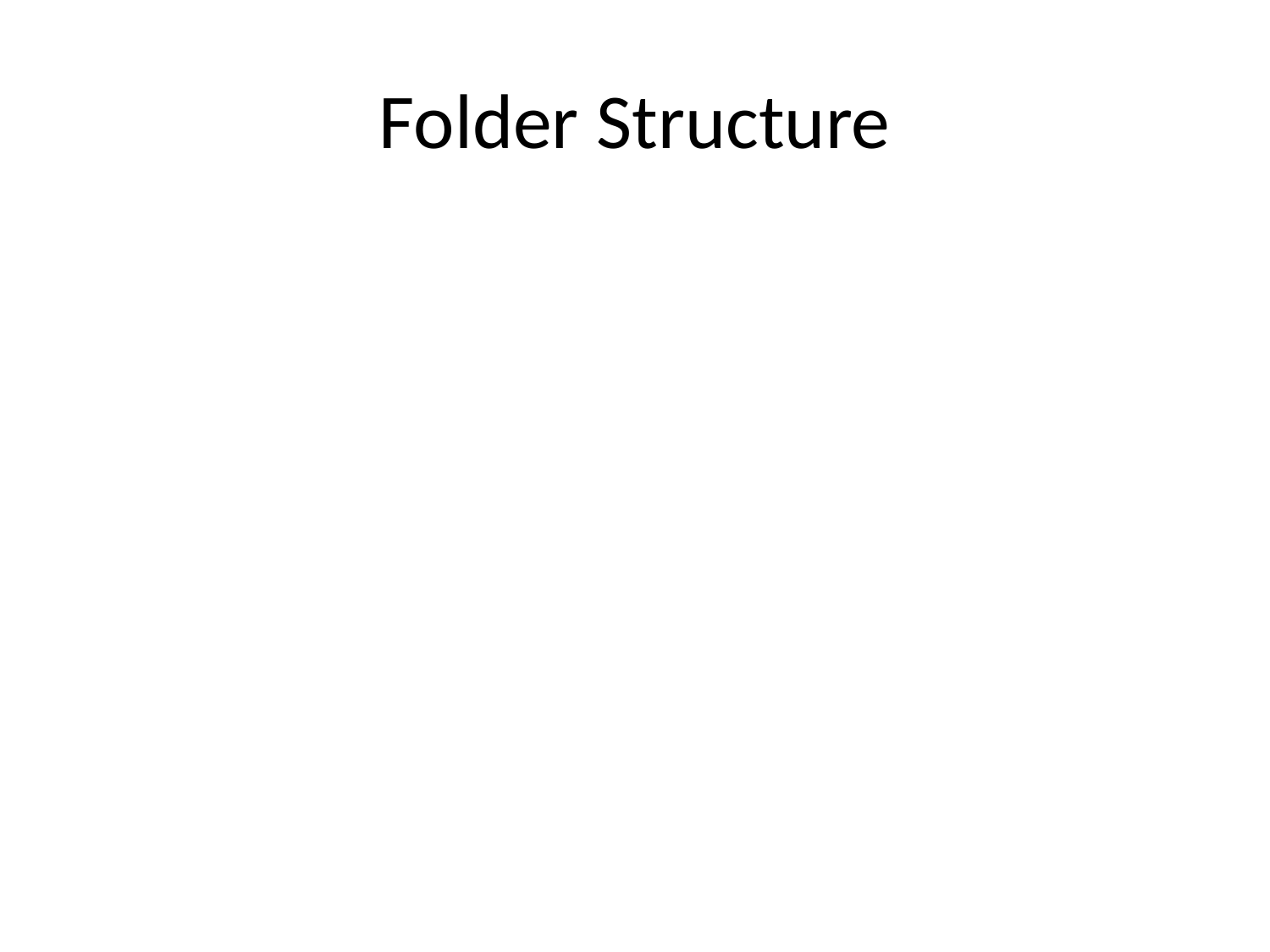

# Folder Structure

## Slide 5
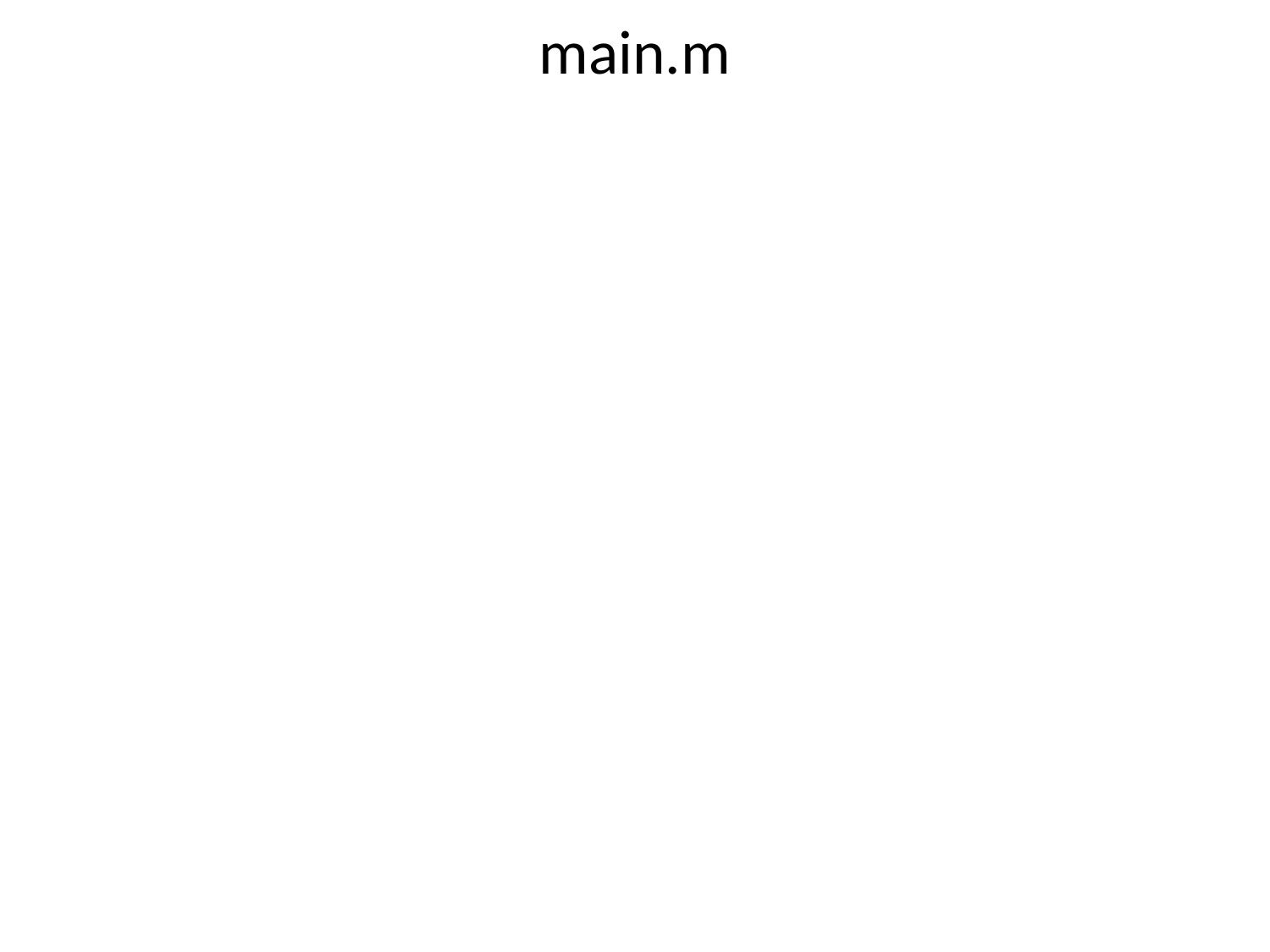

# main.m

## Slide 6
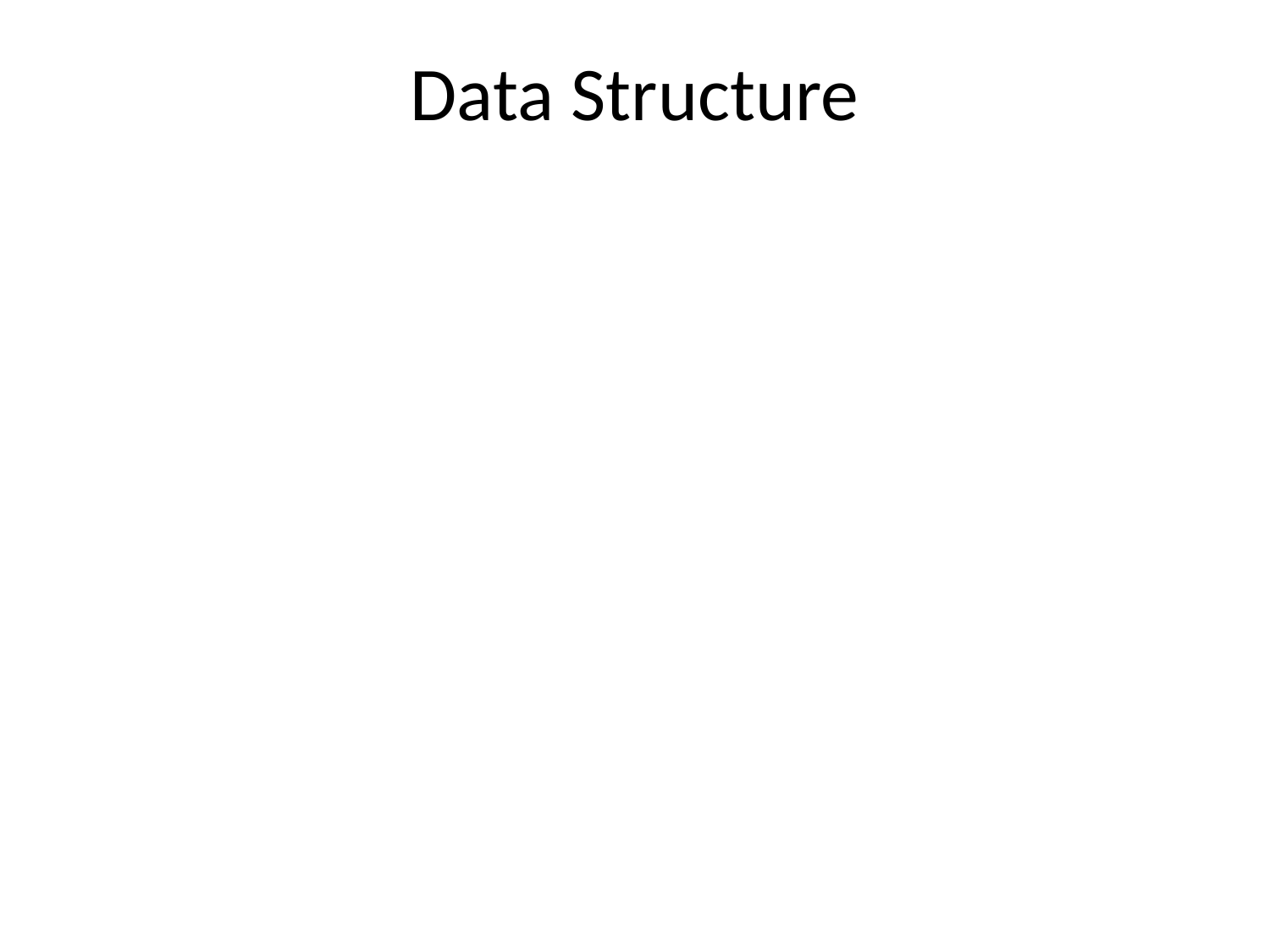

# Data Structure
