# Supplementary material for: Precision Automation of Cell Type Classification and Sub-Cellular Fluorescence Quantification from Laser Scanning Confocal Images
Source: Front Plant Sci. 2016 Feb 9;7:119. doi: 10.3389/fpls.2016.00119 (PMC4746258; doi:10.3389/fpls.2016.00119)
Supplement: Supplementary file 3 [file DataSheet1.ZIP › 20151214_MatlabFiles/UserDocs/dipimage_user_manual.pdf]

DIP*image* User Manual

dr. ir. Cris L. Luengo Hendriks
prof. dr. ir. Lucas J. van Vliet
dr. dipl. phys. Bernd Rieger
dr. ir. Michael van Ginkel
ing. Ronald Ligteringen

Quantitative Imaging Group,
Department of Applied Sciences,
Delft University of Technology

Delft
May 9, 2014

Contents

1 Introduction **1**

1.1 The DIP*image* toolbox . . . 1
1.2 The DIP*lib* library . . . 1
1.3 Image Processing . . . 2
1.4 Documentation Conventions . . . 2
1.5 Acknowledgments . . . 2

2 Installing DIP*image* **3**

2.1 Windows Installation . . . 3
2.1.1 Automatic Installation . . . 3
2.1.2 Manual Installation . . . 4
2.2 UNIX Installation . . . 4
2.3 MacOS X Installation . . . 5

3 Getting Started **7**

3.1 Starting the GUI . . . 7
3.2 Loading and Displaying an Image . . . 7
3.3 Pre-processing the Image . . . 8
3.4 Measuring . . . 10
3.5 Where to Go from Here . . . 11

4 The `dip_image` Object **12**

4.1 Creating a `dip_image` Object . . . 12
4.2 Displaying `dip_image` Objects . . . 13
4.3 Operations on `dip_image` Objects . . . 13
4.4 Dimensions . . . 15
4.5 Indexing Pixels . . . 15
4.6 Image Arrays . . . 16
4.7 Tensor Images . . . 17
4.8 Color Images . . . 18
4.9 A Note on the `end` Method in Indexing . . . 18
4.10 Special Functions . . . 19
4.11 Review of the Differences Between a `dip_image` and a MATLAB Array . . . 24

5 The `dip_measurement` Object **26**

5.1 Extracting Measurement Data . . . 26
5.2 Other Information on the `dip_measurement` Object . . . 26
5.3 Combining Measurement Data . . . 27
5.4 Adding Measurement Data . . . 27
5.5 Converting a `dip_measurement` Object to a `dataset` Object . . . 28

5.6 Creating a `dip_measurement` Object with Your Own Data 28
5.7 Backwards Compatibility 28

6 Figure Windows **29**
6.1 The Figure Window Menus 29
6.2 Using the Mouse in Figure Windows 30
6.3 Using the Keyboard in Figure Windows 31
6.4 Linking Variables with Figure Windows 32
6.5 Setting the Position of Figure Windows 32

7 Toolbox Functions **34**
7.1 The GUI: `dipimage` 34
7.2 The `dipshow` Function 34
7.3 Figure Window Support: `dipmapping` 35
7.4 Figure Window Support: `diptruesize` 36
7.5 Figure Window Support: `diptest`, `dipzoom`, *et al.* 36
7.6 Figure Window Support: `diplink` 37
7.7 Creating, Linking and Clearing Figure Windows: `dipfig` and `dipclf` 37
7.8 Toolbox Preferences: `dipsetpref` and `dipgetpref` 38
7.9 Interactive Tools: `dipcrop`, `dipgetcoords`, *et al.* 38
7.10 Other 3D Visualization Tools: `dipanimate`, `dipisosurface`, `dipprojection` 38
7.11 Image Processing Functions 38
7.12 Adding Functions to the GUI 39
7.13 Automatic Parameter Parsing 44

8 Customizing the DIP*image* Environment **46**
8.1 Figure Windows 46
8.2 Graphical user Interface 46
8.3 Initialization File 47
8.4 Other Settings 47

9 Low-level DIP*lib* Interface **55**
9.1 The Setup 55
9.2 Calling DIP*lib* Functions 55
9.3 Example Function Call 56

10 DIP*image* and the Matlab Compiler **58**
10.1 The MATLAB Compiler 58
10.2 Compiling an M-file that uses DIP*image* 58
10.3 Deploying your compiled program 59

Chapter 1

Introduction

1.1 The DIP*image* toolbox

MATLAB is a software package designed for (among other things) data processing. It contains a huge amount of numerical algorithms, and very good data-visualization abilities. This makes it adequate for image processing. However, MATLAB's virtues do not end there. It is also an ideal tool for rapid prototyping, since it handles a compact but simple notation and it is very easy to add functions to it. The drawback is that MATLAB, since it is an interpreted language, is slow for some constructs like loops; it also is not very efficient with memory (for example, all MATLAB data uses 8-byte floats). This makes it a bit less useful beyond the prototyping stage.

DIP*image* is a MATLAB toolbox for doing image processing, and is based on the image-processing library DIP*lib*. It is meant as a tool for research as well as teaching image processing at various levels. It is not meant as an industrial image-processing package, which should heavily depend on speed and memory-efficiency. Instead, this toolbox is made with user-friendliness, ease of implementation of new features, and compactness of notation in mind.

Most arithmetic operations are done by MATLAB. However, implementing image-processing filters usually requires several nested loops (depending on the dimensionality of the input data), which is not very efficient. Therefore, we based this toolbox on DIP*lib*. It provides all filtering and transform functions and is the heart of the DIP*image* toolbox.

1.2 The DIP*lib* library

DIP*lib* is a scientific image-processing library written in C. It contains a large number of functions for processing and analyzing multi-dimensional image data. The library provides functions for performing transforms, filter operations, object generation, and statistical analysis of images. It is also very efficient (with both memory and time).

The MATLAB interface to DIP*lib* is a simple "glue" layer, which allows calling the C functions in the library by converting the MATLAB data to a form used by the library. Only a few functions have added functionality in the interface. Using these functions therefore is much like using the C functions directly. This is not adequate for the beginning image analyst, who is better off using the DIP*image* toolbox functions instead.

More information on DIP*lib* can be found at:
http://www.diplib.org/.

1.3 Image Processing

This manual is meant as an introduction and reference to the DIP*image* toolbox, not as a tutorial on image processing. Although Chapter 3 shows some image-processing basics, it is not complete. We refer to "The Fundamentals of Image Processing", an online image-processing course, which can be found at:
ftp://ftp.tudelft.nl/pub/DIPimage/docs/FIP2.3.pdf.

1.4 Documentation Conventions

The following conventions are used throughout this manual:

- Example code: in `typewriter` font
- File names and URLs: in `typewriter` font
- Function names/syntax: in `typewriter` font
- Keys: in **bold**
- Mathematical expressions: in *italic*
- Menu names, menu items, and controls: "inside quotes"
- Description of incomplete features: in *italic*

1.5 Acknowledgments

DIP*lib* was written mainly by Michael van Ginkel, Geert van Kempen, Cris Luengo and Lucas van Vliet; the MATLAB interface to DIP*lib* was written by Cris Luengo, with help from Michael van Ginkel. Bernd Rieger, Tuan Pham, Kees van Wijk and Frank Faas have contributed functionality.

The DIP*image* toolbox was written mainly by Cris Luengo, Lucas van Vliet, Bernd Rieger and Michael van Ginkel. Tuan Pham, Kees van Wijk, Judith Dijk, Geert van Kempen and Peter Bakker have contributed functionality.

DIP*lib* and the DIP*image* toolbox are being developed at the Quantitative Imaging Group, Delft University of Technology. Lucas van Vliet is the project supervisor.

Chapter 2

Installing DIP*image*

This toolbox requires MATLAB 5.3/R11 or later, though the official distributions are compiled with later versions of MATLAB and do not run on earlier versions. The download page on the DIP*image* web site should specify the versions of MATLAB a specific distribution is compatible with: http://www.diplib.org/download Some functionality is only available on later versions of MATLAB, but this is an exception.

The bulk of the toolbox is platform-independent. This means that the distributions for the various platforms contain exactly the same functionality, with very few exceptions. However, you do need to obtain the distribution specific to your platform, because the toolbox contains code compiled to work on a specific platform. You can install the same version of the toolbox for different platforms to the same directory. The files that have the same name in the various distributions are identical (assuming they are of the same release!), the platform-specific files have unique names and paths. For example, on Windows you can install both the 32-bit and 64-bit versions of the toolbox into the same directory.

2.1 Windows Installation

2.1.1 Automatic Installation

If you have an earlier version of DIP*image* installed, it is important that you remove it before installing this version. Previous versions installed with the automatic installation tool can be uninstalled through the program management tool in Windows. A previous version installed manually should be uninstalled by either deleting or renaming the whole directory, and undoing any other changes made during installation.

To install DIP*image*, simply run the installation program and follow the directions in it. The tool will tell you to start MATLAB and type the command

```
run('C:\Program Files\dip\dipstart.m')
```

where `C:\Program Files\dip\` is the directory to which you installed DIP*image*. The script `dipstart.m`, executed this way, contains three commands needed to initialise the toolbox (two if you didn't install the images). These must be executed every time you start MATLAB. You can modify (or create) a file `startup.m` in the directory to which MATLAB starts up, to contain the `run` command above. The script `startup.m` is executed automatically every time MATLAB starts.

Optionally, you can add the demo directory to your path too:

```
addpath C:\dip\common\dipimage\demos
```

It contains a few demos that show how to use different features of the toolbox.

2.1.2 Manual Installation

If you have an earlier version of DIP*image* installed, it is important that you remove it before installing this version. Previous versions installed with the automatic installation tool can be uninstalled through the program management tool in Windows. A previous version installed manually should be uninstalled by either deleting or renaming the whole directory, and undoing any other changes made during installation.

Unzip the distribution file `dipimage_X.X_XXX_winXX.zip` to any destination (say, `C:\`). It will generate a directory `C:\dip` with two sub-directories: `C:\dip\common\` and `C:\dip\win32\` or `C:\dip\win64\`, depending on the version you downloaded. The first one contains the MATLAB toolbox, the second one the DIP*lib* library files, support libraries and include files.

You also might want to unzip the images file `images.zip` to the `C:\dip` directory. It will create a directory `C:\dip\images\` with some default images.

To start using DIP*image*, do the following in MATLAB:

```
addpath('C:\dip\common\dipimage')
dip_initialise
dipsetpref('imagefilepath','C:\dip\images')
```

(make sure to substitute `'C:\dip\'` for the name of the directory where you unzipped the distribution file). This will add the DIP*image* toolbox and low-level DIP*lib* interface to the beginning of the path, initialize DIP*lib* and set the default directory where DIP*image* will look for images.

You can add these lines to your `startup.m` file, which should be in your working directory. That way, DIP*image* will be ready to use each time you start MATLAB.

Optionally, you can add the demo directory to your path too:

```
addpath C:\dip\common\dipimage\demos
```

It contains a few demos that show how to use different features of the toolbox.

Note: On versions of MATLAB since version 7.2, it is no longer necessary for Windows to be able to find the DIP*lib* library files (unless you are creating a stand-alone executable using DIP*lib*, but that's a different topic altogether). If you have a version of MATLAB that is older than 7.2, and happen to find a version of DIP*image* that works with your version of MATLAB (these are no longer supported), you will need to modify the `PATH` environment variable to contain the `C:\dip\win32\lib\` or (`C:\dip\win64\lib\`) directory. You must modify this environment variable before starting MATLAB, and possibly must restart Windows for this change to take effect.

2.2 UNIX Installation

If you already have a version of DIP*image* installed, rename the directory it is in, so that you will still have the old version if the installation of the new version fails. Untar the distribution file. It will create a directory `dip/` with a number of subdirectories. If you untarred the file in the directory `/something/`, you now have a directory `/something/dip/`. To get DIP*image* running, there are a number of things that you must do:

1. MATLAB must be told where it can find DIP*lib*, a shared library, that is used by

DIP*image*. You can do this by creating the environment variable `LD_LIBRARY_PATH`, or extending it if it already exists. As the name suggests, it holds a collection of paths. The entries are separated by colons, i.e. '`:`'. Which entry you should add, depends on the type of machine you are working on:

- On Solaris add: `/something/dip/SunOS/lib/:/something/dip/SunOS32/lib/`
- On Linux add: `/something/dip/Linux/lib/`
- On 64-bit Linux add: `/something/dip/Linuxa64/lib/`

2. If you did not do so before: download the separate `tar` or `zip` file containing some test images. Install them somewhere on your system. Let us assume that you installed them in `/herebeimages/`.
3. Add the following lines to your `startup.m` (preferably in `$HOME/matlab/`):

```
addpath('/something/dip/common/dipimage')
dip_initialise
dipsetpref('imagefilepath','/herebeimages')
```

This will add the DIP*image* toolbox and low-level DIP*lib* interface to the beginning of the path, initialize DIP*lib* and set the default directory where DIP*image* will look for images.

Optionally, you can add the demo directory to your path too:

```
addpath /something/dip/common/dipimage/demos
```

It contains a few demos that show how to use different features of the toolbox.

2.3 MacOS X Installation

If you already have a version of DIP*image* installed, rename the directory it is in, so that you will still have the old version if the installation of the new version fails. Extract the distribution file. It will create a directory `dip/` with a number of subdirectories. We suggest you place this directory in `/Applications/`, so that you now have a directory `/Applications/dip/`. To get DIP*image* running, there are a number of things that you must do:

1. Create a file `/etc/launchd.conf` with the following line (or add the following line if you already have this file):

```
setenv DYLD_LIBRARY_PATH /Applications/dip/Darwin/lib/
```

This is a plain text file, and it is hidden, so you need to create it through the Terminal or an application that allows creating and editing hidden files. You need to have root privileges to create and edit it. A simple way of making this file is to type at the Terminal promt:

```
sudo bash -c "echo setenv DYLD_LIBRARY_PATH
     /Applications/dip/Darwin/lib/ >> /etc/launchd.conf"
```

(This should all be on the same line.)

If you already had defined the `DYLD_LIBRARY_PATH` environment variable in `/etc/launchd.conf`, you can add this new directory using the colon (":") to separate it from the other directory.

Next, either reboot or type the following command in Terminal:

```
launchctl < /etc/launchd.conf; sudo launchctl < /etc/launchd.conf
```

2. If you did not do so before: download the separate `tar` or `zip` file containing some test images. Install them somewhere on your system. Let us assume that you installed them in `/Applications/dip/images/`.
3. Add the following lines to your `startup.m` (preferably in `$HOME/matlab/`):

```
addpath('/Applications/dip/common/dipimage')
dip_initialise
dipsetpref('imagefilepath','/Applications/dip/images')
```

This will add the DIP*image* toolbox and low-level DIP*lib* interface to the beginning of the path, initialize DIP*lib* and set the default directory where DIP*image* will look for images.

Optionally, you can add the demo directory to your path too:

```
addpath /something/dip/common/dipimage/demos
```

It contains a few demos that show how to use different features of the toolbox.

Chapter 3

Getting Started

To show you around DIP*image*, we will work through a simple image-processing application. Not all steps will be written out explicitly, since it is our goal to make you understand what is going on, and not to have you copy some commands and stare in amazement at the result.

The goal of this application is to do some measurements on an image of some rice grains, then analyze these measurements.

3.1 Starting the GUI

Type the following command at the MATLAB prompt:

```
dipimage
```

This should start the DIP*image* GUI. A new window appears to the top-left of the screen, which contains a menu bar. Spend some time exploring the menus. When you choose one of the options, the area beneath the menu bar should change into a dialog box that allows you to enter the parameters for the filter you have chosen. See also Sections 7.1 and 8.2 for more info on the GUI.

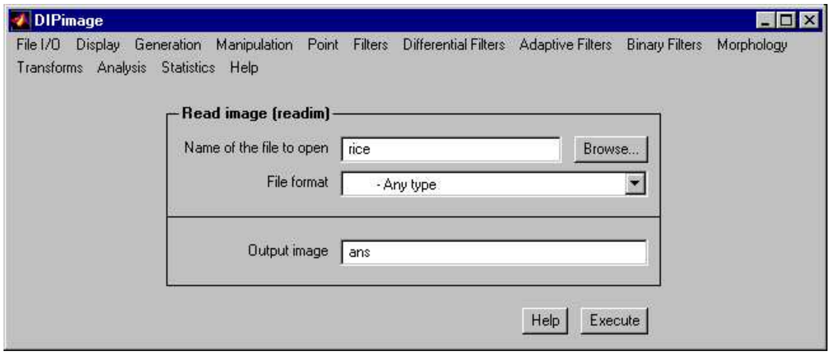

3.2 Loading and Displaying an Image

Before you can use these functions, you first need to load some image. The first menu is called "File I/O", and its first item "Read image (readim)". Select it. Press the "Browse" button, and choose the file `rice.tif`. Change the name for the output variable from ans to `a`, then press the "Execute" button. Two things should happen:

1. The image 'rice' is loaded into the variable `a`, and displayed to some figure window:

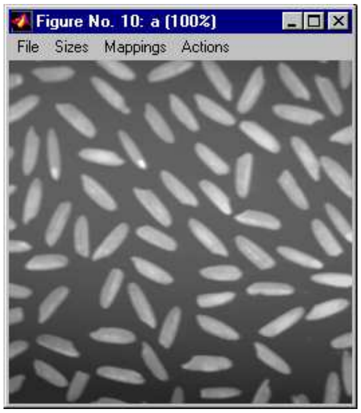

2. The following lines (or something very similar) appear in the command window:

```
>> a = readim('c:\matlab\toolbox\dipimage\images\rice.tif','')
Displayed in figure 1
```

This is to show you that the same would have happened if you would have typed that command directly yourself, without using the GUI. Try typing this command:

```
a = readim('rice')
```

The same image should be loaded into the same variable, and again displayed to some window. Note that we left off the '.tif' ending of the filename. `readim` can find the file without you having to specify the extension. We also didn't use the second argument to the `readim` function, since '' is the default value. Finally, by not specifying a path to the file, we asked the function to look for it either in the current directory or in any of the directories specified by the `ImageFilePath` setting (see Section 8.4).

To avoid having the image displayed in a window automatically, add a semicolon to the end of the command:

```
a = readim('rice');
```

3.3 Pre-processing the Image

You will have noticed the heavy background shading in this image. If we try to segment it directly, the results will be unsatisfactory (as you can try out later). Let's do some background correction. The idea is to use a low-pass filter that removes the objects while keeping the slow change in the background. Choose "Filters" and "Gaussian filter". Select `a` as the input image, and choose a name for your background image (we use `bg`). Finally, choose a suitable value for the filter parameter, such that the objects are removed and the background shading is left. Try different settings until you are satisfied with the result.

Once we have the background image, we can subtract it from the original image. It is very easy to do arithmetic with images in MATLAB. Type

```
a = a - bg
```

The new image should be displayed to a figure window, but it looks very dark. This is because the pixels have lower values now, some even have negative values. By default, images are displayed by mapping the value 0 to black, and the value 255 to white. You can change

this by choosing a different mapping mode. Open the "Mappings" menu on the figure window, and choose "Linear stretch" (try out the other modes too).

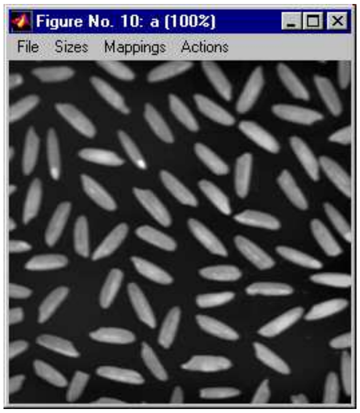

The "Actions" menu allows you to choose what the mouse should do on the figure window. Select "Pixel testing", and press the mouse button while pointing somewhere in the image (keep the button down). The figure caption changes to show the coordinates of the mouse in the image and the value of the pixel at those coordinates. Try moving the mouse while holding the button down. Another option on the "Actions" menu ("Zoom") is used to zoom in on an image. Try it out too. See Chapter 6 for more information on the figure windows.

The next step is to segment the image. We need to find some threshold that distinguishes the grains of rice from the background. To find it, we can examine the histogram of the image. Choose "Histogram" on the "Statistics" menu, or type

```
diphist(a,[])
```

The graph shows two peaks, one for the background, one for the objects. Find a value in between for the threshold. To do the segmentation, compare all pixel values with the threshold, which can be done in this way:

```
b = a > 20
```

This results in a binary image (logical image, containing values of "true" and "false", coded as 1 and 0), with ones at the pixels that belong to the objects. This image is displayed in red and black to emphasize that it is a binary image rather than a grey-value image with only two different grey values. Binary images have different characteristics than grey-value images, for example they can be used to index into other images, just like MATLAB's logical arrays.

The final step is to remove the grains that do not completely lie inside the image. We can do this using a binary operation. Find and execute the "Remove edge objects" function in the menu system. What it does is the same as the `bpropagation` function, with an empty image as a seed image, and the edge condition set to 1. To create an empty seed image use the `newim` function. Thus, these two commands are equivalent:

```
b = b - bpropagation(newim(b,'bin'),b,0,2,1)
b = brmedgeobjs(b,2)
```

3.4 Measuring

Before we can start measuring, it is convenient to have a label image. Select the "Label objects" item on the "Segmentation" menu, and select the new object image as the input. The result (name the image `lab`) is a labeled image where the pixels belonging to each object have a different value. In the window of the new image, select the "Labels" mapping. Now each grey value gets a different color. Examine the pixel values to see how the objects are labeled.

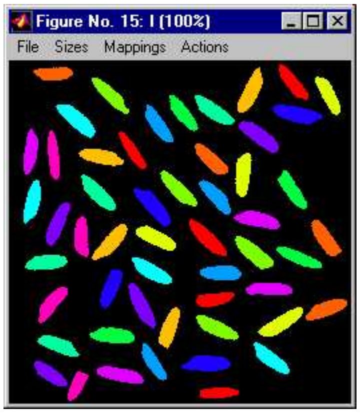

Now do the measuring. We will measure the object area in pixels (`'size'`) and the Feret diameters (`'feret'`), which are the largest and smallest diameters, and the diameter perpendicular to the smallest diameter.

```
data = measure(lab,[],{'size','feret'});
```

`measure` returns an object of type `dip_measurement`, which is explained further in Chapter 5. Leaving the semi-colon off the previous command, the complete measurement results are displayed at the command prompt. Furthermore, `data(1)` is the measurement results for object with label 1, `data.feret` is an array containing all the Feret diameters, and `data(1).feret` are the Feret diameters for object number 1.

To extract the measurements done on all objects and put them in an array, type

```
feret = data.feret;
sz = data.size;
```

This gives us arrays with the measured data. MATLAB allows all kinds of statistics and analysis on these arrays. For example, `mean(sz)` gives the mean grain area.

We will use `scatter` to find some correlation between the diameters and the area of the grains. Let's start by plotting the length of the grains against their width:

```
figure; scatter(feret(1,:),feret(2,:))
```

Apparently, they are mostly unrelated. Let's try a relation between the length and the surface area:

```
scatter(feret(1,:),sz)
```

These appear to be more related, but, of course, the area also depends on the width of the grains. If we assume that the grains are elliptic, we know that the area is $\frac{1}{4}\pi d_1 d_2$. Let's plot

the calculated area against the measured area:

```
scatter(sz,pi*feret(1,:).*feret(2,:)/4)
```

Wow! That is a linear relation. We can add a line along the diagonal to see how much the ratio differs from 1 (the other commands are to make the figure look prettier):

```
hold on , plot([180,360],[180,360],'k--')
axis equal , box on
xlabel('object area   (pixels^2)')
ylabel('\pi{\cdot}a{\cdot}b   (pixels^2)')
```

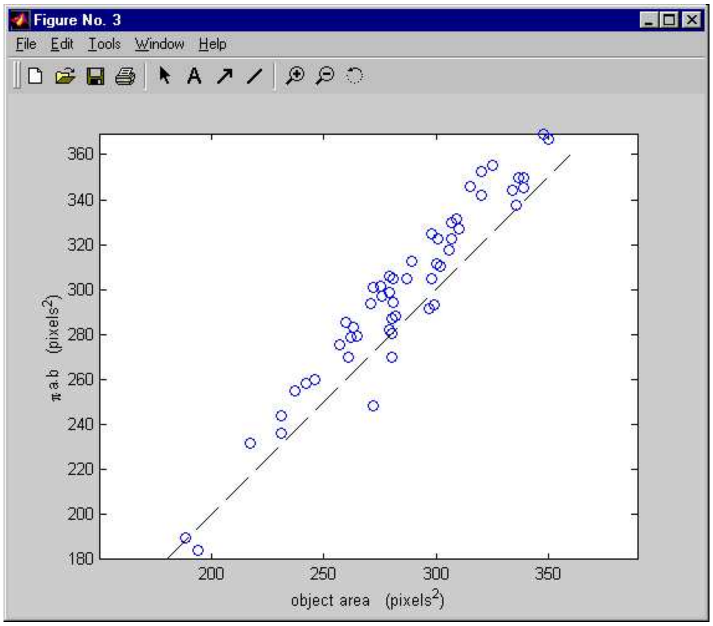

The actual slope can be computed by:

```
f = sz'\calc'
```

(this is the lest-squares solution to the linear equation `sz'*f = calc'`; the apostrophes transpose the vectors to create column vectors).

3.5 Where to Go from Here

If you are new to MATLAB, it would be a good idea to read the "Getting Started with MATLAB" manual. If you are new to image processing, you can read "The Fundamentals of Image Processing", an online image-processing course, which can be found at: ftp://ftp.tudelft.nl/pub/DIPimage/docs/FIP2.3.pdf.

Before you start using this toolbox, we recommend that you read Chapter 4 (at least Section 4.11). It contains very important information on the `dip_image` object and its usage. Since it is not the same as a regular MATLAB array, it can be a bit confusing at first.

Chapter 4

The dip_image Object

Images used by this toolbox are encapsulated in an object called `dip_image`. Objects of this type are unlike regular MATLAB arrays in some ways, but behave similarly most of the time. This chapter explains the usage of these objects.

4.1 Creating a dip_image Object

To create a `dip_image` object, the function `dip_image` must be used. It converts any numeric array into an image object. The optional second argument indicates the desired data type for the image. The pixel data will be converted to this type if possible, or else an error will be generated (for example, it is illegal to convert complex data to a real type, since there are many ways this can be accomplished; it is necessary to do this explicitly). The valid data types are listed in Table 4.1. This table also lists some alternative names that are mapped to the names on the left; these are just to make specifying the data type easier.[1]

For example,

```
a = dip_image(a,'sfloat');
```

will convert the data in `a` to `single` (4-byte) floats before creating the `dip_image` object. The variable `a` now behaves somewhat differently than you might be used to. The following sections explain its behavior.

To convert a `dip_image` object back to a MATLAB array use the function `dip_array`. It simply returns the data array stored inside the `dip_image` object. The functions `double`, `single`, `uint8`, etc. convert the `dip_image` object to a MATLAB array of the specified class.

There are also some commands to create an image from scratch. `newim` is equivalent to the `zeros` function, but returns a `dip_image` object.

```
a = newim(256,256);
```

creates an image with 256x256 pixels set to zero. An additional parameter (as in Table 4.1) can be used to specify the data type of the new image. The default is `'sfloat'`. If `b` is an object of type `dip_image`, then

```
a = newim(b);
```

creates an image of the same size (this is the same as `newim(size(b))`). The functions `xx`, `yy`, `zz`, `rr` and `phiphi` all create an image containing the coordinates of its pixels, and can be used in formulas that need them. For example, `rr(256,256)<64` creates a binary image

[1] Note that these are the names of some additional DIP*lib* data types not used under MATLAB, the names MATLAB uses for the data types, and some generalizations of the other names.

Table 4.1: Valid data types for the dip_image object.

Name	Description	Other allowed names
bin	binary (in 8-bit integer)	bin8, bin16, bin32
uint8	8-bit unsigned integer	
uint16	16-bit unsigned integer	
uint32	32-bit unsigned integer	uint
sint8	8-bit signed integer	int8
sint16	16-bit signed integer	int16
sint32	32-bit signed integer	int, int32
sfloat	single precision float	float, single
dfloat	double precision float	double
scomplex	single precision complex	
dcomplex	double precision complex	complex

with a disk of radius 64. The expression

```
a = (yy('corner'))*sin((xx('corner'))^2/300)
```

generates a nice test pattern with increasing frequency along the x-axis, and increasing amplitude along the y-axis. All these functions have 256x256 pixels as the default output size, and allow as a parameter either the size of an image, or an image whose size is to be copied. For example, `a*xx(a)` is an image multiplied by its x-coordinates.

4.2 Displaying dip_image Objects

When a MATLAB command does not end with a semicolon, the display method is called for the resulting values, if any. This method defaults to calling the `disp` method, which displays all the values in matrices. For the `dip_image` objects, the display method has been overloaded to call `dipshow` instead; `dipshow` displays the image in a figure window (see Section 7.2 for more information on this function). Before display, `dipshow` first calls `squeeze` (see Sections 4.4 and 4.5), meaning that a 4x1x6 image will be displayed as if it were a 4x6 image.

The `disp` method shows only the image size and data type instead. If you want `display` to call `disp` instead of `dipshow`, you can change the `'DisplayToFigure'` preference using `dipsetpref` (see Sections 7.8 and 8.4).

For images that cannot be displayed by `dipshow`, (e.g. zero-dimensional and empty images, image arrays, etc.), `display` always calls `disp`.

4.3 Operations on dip_image Objects

All mathematical operations have been overloaded for the `dip_image` object. The matrix multiplication (`*`), which is meaningless on images, does a pixel-by-pixel multiplication, just as the array multiplication (`.*`). The same applies to the other matrix operations. Relational operations return binary images. Binary operations on non-binary images treat any non-zero value in those images as true and zero as false. For example, to do a threshold we do not

Table 4.2: Arithmetic functions defined for objects of type dip_image(image in, image out).

abs	acos	and, &	angle	asin	atan
atan2	besselj	ceil	complex	conj	cos
erf	exp	fix	floor	hypot	imag
log	log10	log2	mod	not, ~	or, |
phase	pow10	pow2	real	round	sign
sin	sqrt	tan	xor	-	+
*	.*	./	/	^	.^
==	~=	>	>=	<	<=

Table 4.3: Arithmetic functions defined for objects of type dip_image (image in, scalar out).

all	any	max	mean	median	min
percentile	prod	std	sum	var	

need a special function, since we have the relational operators:

```
b = a > 100;
```

A double threshold would be (note MATLAB's operator precedence):

```
b = a > 50 & a < 200;
```

When the two images in the operation do not have the same number of dimensions, images are expanded to match each other. This is called *singleton expansion*. For example, if image a is 10x12x15, and image b is 10x12, then image b is expanded along the third dimension by replication to compute a+b, resulting in an image the same size as a. If image a is 10x1, and image b is 1x12, the result of a+b is 10x12. Only dimensions of size one (and non-existing dimensions) will be expanded. If image a is 10x12, and image b is 1x6, a+b will produce an error.

A note is required on the data types of the resulting images. The "higher" data type always determines this result, but we have chosen never to return an integer type after any arithmetic operation. Thus, adding two integer images will result in a 4-byte floating-point image; an 8-byte floating-point (double) image is returned only if any of the two inputs is double. This can be changed by setting the 'KeepDataType' option, see Section 8.4.

Many of the arithmetic functions have also been defined for objects of type dip_image (see Tables 4.2 and 4.3 for a complete listing). The basic difference between these and their MATLAB counterpart is that they work on the image as a whole, instead of on a per-column basis. For example, the function sum returns a row vector with the sum over the columns when applied to a numeric matrix, but returns a single number when applied to an image. Besides these, there are some other functions that are only defined for objects of type dip_image. See Section 4.10 to learn about these functions. That section also lists some functions that behave differently than usual when applied to images.

Table 4.4: Dimension manipulation functions.

`cat`	`circshift`	`expanddim`	`flipdim`	`fliplr`	`flipud`
`permute`	`repmat`	`reshape`	`rot90`	`shiftdim`	`squeeze`

4.4 Dimensions

Matlab arrays have at least 2 dimensions. This is not true for an image in a `dip_image` object, which can also have 0 or 1 dimension. That is, for images there is an explicit distinction between a 2D image of size 256 by 1 pixels, and a 1D image of size 256. Even though both images have the same number of pixels and their Matlab array representation is identical, these two images behave differently in many aspects. For example, `size` will return two numbers for the first image, but only one for the second; similarly, it will return an empty array for a 0D image (whereas the corresponding Matlab matrix has a size of `[1,1]`). Use the function `ndims` to obtain the number of dimensions in an image.

The 2D image in this example has a singleton dimension. A singleton dimension is any dimension of size 1. In Matlab arrays, trailing singleton dimensions are removed if the array has more than two dimensions. That is, an array of size 4x1x6x1 is silently converted to an array of size 4x1x6. This never happens with `dip_image` objects.

As in Matlab, operations between two images require that both images have the same number of dimensions, as well as the same size. There is only one exception to this rule: it is possible to do arithmetic operations between two images with different number of trailing singleton dimensions (e.g. between two images with sizes 4x6x1 and 4x6).

Functions used in Matlab to manipulate dimensions have also been overloaded to do the same thing with images. They are listed in Table 4.4. The function `expanddim` listed in this table adds trailing singleton dimensions, and hence does not exist for Matlab arrays.

4.5 Indexing Pixels

In image processing, it is conventional to index images starting at (0,0) in the upper-right corner, and have the first index (usually x), index into the image horizontally. Unfortunately, Matlab is based on matrices, which are indexed starting at one, and indicating the row number first. By encapsulating images in an object, we were allowed to redefine the indexing. We chose not to follow Matlab's default indexing method. This might be confusing at first, and special care must be taken to check the class of a variable before indexing.

`dip_image` objects are indexed from 0 to `end` in each dimension, the first being the horizontal. The `size` function also returns the image width as the first number in the array. Any portion of a `dip_image` object, when extracted, is still a `dip_image` object, and of the same dimensionality, even if it is just a single pixel. Thus, if `a` is a 3D `dip_image` object, `a(0,0,0)` is also a 3D `dip_image` object, even though it only has a single pixel. To get a pixel value as a Matlab array, use `double(a(0,0,0))`. To remove these singleton dimensions use `squeeze`. For example, `a(:,:,2)` is a 3D image with a singleton dimensions, whereas `squeeze(a(:,:,2))` is a 2D image.

Any numeric type can be assigned into a `dip_image` object, without changing the image data

type (that is, the element assigned into the image is converted to the image data type). For example,

```
b(:,0) = 0;
```

sets the top row of the image in `b` to 0. Note that indexing expressions can become as complicated as you like. For example, to sub-sample the image by a factor 3, we could write

```
b = b(1:3:end,1:3:end);
```

Instead of using full indexing (indexing each dimension separately), it is also possible to index using a single (linear) index. Following MATLAB's default behavior, the indices increase in the vertical direction, however they start at 0 for `dip_image` objects ($i = y + x \cdot \text{height}$). The output is always a 1D image.

Finally, it is also possible to index using a mask image. Any binary image (or logical array) can be used as mask, but it must be of the same size as the image into which is being indexed. For example,

```
a(m) = 0;
```

sets all pixels in `a`, where `m` is one, to zero. A very common expression is of the form

```
a(a<0) = 0;
```

(which sets all negative pixels to zero).

Note that the expression `a(m)` above returns a one-dimensional image, with all pixels selected by the mask. It is equivalent to `a(find(m))`, where `find` returns an array of indices where `m` is one. This array is then used as a linear index into `a`.

4.6 Image Arrays

It is possible to join objects of type `dip_image` in an array, and the resulting array is still of type `dip_image`. However, an array of type `dip_image` is treated very differently throughout the interface. To support this idea, the functions `class` and `isa`, when querying an array of type `dip_image`, report that the object is of type `dip_image_array`. The function `isscalar` will only return `true` when the object contains a single (grey-value) image.

To create an array of images use the function `newimar`. It has two forms: in the first form, specifying the array dimensions creates an array of empty images; in the second form, two or more images are joined into an image array. These two examples show both forms:

```
A = newimar(3);      % a 3-by-1 array of empty images
B = newimar(a,b,c);  % a 3-by-1 array with images a, b and c
```

The images in an array do not need to be of the same size or type, since the `dip_image_array` object is just a collection of independent objects of type `dip_image`. Accessing any of those images is possible by indexing through the curly braces ({}). Continuing the example above,

```
c = B{3};
A{1} = a;
```

Note that indexing into the array does follow the standard MATLAB array indexing rules (starting at 1, first index is row number). It is possible to combine both types of indexing,

but only in a fixed order, that is, the curly braces must come before the round braces:[2]

```
A{1}(0,0)
```

Most functions and operations do not work on objects of type `dip_image_array`, but the functions `imarfun` and `iterate` allow operations to be performed on all images in an array. See Section 4.10 for more information on these functions. The functions `size`, `length`, `ndims` and `end` behave differently when their input is an array of images. In this case, they work on the array itself, instead of on the images in it. Instead of using these functions, consider using `imsize` and `imarsize`. The first one always returns the size of the image, even if it is an image array, the second one always returns the size of the image array, even if it is a 1x1 array. `length(a)` can be written as `max(imsize(a))`, and `ndims` can be written as `length(imsize(a))`.

Concatenation of images does not produce an image array, but a larger image. Furthermore, concatenation of image arrays also produces a singe image, where the image arrays are first concatenated to form an image. For example,

```
d = [A];
```

is the same[3] as

```
d = [A{1},A{2},A{3}];
```

If all the images in the array are of the same dimensionality and size, the array can be treated in a special way. We will call such an array a tensor image.

4.7 Tensor Images

A tensor image is a special kind of image array, in which all images are of the same dimensionality and size. If this is the case, `istensor` returns non-zero (true). For these special arrays, some arithmetic operations are defined: `+`, `-`, `*`, `.*` and `./`. They are applied to the arrays in the expected way (that is, tensor by tensor, not image by image).

The pixels of a tensor image can be indexed like a normal image, returning a new tensor image. To get the array at a single pixel, use the `double` function on it. For example, say `A` is a tensor image. Then `A{1}` is an image with the first tensor component as pixel values, `A(0,0)` is a tensor image with a single pixel, and `double(A(0,0))` is a MATLAB array with the tensor values at the first pixel. This indexing is not allowed on image arrays that are not tensors.

Note that the functions `size`, `length`, `ndims` and `end` make no exception for tensor images, and work on the array itself, not on the images in it. Thus, as mentioned in Section 4.6, use the function `imsize` to obtain the size in pixels of a tensor image, and `imarsize` to obtain the size of the tensor at each pixel.

Also note that a scalar image (with one component) is also a tensor image (`istensor` returns true). The function `isscalar` returns true when there is only one tensor component. Additionally, the function `isvector` returns true if the tensor image has more than one component and these are all along one dimension. Relevant similar functions are `iscolumn`, `isrow` and

[2]This is a limitation of the MATLAB parser.

[3]Newer versions of MATLAB simply ignore the brackets when there is only one value inside, so this statement does not hold for all versions of MATLAB.

Table 4.5: Arithmetic functions defined for tensor images.

cross	curl	det	diag	divergence	dot
eig	eig_largest	eye	inner	inv	norm
outer	pinv	rotate	svd	trace	-
+	.*	*	./	.'	'

ismatrix.

Functions defined specifically for tensor images are summarized in Table 4.5. See Section 4.10.

4.8 Color Images

A color image is represented in a dip_image object by a tensor image with some extra information on the color space in which the pixel values are to be interpreted. A color image must have more than one channel, so the tensor image that represents it should have at least two components. Use the colorspace function (see Section 4.10) to add this color space information to a tensor image:

```
C = colorspace(A,'RGB')
```

A color space is any string recognized by the system. Currently defined color spaces are RGB, R'G'B', XYZ, Yxy, L*a*b*, L*u*v*, CMY, CMYK, HCV and HSV. It is possible to specify any other string as color space, but no conversion of pixel values can be made, since the system wouldn't know how. Images with a color space will be displayed by dipshow. If the color space is recognized it will be converted to RGB for display.

To convert an image from one color space to another, use the colorspace function. Converting to a color-space-less tensor image is done by specifying the empty string as a color space. This action only changes the color space information, and does not change any pixel values. Thus, to change from one color space to another without converting the pixel values themselves, change first to a color-space-less tensor image, and then to the final color space.

The function joinchannels combines two or more images into a color image using the specified color space:

```
C = joinchannels('RGB',a,b,c)
```

All operations that are defined for tensor images can be applied to color images. In case a dyadic operation is applied to two color images with different color spaces, no conversion is done. Instead, the color space information is thrown away and both images are treated as tensor images. An operation between a color image and a tensor image produces a color image.

4.9 A Note on the end Method in Indexing

Because of limitations in the MATLAB language, it is impossible to know, for the overloaded end method, if it is being used inside curly or round braces (i.e. whether the last element of the image array is requested, or the last pixel of the image is requested). The solution we have adopted is to suppose image array indexing if the object being indexed is an array, or

pixel indexing otherwise (following the convention used for `size`, `length` and `ndims`). Thus, `end` only works fine inside curly braces if there is more than one image in the object, and it only works fine inside round braces if there is just one image in the object.

Since this is not an optimal solution, we suggest that you use `end` with care. `end` can be substituted with `imsize` or `imarsize` in all cases. These two

```
a{end}, b(end,end)
```

are equivalent to

```
a{prod(imarsize(a))}, b(imsize(b,1)-1,imsize(b,2)-1)
```

4.10 Special Functions

There are some special functions defined only for `dip_image` objects. Many have already been mentioned in preceding sections, but we will summarize them here. We also list some functions that are very different in usage from their MATLAB equivalent.

`cat`

`cat` concatenates images into a larger image, just as the regular `cat` does with arrays. The difference is that it concatenates any image array inputs into a scalar image before joining its inputs. Thus, it always produces a scalar image (see Section 4.6).

`class`

Even though the object is of type `dip_image`, `class` will return `dip_image_array` if there is more than one image in the object. See Section 4.6.

`colorspace`

This function will add and retrieve color space information from a tensor image with two or more components. It can also be used to change the color space of a color image, in which case the pixel values will be recomputed. See Section 4.8 for more information on color spaces.

`convhull`

This overloaded function works differently from the MATLAB one. The output is a binary image containing the solid convex hull of the binary image input. `convhull(a,'no')` returns only the outer shell, (i.e. the volume is not filled in).

`curl, divergence`

`curl` calculates the rotation of a 2D or 3D vector image. `divergence` computes the divergence of a vector image. Both methods have different input arguments from their base counterparts.

`datatype`

`datatype` extracts the data-type string from a `dip_image` object. If the input is an image array, it expects as many output parameters as images are in the array. The string returned is a DIP*lib* data type name, not a MATLAB class name (i.e. `'sfloat'`, not `'single'`); see Table 4.1. To change the data type of an image, use the function `dip_image`.

dip_array

dip_array extracts the data array from a dip_image object. If the input is an image array, it expects as many output parameters as images are in the array, and puts one array into each. Alternatively, if only one output parameter is given, and the input is a tensor image, the tensor components are catenated along a new dimension before the data array is extracted. The data array is returned as-is unless a second input argument is used to specify a data type.

double, single

These functions convert a scalar image (in an object of type dip_image) to a MATLAB array of type double (MATLAB's default data type) or type single (single precision floating point). They are equivalent to calling dip_array with 'double' or 'single' as a second argument.

Also defined are uint8, uint16, uint32, int8, int16, int32 and logical.

eig

As opposed to the builtin eig function, this version only works on 2x2 or 3x3 symmetric tensor images, such as the structure tensor, the Hessian, etc.

eig_largest

This function computes the largest eigenvector for a square tensor image using the Power method. An optional second output argument contains the corresponding eigenvalue.

The second argument in the call

```
v = eig_largest(a,sigma)
```

specifies the tensor smoothing that should be applied before calculating the eigenvector.

expanddim

expanddim(a,n) increases the dimensionality of the image a to n, by appending dimensions of size 1.

find, findcoord

find works similarily to the base version, except it is not possible to obtain [I,J] indices as output. The indices returned are always linear indices. An optional second output argument receives the non-zero values. To obtain the coordinates of non-zero values, use findcoord instead. It returns the coordinates of the pixels with non-zero values as a single array, with as many columns as dimensions in the input image, and one row for every non-zero pixel. Note that this matrix cannot be used directly to index an image.

gradient

The overloaded version of gradient returns a vector image, instead of multiple outputs. The derivatives are computed using Gaussian derivatives.

imarfun

Table 4.6: Options for imarfun in its first form. These operations compute a single value for each image in the array.

Option	Meaning
`'isempty'`	true for empty image
`'islogical'`	true for binary image
`'isreal'`	true for non-complex image
`'ndims'`	number of dimensions of image
`'prodofsize'`	number of pixels in image
`'max'`	maximum pixel value in image
`'mean'`	mean pixel value in image
`'median'`	median pixel value in image
`'min'`	minimum pixel value in image
`'std'`	standard deviation of pixels in image
`'sum'`	sum of pixels in image

Table 4.7: Options for imarfun in its second form. These operations combine all images into a new image.

Option	Meaning
`'imsum'`	sum of all images
`'improd'`	product of all images
`'imor'`	true if any pixel is non-zero
`'imand'`	true if all pixels are non-zero
`'immax'`	maximum pixels over all images
`'immin'`	minimum pixels over all images
`'imeq'`	true if pixel is equal in all images
`'imlargest'`	index of first image with largest pixel value
`'imsmallest'`	index of first image with smallest pixel value

`imarfun` applies some other function on an array of images. It has two modes.

In the first mode, it produces a numeric array with the same size as the input image array, where each number is some measure for each image. The possibilities are listed in Table 4.6. This example replaces the image `a` for the empty images in the array `A`:

```
I = imarfun('isempty',A);
A{find(I)} = a;
```

The second mode applies an operation to all images in the array, which must all be of the same size (`istensor` returns true), producing an image with the same size as the images in the array. The possibilities are listed in Table 4.7. For example, to get the sum of all images in the array `A`, we can do either of these:

```
res = imarfun('imsum',A);
res = A{1}+A{2}+A{3}+...+A{end};
```

`imarsize, imsize, size`

The function `size` works differently if the input is of type `dip_image` or `dip_image_array`. To solve the problems that yields, use the functions `imsize` to obtain the size of an image (including tensor images or color images), and `imarsize` to obtain the size of an image array, even if the image is scalar.

`imarsize`, just like the function `size` when applied to an array, always returns at least two values. `imsize`, on the other hand, can return fewer values.

`ind2sub, sub2ind`

These functions have the same function as their base counterparts, but instead of using subscripts specified with one array for each dimension, they take and return a single coordinate array, compatible to that returned by `findcoord`. Also, instead of a size array, they take an image as input.

`inner, outer`

These calculate the inner and outer product of two tensor images. `outer` is only defined for tensors with three components. They are equivalent to `dot` and `cross`, respectively.

`isa`

`isa(a,'dip_image')` returns true only if there's a single image in the object `a`. `isa(a,'dip_image_array')` returns true only if there's multiple images in the object. `isa(a,class(a))` always returns true. See Section 4.6.

`iscolor`

`iscolor` returns true if the input image is a tensor image and contains color space information (see Section 4.8).

`isscalar`

`isscalar` returns true if the argument is a `dip_image`, not a `dip_image_array`. That is, the argument is a single, scalar, grey-value image.

`istensor`

`istensor` returns true if all images in an image array are of the same size. A tensor image is treated differently than a regular image array (see Section 4.7). Note that a scalar image is also a tensor image.

Also defined are `iscolumn`, `ismatrix`, `isrow` and `isvector`. These give additional information about the shape of the tensor.

`iterate`

`iterate` loops through each image in the image arrays it gets as input, and calls the function fun with the given parameters. This is a very versatile function, and allows a combination of image arrays, single images and other objects as input. The only requirement is that all the image arrays are of the same size.

For example, let `A` and `B` be N-by-M `dip_image_array` objects. Then

```
C = iterate('max',A,B);
```

is the same as

```
C = newimar(N,M);
for ii=1:N*M, C{ii} = max(A{ii},B{ii}); end
```

Use the function `iterate` to apply filters to color images.

`length, ndims`

The functions `length` and `ndims`, much like `size`, work differently on scalar images and on tensor or color images. If the image is scalar, they work on the image itself, meaning that `ndims` returns the dimensionality of the image and `length` returns the maximum size of the image. However, if the input is a tensor or a color image, which are implemented as image arrays, these functions work on the array rather than the image. So now `ndims` returns the dimensionality of the tensor (or just 2 for normal color images), and `length` returns the maximum tensor size (or the number of channels in the color image).

`max, min`

These functions have two different forms.

In the first form, they return the global maximum/minimum in the image and, optionally, its position:

```
[value,pos] = max(a,m);
```

The second input argument is a mask image, for ROI processing (this must be a binary image or logical array). It is also possible to process only a specified set of dimensions. For example, assuming `a` is 3D, this command returns a 3D image with two singleton dimensions, where each pixel `i` contains the maximum over `a(i,:,:)`:

```
value = max(a,m,[2,3]);
```

If no mask is required, pass `[]` for the mask argument. A second output argument gives the location of the maximum, but only can be given if the projection is along one dimension:

```
[value,pos] = max(a,m,1);
```

Here, `a(pos(0,i,j),i,j) == value(0,i,j)`.

The second form takes two images and returns an image with the supremum of the two:

```
c = max(a,b);
```

`mean, std, var`

These return the mean intensity, standard deviation or variance of the pixel values in an image. It is possible to add a mask:

```
value = mean(a,m);
```

As in `max`, it is possible to specify a set of dimensions that are to be processed:

```
value = mean(a,m,[2,3]);
```

If no mask is required, pass `[]` for the mask argument.

`median, percentile`

`percentile` returns the `p` percentile of all pixels in the image `a`, and, optionally, its position:

```
[value,pos] = percentile(a,p);
```

Note that `percentile(b,50)` is exactly the same as `median(b)`, `percentile(b,0)` is a silly way of computing `min(b)`, and `percentile(b,100)` is a silly way of performing `max(b)`.

Like `max` and `min`, these two function also allow specifying a set of dimensions that are to be processed, and a mask image `m` for ROI processing:

```
[value,pos] = median (a,m,[2,3]);
[value,pos] = percentile (a,p,m,[2,3]);
```

`numel`

The function `numel` always returns 1. To obtain the number of pixels in an image, use `prod(imsize(a))`. To obtain the number of tensor elements, or the number of images in an image array, use `prod(imarsize(a))`.

`phase`

`phase` is defined the same as `angle`, and is provided because it might be easier to remember for some users. It returns the angle of the complex values in an image.

`pow10`

This function was added just to complete MATLAB's collection of `pow2`, `log2`, and `log10`.

`prod, sum`

These methods return the product or sum of all pixel values in an image. Arguments are identical to `mean` and the like.

`rot90`

This function works on images of any dimensionality, not only 2D images. However, the rotations always occur in the x-y plane.

`rotate`

The overloaded method `rotate` has nothing to with MATLAB's `rotate`. Applied to a 3D-vector image, it rotates the vectors around an axis given by a second vector image or vector.

4.11 Review of the Differences Between a dip_image and a MATLAB Array

As we have seen, objects of type `dip_image` have some differences with respect of regular MATLAB arrays. The main difference is in indexing. We start counting pixels from 0, and the first index counts from left to right. This ordering is also used by functions such as `size`, in which the first number is the image width and the second one the height. Finally, `ndims` can return 0 or 1, which it never does for MATLAB arrays, and `size` can return an empty array

or a scalar, which it never does for Matlab arrays. The reason is that zero-dimensional and one-dimensional images are allowed, and are not seen as a special case of two-dimensional images. Furthermore, singleton dimensions at the end are not ignored.

When a Matlab command results in an object of type `dip_image`, and it is not ended with a semicolon, the image is displayed to a figure window, instead of having its pixel values shown in the command window. This is the default behavior, but can be overridden.

There are no array operators for scalar images, all operators work on a pixel-by-pixel basis. All functions that work on the columns of numeric arrays work on the image as a whole when applied to a `dip_image` object.

A collection of images can be stored in an object of type `dip_image`. For the purposes of the toolbox, such an object is called a `dip_image_array`. Syntax for indexing into such a collection is similar to that used to index into a `cell` array (which is a collection of any type of arrays), but should not be confused for one. A special type of image array is used as a tensor image, for which a whole range of functions is available. Color images are tensor images with color space information.

Objects of type `dip_image` cannot be used in functions of the MathWorks' Image Processing Toolbox. Although most of Matlab's functions work on `dip_image` objects, not every function will work as expected. Use the functions `dip_array`, `double` or `uint8` to convert the image to a format recognizable by these functions.

Chapter 5

The dip_measurement Object

The function measure (and the low-level dip_measure function in DIP*lib*) returns the measurement results in an object of type dip_measurement. It contains all the measures done on an image in a manageable way.

5.1 Extracting Measurement Data

The data in the dip_measurement object can be accessed in a very simple way, but only for reading, not writing (i.e. data manipulation is not allowed).

Indexing with parentheses is used to access the measurements belonging to one or more objects. The index used must match the label ID of the object in the image, and the returned value is an object of type dip_measurement.

The dot operator is used to extract the values corresponding to a single measurement. The array returned is of type double.

For example,

```
msr(11:15).size
```

will return a double array with five elements, being the sizes for objects number 11 through 15. Note that element 11 doesn't need to be placed 11th in the list of measurements. If only objects starting at 10 were measured, the above example is equivalent to

```
msr.size(2:6)
```

since msr.size returns a double array, whose second element would be the size of object number 11.

The end method will return the last label ID in the object. double converts the data in the object to a double array, loosing the names of the measurements and the label IDs.

5.2 Other Information on the dip_measurement Object

Besides extracting the measured data, you might want to gain more knowledge on the object you are dealing with (e.g. which measurements were taken and how many of them are there). This section describes functions used for this purpose.

fieldnames returns the names of the measurements present in the object.

isempty returns true if there is no data in the object.

size returns the number of IDs as the first dimension, and the number of measurements as the second. Note that the number of measurements returned by size does not need to be

equal to the number of names returned by `fieldnames`. If a measurement contains more than one value for each object, each of these is taken as a measurement. Thus, the number of measurements is the number of scalar values assigned to each object. `size(double(msr))` returns the same value as `size(msr)`.

5.3 Combining Measurement Data

To join measurements produced by different calls to measure, use the default MATLAB syntax. However, there is the limitation that, when joining measurements, they must contain either the same measurements on different objects, or different measurements on the same objects. Horizontal and vertical catenations produce different effects.

`[A,B]` joins two measurement objects with the same label IDs, but different measurements. If some measurements are repeated, or if the label IDs don't match, an error is generated.

`[A;B]` joins two measurement objects with the same measurements, on different label IDs. If some IDs are repeated, or if the measurements don't match, an error is generated.

In some cases, objects in different images have the same labels. These need to be changed before catenation is possible. This is done by the following syntax:

```
msr.id = 51:73;
```

The length of the array assigned to the IDs must have the same number of elements as the measurement object.

Similarly, it is possible to measure the same thing on different images of the same objects. For example, one might measure the average grey value on all three channels of an RGB image. To join these measurements into a single object, it is possible to add a prefix to the names of the measurements:

```
msr1.prefix = 'red_';
msr2.prefix = 'green_';
msr3.prefix = 'blue_';
msr = [msr1,msr2,msr3];
```

Note that this prefix cannot be changed, only added to. For example,

```
msr.prefix = 'A';
msr.prefix = 'B';
```

causes the measurements in `msr` to have names like `'BAsize'`.

5.4 Adding Measurement Data

Furthermore, it is possible to add your own measurements to a `dip_measurement` object:

```
msr.temperature = mydata;
```

You can name them whatever you want, except `id` or `prefix`, since that would invoke the syntaxes explained previously. The array `mydata` in the example above has to be an array with the same number of columns as there are labels in the `dip_measurement` object.

The function `rmfield` deletes a measurement from the object.

5.5 Converting a dip_measurement Object to a dataset Object

The `dip_measurement` object provides an overloaded version of the `dataset` function, which will convert the measurement object into a PRTOOLS data set. An optional second argument allows giving each object a class ID:

```
ds = dataset(msr,[1,1,1,2,2,3,2,3,3,2,1])
```

For more information on the PRTOOLS pattern recognition toolbox, go to http://www.prtools.org/.

5.6 Creating a dip_measurement Object with Your Own Data

To create an object of type `dip_measurement`, use the `dip_measurement` function. Its syntax is:

```
msr = dip_measurement(id,'msrname1',msr1,'msrname2',msr2,...)
```

where `id` is a vector containing the object IDs, and `'msrname1'` and `msr1` are the name and results of a measurement. The number of columns in `msr1` should match the number of elements in `id`, as each of the columns represents the result of a measurement on a single object.

If the name of a measurement is not given, `'dataX'` is assumed, where the 'X' is the ordinal number of the measurement. If `id` is not given, `1:N` is assumed. Note that the only way it is possible to recognize whether `id` is missing is if the first argument is a string.

5.7 Backwards Compatibility

The `dip_measurement` object is new to version 1.1 of the toolbox. However, it has been implemented in such a way that most old code doesn't break. The structure that used to be returned by measure in earlier versions can still be obtained with the struct function:

```
oldmsr = struct(msr);
```

The `dip_measurement` constructor can be used to convert this structure back to an object. Converting to a structure is the only way of manipulating the measurement data.

Chapter 6

Figure Windows

The display is a very important part of any image-processing package. `dip_image` objects containing scalar or color images with 1 to 4 dimensions are displayed to MATLAB's figure windows. These windows are completely cleared beforehand, meaning that images never share a window with each other or with other graphical elements. This chapter describes the possible interactions with figure windows, how to link variables with them, and their placing on the desktop.

6.1 The Figure Window Menus

The display for an image contains four menus: "File", "Sizes", "Mappings" and "Actions".

The first menu contains a "Save display..." option that saves the display to a TIFF file. This allows you, for example, to save an image with labels, or to zoom into a portion of an image and only save that. It also contains a "Close" and a "Clear" item. On Windows machines, there is a "Copy display" option. It does the same as "Save", but writes the image as a bitmap to the clipboard, so that it can be pasted into other applications.

"Sizes" contains options that call `diptruesize`, which causes the image to be displayed with an aspect ratio of 1, and various different zoom factors (see Section 7.4). It also contains an option that causes a the image to be stretched to fill the figure window. The last option on this menu, "Default window size" resizes the window to some pre-defined size (which is 256 by 256 pixels, but you can change it using `dipsetpref`, see Sections 7.8 and 8.4).

"Mappings" contains different ways of mapping the data for display. These options correspond to calls to `dipmapping` (see Section 7.3). The first section here contains stretching modes, which correspond to the range parameter in `dipshow` (see Section 7.2); one of these options is "Manual...", which, through a dialog box, allows the user to select a custom range. The second section, only available for grey-value images, selects a colormap. The options in the first section will sometimes change the selection of the colormap. If the image being displayed is complex, this menu allows choosing the complex to real mapping performed (magnitude, phase, real or imaginary part). For 3D and 4D images you can select the orientation of the slices shown (X-Y, X-Z, Y-Z, X-T, Y-T, Z-T), as well as decide whether the stretching mode selected is to be computed on the whole volume ("Global stretch") or only on the current slice.

The "Actions" menu selects the actions that can be performed through the mouse. The options "none", "Pixel testing", "Zoom", "Looking glass" and "Pan" (which correspond to the `diptest`, `dipzoom`, `diplooking` and `dippan` commands) are available to all image types. The 3D/4D image display also contains an option to "Step through slices" (`dipstep`), and

the 2D grey-value image display contains an option for “Orientation testing” (`diporien`). See Section 6.2 for more information on these modes, and Section 7.5 for the associated commands. This menu also contains a command to enable or disable the keyboard functionality in the window. See Section 6.3 for more information on this.

Finally, the “Actions” menu on 3D/4D images contains some more options:

- “Link displays” (`diplink`, see Section 7.6) allows the user to link a display with other displays. When stepping through the slices of this image, or changing the orientation of the slicing, the images in the other displays will be kept in sync. This can be used to easily compare various 3D or 4D images.
- “Animate” (`dipanimate`) will step through all slices in sequence. Calling this function from the command line allows the user to choose the speed of this animation.
- “Max projection” and “Sum projection” (`dipprojection`) open a new window with the chosen type of projection, along the current visualization axis.
- “Isosurface plot” (`dipisosurface`) also opens a new window, showing an isosurface plot of the image. This window contains some controls to modify the surface. You should be aware that it takes a while to generate an isosurface. It is recommended to smooth and down-sample an image before generating an isosurface plot. The isosurface plot is only available for 3D displays.

6.2 Using the Mouse in Figure Windows

As discussed above, the “Actions” menu allows selecting a mode for the mouse to work in. Depending on the dimensionality and type of the image, the modes are (the commands between brackets can also be used to turn these modes on and off, see Section 7.5):

- “None”: The mouse does nothing.
- “Pixel testing” (`diptest`): The mouse is used to examine pixel values and location.
- “Orientation testing” (`diporien`): The mouse is used to examine local orientation.
- “Zoom” (`dipzoom`): The mouse is used to zoom the image in and out.
- “Looking glass” (`diplooking`): The mouse is used to enlarge a part of the image.
- “Pan” (`dippan`): The mouse is used to pan the image if it doesn’t fit in the window.
- “Step through slices” (`dipstep`): The mouse is used to step through the slices of a 3D or 4D volume.

When `diptest` is turned on, depressing the left mouse button will cause the current cursor position to be displayed in the title bar, together with the grey-value (or color values) of the pixel at that location. It is possible to move the mouse while holding down the button. Depressing the right mouse button does the same thing, but the cursor position becomes the origin of the coordinate system. This mode allows for length measurements in images.

When `diporien` is first turned on, a dialog box asks for the orientation image to associate to the currently displayed image. This dialog can also calculate that image for you, using the function `structuretensor`. Depressing any mouse button over the image now converts the cursor into a line, aligned with the local image orientation. Like in `diptest`, the title

bar changes to display the coordinates and local orientation. The only way of changing the orientation image associated with this display is to set "Actions" to "None", and then enable `diporien` again. Displaying a new image in this display also removes the orientation image.

When `dipzoom` is turned on, the mouse can be used to zoom the image in and out:

- Clicking with the left mouse button zooms the image in (with a factor 2).
- Clicking with the right one will zoom the image out (with a factor 2).
- Double-clicking any mouse button will cause the image to be stretched to fill the figure window.
- Dragging a rectangle around an area of interest will cause it to be zoomed-in on.

The aspect ratio is set to 1:1 when zooming in or out, except after double-clicking. See Section 6.3 to learn how to zoom using the keyboard.

`dippan` enables the user to use the mouse to pan (move) the image if it is larger than the window. Just press the left mouse button and move the mouse with the button down. It is also possible to pan using the keyboard (see Section 6.3).

When `dipstep` is selected, it allows the user to click or drag the cursor over the image to go back and fourth through the slices that make up the volume. Moving the mouse down or to the right, while holding down the left button, displays higher slice numbers along the first hidden dimension. Moving the mouse up or to the left displays lower slice numbers. Alternatively, click with the left mouse button to go up, and with the right one to go down. If the displayed image is 4D, dragging the mouse with the right button down moves the display along the second hidden dimension. Section 6.3 explains how to do step through slices with the keyboard.

6.3 Using the Keyboard in Figure Windows

When the keyboard is enabled for a display window, it can be used to step through the slices of a 3D/4D image, zoom in and out, and pan the image. These functions are independent of the chosen mode for the mouse under the "Actions" menu.

The keys **N** and **P** step to the next and previous slice, respectively, of a 3D image. Additionally, you can type the number of a slice and press **Enter** to go to it. Note that slice numbers start with 0. In case of a 4D image, **N** and **P** step through the first hidden dimension (Z), whereas **F** and **B** step through the second hidden dimension (T).

The keys **I** and **O** are used to zoom in and out, respectively. The zoom factor is 2. When zoomed in, use the following keys to pan the image and get to the area of interest: **W** for up, **S** for down, **A** for left, and **D** for right. With MATLAB 6 and newer, it is also possible to use the arrow keys.

The **Esc** key disables the keyboard. This is useful under Windows, where displaying an image causes its window to gain keyboard focus. You would have to click on the command window to continue typing a new command. Instead, press **Esc**, which disables the keyboard for the window and causes your keystrokes to be send to the command window. To enable the keyboard again, use the menu item "Enable keyboard" under the "Actions" menu. With the

command

```
dipsetpref('EnableKeyboard','off')
```

you disable the keyboard by default, and will have to use the above mentioned menu item to enable it. See Sections 7.8 and 8.4.

6.4 Linking Variables with Figure Windows

A variable name can be linked with the handle of a figure window, such that any image stored in that variable will always be displayed in the same window. This is done through the `dipfig` function (see Section 7.7). It is not possible to link a single variable with more than one figure window, but it is possible to link many variables to the same figure window. This system allows the user to create a series of figure windows that will be reused, instead of having new windows created all the time. These links do not, however, promise that an image displayed is actually up-to-date. Changing the contents of a variable does not change the contents of a figure window. By not adding the semicolon at the end of commands, it is possible to automatically update the figure windows (see Section 4.2).

A special name `'other'` is defined in `dipfig`, that is a substitute for all variables not explicitly linked to a figure window. It allows the user to have a window for all possible images he can create. `'other'` can be linked to a series of windows, which then will be used sequentially.

Closing a window does not destroy the links that were made for it. Since variable names are linked to window handles, a window can be reopened to display the image with which it is linked.

Note that many toolbox functions that require a figure window handle as input also accept a variable name. Variable names linked with a figure window are considered aliases for a figure window handle.

6.5 Setting the Position of Figure Windows

The position of a figure window can be changed by manipulating its `'Position'` property, which is defined by an array with four values: `left`, `bottom`, `width` and `height`.

```
set(handle,'Position',[left,bottom,width,heigth]);
```

The coordinates for figure windows start at the bottom-left corner of the screen, and are in screen pixels by default. This can be changed to `centimeters`, `inches` and other units:

```
set(handle,'Units','points');
```

See "MATLAB Function Reference" for more information on figure window properties.

The `dipfig` function has an additional optional parameter, which can be used to set the position of a figure window at the same time that it is created. This parameter comes at the end of the parameter list, and is the same array used for the `'Position'` property:

```
dipfig('a',[400,600,256,256]);
```

The `width` and `height` values are those of the image that will fit in the window, and the window itself is drawn around this area. These values are always in screen pixels.

If an image is larger or smaller than the size of the window, the window will be resized so that the image fits exactly. That is, unless the `'TrueSize'` option is turned off (see Section 8.4), in which case the window will not be resized, and the image will be stretched to fit. To have your windows fixed on the desktop, disable the `'TrueSize'` option.

As with all other settings, the position of the figure windows cannot be saved from one session to the next. Add the appropriate commands to your `startup.m` or `dipinit.m` files to have the same settings across sessions (see Section 8.3).

Chapter 7

Toolbox Functions

7.1 The GUI: dipimage

The GUI is started with the `dipimage` command. It contains menus with all available image-processing functions in the toolbox. After choosing any of these menu items, the GUI window transforms itself into a dialog box so that you can enter the appropriate parameters. The controls that allow entering images have a context-menu (obtained by right-clicking in them) with the names of the images currently defined. It is possible to enter the name of a variable containing an image or any valid MATLAB statement that evaluates to image data. (The same is true for other objects, like measurements or data-sets. Also, the window selection control, which is a drop-down list, can be updated through its context-menu.) Pressing the "Execute" button causes the function to be called. There is also a button to get help on the particular function. The whole process is rather obvious and self-explanatory, and no further words shall be wasted on it.

In the "File I/O" menu is an option "Record macro". When selected, the user is asked for the name under which the macro will be recorded. The extension will be ".m", indicating it is an M-file. MATLAB scripts are M-files, and can be executed by typing their name on the command line. After entering the name (let's assume we use the default "macro.m"), this file will be created (or appended to if it already exists[1]), and loaded in the editor. Any commands executed through the DIP*image* GUI will be written to this file, in the same manner as that they are echoed to the command line. When finished, select the same menu item again (its text will have changed to "Stop recording macro"). Typing the macro name on the command line:

```
macro
```

will execute all recorded commands again. It is possible to append commands to a recorded macro by starting the recording again with the same macro name. It is also possible to edit the macro in the editor. However, if you edit the macro file while recording, do remember to save your changes before executing another command through the GUI.

7.2 The dipshow Function

`dipshow` shows a `dip_image` object, as an image, in a figure window (that is, as long as it is a binary, grey-value or color image, and has between 1 and 4 dimensions). An optional second argument indicates the display range required, and allows more flexibility than the options

[1] MATLAB will ask if it is OK to overwrite the file, don't worry, the file will not be overwritten but appended to.

in the “Display” menu. The general form for `dipshow` is:

```
dipshow(a,range,colmap)
```

where `range` is either a grey-value range that should be displayed, or one of `'log'` or `'base'`. A range is a numeric array with two values: a lower and an upper limit. The pixels with the same or a lower value than the lower limit will be mapped to black. The pixels that are equal or larger than the upper limit will be mapped to white. All other values are linearly spaced in between. The strings `'lin'` and `'all'` and the empty array are a shortcut for `[min(image),max(image)]`, and cause the image to be stretched linearly. The string `'percentile'` is a shortcut for `[percentile(image,5) percentile(image,95)]`, and `'angle'` and `'orientation'` are equivalent to `[-pi,pi]` and `[-pi,pi]/2` respectively. The default range is `[0,255]`, which is used unless a range is given explicitly. `colmap` is a colormap. It can either be `'grey'`, `'periodic'`, `'labels'` or an array with 3 columns such as those returned by the MATLAB functions `hsv`, `cool`, `summer`, etc. (see the help on `colormap` for more information on this).

The strings `'angle'` and `'orientation'` imply `'periodic'` if no explicit colormap is given. This colormap maps both the maximum and minimum value to the same color, so as to hide a jump in angle or orientation fields. The string `'labels'` implies a range of `[0,255]`, and produces a colormap that gives each integer value a distinct color.

The string `'log'` causes the image to be stretched logarithmically. `'base'` is a linear stretch that fixes the value 0 to a 50% grey value.

Examples:

```
dipshow(a,'lin',summer(256))
dipshow(a,[0,180],'periodic')
```

If the input argument is a color image, it will be converted to RGB for display.

The image is displayed in a figure window according to the name of the variable that contains the image. Links can be made using the `dipfig` function (see Section 7.7). If the variable name is not registered, a new figure window is opened for the image. To overrule this behavior, it is possible to specify a figure handle in the parameter list of `dipshow`:

```
dipshow(handle,image,'lin')
```

Finally, an optional argument allows you to overrule the default setting for the `'TrueSize'` option. By adding the string `'truesize'` at the end of the parameter list for `dipshow`, you can make sure that `diptruesize` is actually called. The string `'notruesize'` does the reverse.

See Chapter 6 for more information on the figure windows used by `dipshow`.

7.3 Figure Window Support: `dipmapping`

The function `dipmapping` can be used to change the image-to-display mapping. All menu items under the “Mappings” menu are equivalent to a call to `dipmapping`. In a single command, you can combine one setting for each of the four categories: range, colormap, complex-to-real mapping, the slicing direction and the global stretching for 3D images.

```
dipmapping(h,range,colmap,torealstr,slicingstr,globalstr)
```

changes the mapping settings for the image in the figure window with handle `h`. It is not

necessary to provide all four values, and their order is irrelevant. `range` can be any value as described for `dipshow` in Section 7.2: a two-value numeric array or a string. `colmap` can contain any of the strings described for `dipshow`, but not a colormap. To specify a custom colormap, use `dipmapping(h,'colormap',summer(256))`. `torealstr` can be one of: `'abs'`, `'real'`, `'imag'` or `'phase'`. `slicingstr` can be one of: `'xy'`, `'xz'`, `'yz'`, `'xt'`, `'yt'` or `'zt'`. `globalstr` can be one of `'global'` or `'nonglobal'`. If you don't specify a figure handle, the current figure will be used.

Additionally, you can specify a slice number. This is accomplished by adding two parameters: the string `'slice'`, and the slice number. These must be together and in that order, but otherwise can be combined in any way with any of the other parameters. The same is true for the `'colormap'` parameter.

7.4 Figure Window Support: diptruesize

The "Sizes" menu contains some options to call `diptruesize` (see Section 6.1). This function causes an image to be displayed with an aspect ratio of 1:1, each pixel occupying one screen pixel. An argument gives the zoom factor. For example, `200` would make the image twice as large on the screen, but with the 1-to-1 aspect ratio:

```
diptruesize(200)
```

`diptruesize('off')` causes the image to fill the figure window, possibly loosing the aspect ratio. `diptruesize` accepts a figure handle as an optional first argument. If you provide a handle, you must also provide a zoom factor.

7.5 Figure Window Support: diptest, dipzoom, *et al.*

As explained in Section 6.1, the first section of items under the "Actions" menu correspond to the `diptest`, `diporien`, `dipzoom`, `diplooking`, `dippan` and `dipstep` commands. We explain here how to use the functions. The modes they activate are described in the section previously referred to.

All five functions have the same syntax:

```
diptest on
```

enables the mode, and

```
diptest off
```

turns it off. `diptest`, by itself, toggles the state. The current window is the last one activated. You can select a window either through some mouse action on that window, or by typing in the MATLAB command window:

```
figure(handle)
```

where handle is the handle of the figure window, which should be visible on the title bar. If you know this handle, you can also directly use it as a parameter to `diptest`:

```
diptest(handle)
```

or

```
diptest(handle,'on')
```

7.6 Figure Window Support: `diplink`

`diplink` is the command that corresponds to the "Link displays..." menu option for 3D/4D images (see Section 6.1). It is used in much the same way as the functions in Section 7.5. When turning on, it displays a dialog box that allows the user to select the windows with which to link. Alternatively, it is possible to specify the figure windows with which to link through the command line:

```
diplink('a',{'b','c','d'})
```

or

```
diplink(1,[2,10,6])
```

7.7 Creating, Linking and Clearing Figure Windows: `dipfig` and `dipclf`

The single most important thing that can be customized in the DIP*image* environment is the way that images are displayed to figure windows. It is possible to link a variable name with a figure handle, such that that variable is always displayed in that same window. If a variable is not linked to any window, a new one will be opened to display it. The command

```
dipfig a
```

opens a new figure window and links it to the variable named `a`. Whenever that variable (if it contains an image) is displayed, it will be send to that window. If the window is closed, it will be opened again to display the variable. It is possible to link more than one variable to the same window, like in the next example (which uses the functional form):

```
h = dipfig('a')
dipfig(h,'b')
```

Finally, there is a special variable name, `'other'`, that creates a link for all variables not explicitly linked to a window. It is possible to have many windows linked to this special name, and they will be used alternately. Creating a window for `'other'` avoids the opening of new windows for 'unregistered' variables.

To remove the links, type

```
dipfig -unlink
```

Unlinking only a specific variable is not implemented.

To clear all figure windows (for example at the beginning of a demo), use the function `dipclf`. It doesn't change the position or size of any window, but removes the images in them. `dipclf` can also be used to clear selected windows by giving it an array with handles or a `cell` array with names as an argument (in a `cell` array you can actually combine numeric handles and variable names).

7.8 Toolbox Preferences: `dipsetpref` and `dipgetpref`

All toolbox preferences are stored in memory, and are only accessible through the `dipsetpref` and `dipgetpref` functions. They are listed in Section 8.4.

```
v = dipgetpref('name');
```

retrieves the value of the named preference. Two special forms print all current preferences and all factory settings to the command window:

```
dipgetpref
dipgetpref factory
```

Setting a preference is similar:

```
dipsetpref('name',value)
```

Furthermore, it is possible to set many preferences at once:

```
dipsetpref('name1',value1,'name2',value2,'name3',value3,...)
```

7.9 Interactive Tools: `dipcrop`, `dipgetcoords`, *et al.*

These are some tools that, using an image display, allow the user to select points or regions in an image. `dipgetcoords` returns the coordinates of one or more points selected by clicking on an image. `dipcrop` returns a rectangular portion of an image selected by dragging a rectangle. `dipprofile` returns a 1D image interpolated along a path selected by the user on the display. `diproi` returns a mask image (ROI stands for region of interest) created by selecting the vertices of a polygon; it can only be used with 2D images.

`dipgetimage` retrieves the image from a display. Use it if you lost an image but can still see it in its display.

`dipstackinspect` lets the user click on a 3D display, and shows a 1D plot of the hidden dimension at that point. The tool will stay active until the right mouse button is clicked over the image.

7.10 Other 3D Visualization Tools: `dipanimate`, `dipisosurface`, `dipprojection`

These functions handle the callback for some visualization tools available on 3D or 4D displays. `dipanimate` automatically steps through slices. Optional input arguments allow to set the speed and whether to loop indefinitely or not. `dipisosurface` shows a 3D rendering of an isosurface of a 3D image. `dipprojection` calculates and displays various types of projections.

7.11 Image Processing Functions

The largest part of the toolbox is made out of the image processing functions. Most of them are listed in the menu system of the GUI, and all are listed by typing

```
help dipimage
```

The usage of each function can be retrieved through the `help` command or through the GUI.

```
function out = func_name(varargin)
% The next line defines the parameters your function requires
paramlist = struct(...);
% The next section causes this function to be integrated in
% the menu system
if nargin == 1
   s = varargin{1};
   if ischar(s) & strcmp(s,'DIP_GetParamList')
      out = paramlist;
      return
   end
end
% Below, add your own code
out = process_image(varargin);
```

Figure 7.1: Skeleton GUI function.

7.12 Adding Functions to the GUI

To add a function to the GUI, it must:

- respond in certain ways to certain inputs, so that the GUI can query it for parameters, and
- be on both the MATLAB path and the DIP*image* path.

The second requirement is the easiest. If you have your functions in a directory called `/myhome/mytools/`, then this command accomplishes it:

```
dipaddpath('/myhome/mytools')
```

The first requirement is a bit more complicated. To add this functionality to your own function, copy the code in Figure 7.1. It shows a complete skeleton for a function. The line that is not written-out is the one that assigns a structure into `paramlist`. This structure is the most complicated part of the function (Figure 7.2 shows an example), but allows both the automatic parsing of the input parameters and the drawing of the dialog box in the GUI. Automatic parameter parsing is discussed in Section 7.13.

The parameter structure `paramlist` contains four values:

`menu`	Name of the menu to place the function in (string).
`display`	Name for the function in the menu (string).
`inparams`	Structure array with input parameters.
`outparams`	Structure array with output parameters.

The function will be added to the end of the menu specified (in alphabetical order). If you want to change the order of the menu items, you will need to create a `localdipmenus` function (see Section 8.2).

```
inparams = struct(...
    'name',{'image_in','percentile','filterSize','filterShape'},...
    'description',{'Input image','Percentile','Size of filter',...
                   'Shape of filter'},...
    'type',{'image','array','array','option'},...
    'dim_check',{[],0,1,0},...
    'range_check',{'scalar',[0,100],'N+',{'rectangular', 'elliptic',...
                   'diamond','parabolic'}},...
    'required',{1,0,0,0},...
    'default',{'ans',50,7,'elliptic'}...
);
outparams = struct(...
    'name',{'image_out'},...
    'description',{'Output image'},...
    'type',{'image'},...
    'suppress',{0}...
);
paramlist = struct(...
    'menu','Filters',...
    'display','Percentile Filter',...
    'inparams',inparams,...
    'outparams',outparams...
);
```

Figure 7.2: Sample parameter structure (belongs to the function `percf`).

`paramlist.inparams` defines the input parameters, and contains the following fields for each parameter:

`name`	Variable's name (string). Not used (for now).
`description`	Description to show the user (string).
`type`	Expected data type (string).
`dim_check`	Expected dimensionality or size.
`range_check`	Expected range.
`required`	1 or 0, to specify whether the default value is useful.
`default`	Default value to use if the parameter is not given.

`paramlist.outparams` defines the output parameters, and contains the following fields for each parameter:

`name`	Variable's name (string), the default output variable in the GUI.
`description`	Description to show the user (string).
`type`	Data type (string).
`suppress`	Suppress output? (0 or 1, optional, defaults to 0)

The parameter description depends on the parameter type. What each of `dim_check`, `range_check` and `default` mean depends on the type. Also, each parameter type produces

Table 7.1: Data type aliases used in the `range_check` parameter for images.

Name	maps to
`'any'`	`'complex'` + `bin`
`'complex'`	`'real'` + `scomplex, dcomplex`
`'noncomplex'`	`'real'` + `bin`
`'real'`	`'float'` + `'integer'`
`'int'` or `'integer'`	`'signed'` + `'unsigned'`
`'float'`	`sfloat, dfloat`
`'sint'` or `'signed'`	`sint8, sint16, sint32`
`'uint'` or `'unsigned'`	`uint8, uint16, uint32`

different controls in the GUI. Recognized types are listed below. Please examine any of the functions in the toolbox that put themselves on the menu to learn more about this structure.

`'image'`

An object of type `dip_image` (or `dip_image_array`). Numeric arrays are converted to a `dip_image`. The GUI presents an edit box where you can type any expression. Furthermore, a right-click in this edit box brings up a list with variables of class `dip_image` defined in the base workspace.

`dim_check` and `range_check` are used to specify the type of image expected. `dim_check` defines the allowed image dimensionalities through a two-element vector `[m,n]`, where `m` is the lowest dimensionality and `n` is the highest dimensionality allowed. The expressions `0` and `[]` map to `[0,Inf]`, meaning any dimensionality is OK. Any scalar `m` maps to `[m,m]`, meaning only images with `m` dimensions are allowed. For example, to limit your function to 2D and 3D images, use `[2,3]`.

`range_check` is a string or a cell array with strings that defines both the allowed data types and the image type (scalar, color, tensor, etc.) Allowed are any combination of `dip_image` data types (see Table 4.1) as well as the data type aliases defined in Table 7.1, and one of the following strings: `'scalar'` (requires `isscalar` to be `true`), `'tensor'` (`istensor` is `true`, which also allows a scalar image), `'vector'` (`isvector` is `true`, which does not allow a scalar image), `'color'` (`iscolor` is `true`) or `'array'` (any `dip_image` or `dip_image_array` object is OK). If none of this set is specified, `'tensor'` is assumed. If `range_check` is `[]`, `{'all','tensor'}` is used. There is no way to control the length of the vector or the dimensionality of the tensor, you will need to write code to check those sizes yourself.

`default` is a string to be evaluated in the base workspace (therefore, you can use any expression with names of variables in the base workspace). Typically you would use `'a'` or `'b'` as a default value, and set `required` to 1. This way, the GUI shows the name of a variable possibly containing an image, but at the command-line (assuming you use automatic parsing) this default value is never used. It is also possible to specify something like `'[1,1,1;1,1,1;1,1,1]'` as a default image (as does the function `convolve`).

`'measurement'`

An object of type dip_measurement. This input is treated the same as one of type 'image', except that dim_check and range_check are not used; set them to [] to avoid problems if these values become significant in the future.

'dataset'

An object of type dataset (from PRTOOLS). This input is treated the same as one of type 'image', except that dim_check and range_check are not used; set them to [] to avoid problems if these values become significant in the future.

'array'

Any MATLAB array. This is a complicated type because of the flexibility when specifying array size and data type.

dim_check defines the allowed array sizes in one of two ways:

- by referring to an image parameter using a positive integer scalar, the dimensionality of the image pointed to gives the length of the vector required as input here; or
- by directly giving an array size.

The first mode is useful when the array indicates e.g. a filter size (see gaussf) or a coordinate in the image (see findlocalmax). In both these cases one value per image dimension is required.

The second mode allows any array size, either fixed ([4,4] for a 3D transformation matrix) or flexible ([-1,3] for an RGB color map on any length). The -1 indicates that the length along that dimension is not tested for. The empty array [] indicates that an empty array is required. An empty array is not very useful, of course, except that we allow the combination of various size specifications using a cell array: {[],[1,3],[4,4]} indicates either an empty array, a 3-element vector or a 4-by-4 matrix are allowed. It is possible to combine references to image parameters and direct array sizes: {[],1} indicates either an empty array or a vector with as many elements as dimensions are in the first input image.

0 is a shortcut for [1,1], a scalar value. -1 is a shortcut for {[],[1,-1]}, a row vector of any length or an empty array.

When using automatic parameter parsing, if a scalar input is given it is extended to satisfy the required array size. Also, a vector is transposed to match the template, but two- or higher-dimensional arrays are not. If multiple array size options are given, the first one that matches is the one used.

range_check determines the valid range for the values in the array. It must be either an array with two values (minimum and maximum valid values), an empty array (meaning [-Inf Inf]), or one of a few strings that are defined for common ranges:

- Integer types: 'N+' = [1 Inf]. 'N-' = [-Inf -1]. 'N' = [0 Inf], 'Z' = [].
- Real types: 'R' = []. 'R+' = [0 Inf]. 'R-' = [-Inf 0].

Note that if you specify a range by two values, it is considered real. If you require some (finite) integer range, use the type 'option'.

If required is false, default is any array that satisfies the requirements of dim_check and range_check. For positive dim_check, provide a scalar as default value, since it is always

valid.

`'measureid'`

A measurement ID in a `dip_measurement` object.

`dim_check` is a positive integer that points to a parameter of type `'measurement'`. The GUI shows, in a drop-down list, all measurement IDs present in the referenced object. The automatic parameter parsing makes sure the measurement ID given by the user exists in the referenced object.

`required` should be 0, and `dim_check` and `default` are ignored. The default is always the first measurement in the `dip_measurement` object (passing the empty string yields the default as well).

`'option'`

A value (numerical or string) selected from a list. The GUI presents a drop-down list with options to choose from.

`range_check` is a `cell` array with possible options, for example:

- `{1,2,3,4}`
- `{'rectangular','elliptic','parabolic'}`

`required` should be 0. `default` is any one value from the list. `dim_check` is ignored.

`'optionarray'`

A `cell` array (with numbers or strings) selected from a list. The GUI presents an edit box with a button. Pressing the button brings up a dialog box that allows selecting one or more items from a list.

`range_check` is as in `'option'`. `required` should be 0. `default` is a `cell` array with values from the list, or a single value. `dim_check` is 0 if an empty cell array is allowed as input, 1 if at least one value is required.

`'cellarray'`

A `cell` array (with arbitrary cell content). `dim_check` and `range_check` are ignored. `default` must be a cellarray.

`'infile'`

The name of an existing file (for input). The GUI presents an edit box and a button that, when pressed, presents an "Open..." dialog box.

`range_check` is a string containing the mask for the file name, `dim_check` is ignored, and `default` is a string with the default file name.

`'outfile'`

The name of a file (for output). The GUI presents an edit box and a button that, when pressed, presents an "Save as..." dialog box. See the comments for `'infile'`.

`'indir'`

The GUI presents an edit box and a button that, when pressed, presents an "Select a directory ..." dialog box. `range_check` and `dim_check` are ignored, `default` gives the default directory.

`'handle'`

The handle of a figure window created by `dipshow`. It is possible to enter a handle or the name of a variable (the figure to which it is linked is used). The GUI shows a drop-down list with the titles of all figure windows that fit the description.

`range_check` is a `cell` array with strings that specify the type of figure window required. All figure windows that satisfy any of the strings are valid. Examples are:

- {`'1D','2D','3D'`} : either two- or three-dimensional displays.
- {`'Color','Grey','Binary'`} : either color, grey-value or binary displays.
- {`'1D_Color','2D_Grey'`} : either 1D color or 2D grey-value displays.

An empty array means that any window created by `dipshow` is acceptable. Note that these strings are not case-sensitive. It is, however, important that the order shown here is maintained. No window will satisfy the string `'Binary_2D'`, for example, but `'2D_Binary'` is valid.

`dim_check` and `default` are ignored. The default value is always `gcf` (the current figure).

`'string'`

Any string. `dim_check` and `range_check` are ignored. `default` must be a string.

`'boolean'`

The value 1 or 0. Also accepted are the strings `'yes'`, `'no'`, `'true'` and `'false'`, as well as only the first character of each. The GUI presents a drop-down box with the words "yes" and "no". The automatic parameter parsing, however, always returns either 1 or 0. `dim_check` and `range_check` are ignored. `default` should be any of the accepted values.

7.13 Automatic Parameter Parsing

To use automatic parameter parsing (through the `getparams` function), you no longer (since version 1.4.1) need to copy files from the `dipimage/private/` directory into your own `private/` directory. The function `getparams` is directly available.

The code shown in Figure 7.3 needs to be inserted into your function (after the portion used for the GUI functionality). As you can see, the same data structure `paramlist` is used for automatic parameter parsing and for the GUI.

It is not necessary to use the function `getparams`. If you don't, you will have a more flexible parameter parsing, but if you do, you will need to write less code: parameters are guaranteed to be of the chosen types and in the chosen intervals.

```
% The next section handles all parameter parsing
try
   [var1,var2,var3] = getparams(paramlist,varargin{:});
catch
   if ~isempty(paramerror)
      error(paramerror)
   else
      error(firsterr)
   end
end
% Below, add your own code
image_out = process_image(var1,var2,var3);
```

Figure 7.3: Skeleton for a function that uses automatic parameter parsing.

Chapter 8

Customizing the DIP*image* Environment

8.1 Figure Windows

The single most important thing that can be customized in the DIP*image* environment is the way that images are displayed to figure windows. It is possible to link a variable name with a figure handle, such that that variable is always displayed in that same window. If a variable is not linked to any window, a new one will be opened to display it. The command `dipfig` is used to create these links (see Section 7.7).

8.2 Graphical user Interface

The DIP*image* toolbox contains a GUI with a menu system for easy calling of toolbox functions. It is not necessary to use this GUI, but it is the easy way of finding the functions defined in the toolbox (see Section 7.1).

All functions that appear on the menus are in the toolbox directory or on the DIP*image* path. If you want to add any functions to this menu system, read Section 7.12. If you want your function to appear in a specific place in the menu system, you will have to create a function called `localdipmenus`. It gives you the opportunity to edit the cell array `menulist` created by `dipmenus`, which specifies in which menu each function should be placed. It also allows you to provide a list of functions not to be put on the menus at all.

The cell array `menulist` has two columns. The left column gives the names of the menus, the right column contains cell arrays with the function names and menu names that are to be put under each menu. Any function not mentioned in this array will be put at the bottom of the menu specified by the function itself, in alphabetical order. See the code for `dipmenus` to see how it is defined.

The list of functions to be excluded overrides the `menulist`. Any function in this list will not be queried when generating the menu system.

Figure 8.1 provides an example for a `localdipmenus` function. It adds a menu to the `menulist`, and puts all AVI-related functions on the `exclude` list. Note the string `'-'` that inserts a separator in the menu.

An alternative is to edit the `dipmenus` function. We do not recommend this because you will be required to make the same changes each time you install a new version of DIP*image*.

The DIP*image* GUI will call the `dipinit` command when starting. It initializes the working environment. See Section 8.3.

```
function [menulist,excludelist] = localdipmenus(menulist)
I = size(menulist,1)+1;
menulist{I,1} = 'My Functions';
menulist{I,2} = {'gaussf','unif','kuwahara','-','closing','opening'};
excludelist = {'readavi','writeavi','writedisplayavi'};
```

Figure 8.1: Sample `localdipmenus` function.

Another thing that can be customized in the GUI is whether the command it executes should be printed to MATLAB's command window. This is useful for copying and pasting the command being executed to some script or function. It is on by default, and can be switched off by typing

```
dipsetpref('PutInCommandWindow','off')
```

8.3 Initialization File

The DIP*image* GUI will call the `dipinit` command when starting. It initializes the working environment, setting up figure windows and the like. You can also call it yourself, to return the windows to their starting positions. You can edit this file to suit your need (or you can create a local copy, making sure that it sits on the MATLAB path before the original one; this is recommended in multi-user systems). Since it is a script, not a function, it can initialize some variables if you like. It can also be used to position the DIP*image* GUI to the place of your liking:

```
set(0,'ShowHiddenHandles','on')
h = findobj('tag','DIPimage_Main_Window');
set(h,'Position',[500,600,500,100])
set(0,'ShowHiddenHandles','off')
```

8.4 Other Settings

Other settings are available through the `dipsetpref` command (see Section 7.8). They are listed below:

`BinaryDisplayColor`

Value: 3x1 array of floats between 0 and 1

Default: `[1 0 0]`

This specifies the color used to display the object pixels in a binary image. Be default they are red, out of historical reasons. Some people prefer a different color, such as `[1 1 1]` or `[0 1 0]`.

`BoundaryCondition`

Value: string

Default: `'symmetric'`

Setting this value causes `dip_setboundary` to be called. This causes the algorithm that extends the image beyond its boundary to change, for all filter operations.

`BringToFrontOnDisplay`

Value: `'on'` or `'off'`

Default: `'on'`

This setting controls whether `dipshow` brings a window to the front when displaying a new image, or updating an old one.

`CommandFilePath`

Value: string

Default: `''`

This setting stores the path used by the DIP*image* GUI to find the functions that must be added to the menu system. The DIP*image* toolbox directory does not need to be in this path, since it is always used. On UNIX and Linux systems, directories are separated by a colon (`:`), on Windows systems by a semicolon (`;`).

`ComplexMappingDisplay`

Value: string

Default: `'x+iy'`

This only affects display of complex images. When using the *Pixel testing* mode in the image display window, the pixel value can be displayed as real and imaginary components (`'x+iy'`), or as magnitude and phase components(`'r/phi'`).

`ComputationLimit`

Value: integer

Default: $64 * 1024^2$

This only affects operations done on `dip_image` objects in MATLAB (not operations that involve DIP*lib* itself). MATLAB can only compute (properly) using floating-point values, so images of integer types are converted to either `single` or `double` to do the computation. To avoid excessive memory usage, the images are chopped in blocks to do this conversion. `ComputationLimit` sets the size of these blocks, in bytes. Versions of MATLAB prior to 7.0 can only compute using double-precision floats, so images of type `single` are also processed this way.

`ConflictingPixelSize`

Value: `'ignore'`, `'first'`, `'second'`, `'pixel'`, `'warning'` or `'error'`

Default: `'first'`

If the pixel sizes of two images in an arithmetic operation are different, this option specifies

what should happen. The `'pixel'` and `'warning'` option cause the output image to have the default pixel size along all dimensions. The `'error'` option disallows the operation by generating an error message. The `'ignore'` and `'first'` options keep the pixel size of the first image. The `'second'` option keeps the pixel size of the second image.

`CurrentImageFileDir`

Value: string

Default: `''`

This setting stores the directory last visited by the file selection dialog boxes of `readim`, `readcolorim`, `readroiim` and `writeim`. It is used by these functions to open the file selection dialog box in the directory you last used.

`CurrentImageSaveDir`

Value: string

Default: `''`

This setting stores the directory last visited by the file selection dialog box of the "Save display..." option of the "File" menu of the figure windows. It is used to open the file selection dialog box in the directory you last used. An empty string means that the current directory is to be used.

`DebugMode`

Value: `'on'` or `'off'`

Default: `'off'`

When this option is turned on, error messages are more verbose, and errors in the DIP*image* toolbox are easier to track. It is used for developing GUI functions.

`DefaultActionState`

Value: string

Default: `'diptest'`

This is the action mode that will be enabled by `dipshow` when displaying an image to a new window, or to a window with a mode not compatible with the image being displayed. Possible values are `'none'`, `'diptest'`, `'diporien'`, `'dipzoom'` and `'dipstep'`. See Section 6.2.

`DefaultColorMap`

Value: string

Default: `'grey'`

This is the colormap that will be used by `dipshow` when displaying an image to a new window. Possible values are `'grey'`, `'periodic'`, `'saturation'`, `'zerobased'` and `'labels'`. See Sections 7.3 and 6.1.

`DefaultComplexMapping`

Value: string

Default: `'abs'`

This is the complex mapping mode that will be enabled by `dipshow` when displaying an image to a new window, or to a window with a mode not compatible with the image being displayed. Possible values are `'abs'`, `'phase'`, `'real'` and `'imag'`. See Sections 7.3 and 6.1.

`DefaultFigureHeight`

Value: integer

Default: `256`

This value determines the height of a window created by `dipshow` or `dipfig`, unless a size is explicitly given.

`DefaultFigureWidth`

Value: integer

Default: `256`

This value determines the width of a window created by `dipshow` or `dipfig`, unless a size is explicitly given.

`DefaultGlobalStretch`

Value: `'on'` or `'off'`

Default: `'off'`

Set this option if you want global stretching for 3D/4D images on by default. See Sections 7.3 and 6.1.

`DefaultMappingMode`

Value: string

Default: `'normal'`

This is the mapping mode that will be enabled by `dipshow` when displaying an image to a new window, or to a window with a mode not compatible with the image being displayed. Possible values are `'lin'`, `'percentile'`, `'log'`, `'base'`, `'angle'` and `'orientation'`. See Sections 7.3 and 6.1.

`DefaultSlicing`

Value: string

Default: `'xy'`

Sets the direction in which 3D/4D volumes are sliced by default. Possible values are `'xy'`, `'xz'` and `'yz'`. See Sections 7.3 and 6.1.

`DerivativeFlavour`

Value: string

Default: `'spatial'`

Sets the way Gaussian derivatives are computed: either by spatial convolution with a Gaussian derivative or via the Fourier domain. The second is slower and uses more memory, but will be more accurate for small sigmas. Possible values are `'spatial'` and `'fourier'`.

`DisplayToFigure`

Value: `'on'` or `'off'`

Default: `'on'`

When this setting is `'on'`, the `display` method of the `dip_image` object sends the image data to a figure window. When it is `'off'`, `disp` is called instead. The display method is called when a MATLAB command does not end with a semicolon. See Section 4.2 for more information on this behavior.

`EnableKeyboard`

Value: `'on'` or `'off'`

Default: `'on'`

If you set this value to `'off'`, the keyboard will be disabled when displaying an image. This is useful for Windows machines, on which the figure window will get keyboard focus when displaying an image. This can be annoying when you want to continue typing. Enable the keyboard callback for a figure window using the appropriate menu item under "Actions".

`FileWriteWarning`

Value: `'on'` or `'off'`

Default: `'off'`

If you set this to `'on'` everything you write a non-standard TIFF image in terms of byte depth or compression a warning will be displayed on the screen. This is useful as many image viewer cannot read anything but `uint8` uncompressed images (e.g. the standard Windows image TIFF viewer).

`FFTtype`

Value: `'diplib'` or `'fftw'`

Default: `'diplib'`

Selects which FFT routine is used for computation in the functions `'ft'` and `'ift'`. The `'diplib'` option uses the routine in DIP*lib*, the `'fftw'` option uses the `'fftn'` routine in MATLAB, which uses the FFTW library. The `'fftw'` option is usually much faster.

`Gamma`

Value: 3x1 array of floats

Default: `[1 1 1]`

These parameters control the display of all colour images shown by `dipshow`. If the values are different from unity a gamma correction is applied before displaying any image. The different

values control the behaviour for the Red, Green and Blue channel respectively.

`GammaGrey`

Value: float

Default: `1`

Similar to `'Gamma'`, but only for grey-value images. This parameter controls the display of all grey-value images shown by `dipshow`. If the value is different from unity a gamma correction is applied before displaying any image.

`ImageFilePath`

Value: string

Default: `''`

This setting stores the path used to find image files. The functions `readim`, `readcolorim` and `readroiim` look for a file first in the current directory, and then in each of the directories given by this option, unless the filename already contains a path. On UNIX and Linux systems, directories are separated by a colon (`:`), on Windows systems by a semicolon (`;`).

`ImageSizeLimit`

Value: integer

Default: `4096`

This is the maximum size of an image automatically displayed through `display`. If any of the sizes of an image is larger, you will need to display it manually using `dipshow`. The reason behind this behavior is that such an image is most likely to be created accidentally, and not meant for display anyway. For example, `a(a>10)` returns a 1D image with all pixel values of `a` larger than 10; this is very useful, but not interesting to look at. For a large `a` (such as a 3D image), the display of the resulting 1D image might require a lot of memory.

`InconsistentPixelSize`

Value: `'ignore'`, `'pixel'`, `'warning'` or `'error'`

Default: `'pixel'`

If the pixel sizes along the various dimensions of an image are different, this option specifies what should happen when these dimensions are mixed. The `'pixel'` and `'warning'` option cause the output image to have the default pixel size along all dimensions. The `'ignore'` option keeps the pixel size information as is. The `'error'` option disallows the operation by generating an error message.

`KeepDataType`

Value: `'on'` or `'off'`

Default: `'off'`

Setting this option to `'on'` causes arithmetic operations to produce images of the same data type as the input images. In the case of an operation between an image and a MATLAB array,

the data type of the image is used. In case of an operation between two images with different type, the larger of the two types is chosen for the result.

`MorphologicalFlavour`

Value: integer

Default: `0`

This setting stores the state usually set though `dip_morph_flavour`. The value `0` causes the morphological operations to follow the definition of Serra and Soille, `2` is for the definition followed by Heijmans and Haralick. Any other value is equivalent to `0`.

`NumberOfThreads`

Value: integer

Default: usually the number of cores in your system, or the value given by the `OMP_NUM_THREAD` environment variable.

The number of threads used for computation by DIP*lib*. This does not affect the computations performed by MATLAB itself.

`PutInCommandWindow`

Value: `'on'` or `'off'`

Default: `'on'`

This option causes commands that are executed from the DIP*image* GUI to be printed to the command window. This makes it possible to copy and paste commands being executed to a MATLAB script.

`RespectVisibility`

Value: `'on'` or `'off'`

Default: `'off'`

By default, `dipshow` hides a window while it prepares for displaying a new image, then makes it visible again. This speeds up the process, and removes flickering. Setting `'RespectVisibility'` to `'on'` the window remains visible if it was visible (some flickering might occur), and hidden if it was hidden.

`TrueSize`

Value: `'on'` or `'off'`

Default: `'on'`

This setting controls whether `diptruesize` is called after an image is displayed to a figure window (see Section 7.4).

`Truncation`

Value: integer

Default: `3`

Setting this value causes `dip_settruncation` to be called. This changes all finite impulse response Gaussian filters (this number represents the extent of the filtering kernel, in terms of the given parameter sigma). Note that the Gaussian filters are also available as IIR (infinite impulse response) filters and as Fourier Domain filters; these versions are not affected by the truncation parameter.

`UserManualLocation`

Value: string

Default: The URL needed to fetch the user manual online.

This setting stores the location of the DIP*image* User Manual (a PDF file). By default it points to an address online, but you can change it to point to a local copy of the PDF file. A link on the Help menu of the DIP*image* GUI and on the MATLAB Start Button are affected by this setting.

Chapter 9

Low-level DIP*lib* Interface

The DIP*image* toolbox is build around DIP*lib*, which is a library of image-processing functions written in C. Most of these functions can be directly called from within MATLAB through a low-level interface. This interface is not as easy to use as the toolbox functions, but it is more complete.

9.1 The Setup

For each function available in the low-level interface, there is a MEX-file (which is just a shared object MATLAB links to), and an M-file, which just contains some help on calling the function. However, this help is very meager, since it only lists the name and type of each parameter. Only when the function does more than just call the equivalent DIP*lib* function, are there any comments on how the function works. You will need to check the online DIP*lib* documentation to see what each of the parameters does. The online function reference can be found at:
ftp://ftp.tudelft.nl/pub/DIPimage/latest/docs/reference/index.html.

9.2 Calling DIP*lib* Functions

You will notice that the parameters required by the interface are exactly those required by the C functions, but with some exceptions:

- The output parameters are naturally placed at the left-hand side of the function call. In the corresponding C functions, they are always on the right-hand side. The interface generates an error when the C function returns an error code.
- The parameters corresponding to the random number generator are stored internally by the interface and thus are not needed at the command line.
- The parameters corresponding to the extension of the image beyond the boundaries and the truncation of the Gaussian kernel are also not present in the interface. The default values for these parameters are used. These defaults can be set and read with the functions `dip_setboundary`, `dip_getboundary`, `dip_settruncation` and `dip_gettruncation`.
- None of the DIP*lib* library variables is passed back to MATLAB. This means that any parameter of the type `dip_Resources` and the like are not present in the MATLAB interface.
- Enumerated values in the C functions have been implemented as strings in the MATLAB interface. The function's help will list these strings, which are easy to map to the names

of the enumerated values (use `help parameters` if in doubt). If a function requires an array of these values, the strings should be put in a `cell` array.

By the way, all DIP*lib* interface functions have all characters in lower-case, and start with the characters "`dip_`", or "`dipio_`" for the functions in the dipIO extension library.

Whenever an image is required as input, it is legal to pass either a numeric MATLAB array or a `dip_image` object. Parameters of the type `dip_FloatArray` and the like are usually expected to be of the same length as the dimensionality of the input image. The low-level interface is so low-level that it does not even check these simple things, and DIP*lib* will generate an error if the array is not of the correct size. Many parameters are allowed to be null pointers in the C library. Sometimes it is possible to pass an empty array as such a value (for example, pass an empty array as a mask image if no masking is required).

9.3 Example Function Call

As an example, let us call the DIP*lib* function`dip_Gauss` from within MATLAB. The declaration of the C function is:

```
dip_Error dip_Gauss (
  dip_Image in,
  dip_Image out,
  dip_BoundaryArray boundary,
  dip_BooleanArray process,
  dip_FloatArray sigmas,
  dip_IntegerArray parOrder,
  dip_float truncation
);
```

As explained earlier, the parameters `boundary` and `truncation` should not be used from within MATLAB. The globally defined default values will be used. The parameter `out` should be on the left of the function call. What remains is this:

```
out = dip_gauss(in,process,sigmas,parOrder);
```

which we can verify by typing

```
help dip_gauss
```

This also gives us the expected data types for each parameter. The data types correspond

with those expected by the C function:

```
dip_gauss   Gaussian Filter.

out = dip_gauss(in, process, sigmas, parOrder)

in
   Image.
process
   Boolean array.
sigmas
   Real array.
parOrder
   Integer array.
```

Parameter `in` can be a `dip_image` or any numeric array. The length of the other arrays should match the dimensionality of the image. We will use a two-dimensional image `a`, which we want to smooth by convolution with a Gaussian with sigma 5 in the y-direction and 2 in the x-direction. We would write

```
b = dip_gauss(a,[1,1],[2,5],[0,0]);
```

As can be read in the online help for DIP*lib*, `parOrder` indicates the order of the derivative. The `process` array, which is present in many DIP*lib* functions, can be used to apply the filter only in some dimensions.

The `dipimage/demos/` directory contains some example M-files. Examine `demogdt.m` for an example on using the low-level interface.

Chapter 10

DIP*image* and the MATLAB Compiler

10.1 The MATLAB Compiler

Since MATLAB version 7.0 (Release 14), the MATLAB Compiler no longer generates C or C++ code from M-files. Instead, it packages all M-files and MEX-files into a *Component Technology File* (CTF) archive, generates a small stub executable, and requires the end user to install the MATLAB Component Runtime (MCR). This MCR is the MATLAB interpreter (but without licensing restrictions). The upside of this is that there are no longer limitations as to what M-files can be compiled, meaning it is now possible to create standalone applications that use DIP*image*. The downside is that, since code is not really compiled, there is no performance benefit to compiling.

The MATLAB compiler can generate shared objects (dynamically linked libraries) as well as executables. This means that it is still possible to compile M-file code so that it can be called from your own C or C++ code, even though this compiled M-file code can no longer be statically linked into your executable.

M-files in the CTF archive are encrypted so that it is not possible to obtain source code from the compiled application. MEX-files are also protected in some way so they cannot be run outside of the deployed application. Therefore, even though the code is not truly compiled, your code is reasonably well protected against reverse-engineering.

The explanations below are for Linux/UNIX systems. If you use Windows, similar issues will have to be taken into account. The MATLAB Compiler User's Guide (available online at http://www.mathworks.com/access/helpdesk/help/pdf_doc/compiler/compiler.pdf) contains all the information needed to compile an M-file that uses DIP*image*.

10.2 Compiling an M-file that uses DIP*image*

Please first read the MATLAB Compiler User's Guide, and make sure you are able to generate the `magicsquare.m` stand-alone example application using the `mcc` command (not through the `deploytool` GUI, since the explanations below assume you are familiar with `mcc`).

There are a few things that have to be taken into account when your M-file uses DIP*image*. First, like with other toolboxes, the DIP*image* directory must not be added to the MATLAB path through the `startup.m` file (as suggested in the DIP*image* installation instructions), but through the `mcc` command line. Second, instead of calling `dip_initialise`, call `dip_initialise_libs`.

`dip_initialise` searches for the correct version of the DIP*image* toolbox to use, depend-

ing on the MATLAB version you are running. It then adds the necessary paths and calls `dip_initialise_libs`. Since this process doesn't work with the MATLAB Compiler, you will need to do these two steps separately.

Hence, you need to create a special version of your `startup.m` file in the directory where your application M-file lives. Remove all the `addpath` instructions, and change the line `dip_initialise` into `dip_initialise_libs` (if you do not want the DIP*lib* version information to be displayed on startup, you can use the `'silent'` argument to `dip_initialise_libs`). Alternatively, you can call the `dip_initialise_libs` function in your application M-file. In this case, make and empty `startup.m` file to avoid your default one to be used.

To find out which directories you need to add to the Compiler search path, type `path` on the MATLAB command line. It should return a long list of directories, three of which look like this:

```
/something/dip/common/mlv7_4/diplib
/something/dip/common/mlv7_4/dipimage_mex
/something/dip/common/dipimage
```

These three paths can be added the the `mcc` command line using the '`-I`' argument:

```
mcc -m myapplication.m ...
    -I /something/dip/common/dipimage ...
    -I /something/dip/common/mlv7_4/dipimage_mex ...
    -I /something/dip/common/mlv7_4/diplib
```

Under some circumstances, `mcc` might give a warning telling you that the `dip_initialise_libs` command is unknown. However, when running the resulting executable, DIP*lib* gets initialized just fine. This must be due to the order in which paths get added and commands are executed.

When running the stand-alone application you just created, the three DIP*lib* shared libraries must be on the `LD_LIBRARY_PATH` environment variable, as discussed in Section 2.2. It is possible to edit the shell script that is created by `mcc` (`run_myapplication.sh`) to properly set the `LD_LIBRARY_PATH` environment variable.

10.3 Deploying your compiled program

First of all, note that you need a special license of DIP*image* and DIP*lib* to be able to distribute a program that uses this toolbox and associated libraries. Please read our web page (http://www.diplib.org/) for information on how to obtain such a license.

The CTF file created by `mcc` needs either the exact same version of MATLAB, or the MCR created with that version, to run. It will also need the three DIP*lib* shared libraries `libdip.so`, `libdipio.so` and `libdml_mlvX_X.so` (the name of this last SO file should match the directory name given as path to the MATLAB Compiler). The end-user needs to install these three libraries and adjust the `LD_LIBRARY_PATH` environment variable prior to starting the executable.

There is a very simple way of including the DIP*lib* libraries in the CTF file:

```
mcc -m myapplication.m ...
    -I /something/dip/common/dipimage ...
    -I /something/dip/common/mlv7_4/dipimage_mex ...
    -I /something/dip/common/mlv7_4/diplib ...
    -a /something/dip/Linux/libdip.so ...
    -a /something/dip/Linux/libdipio.so ...
    -a /something/dip/Linux/libdml_mlv7_4.so
```

The CTF archive will be called `myapplication.ctf`, and, once extracted, the DIP*lib* libraries will be in the directory `myapplication_mcr/something/dip/Linux/` (assuming 32-bit Linux OS).

Thus, assuming your user puts the files `myapplication.ctf` and `myapplication` into the directory `/home/user/myapp/`, and installed the MCR into `/usr/local/mcr/v76/`, your user will have to do the following to start the application:

```
MCRROOT=/usr/local/mcr/v76
LD_LIBRARY_PATH=/home/user/myapp/myapplication_mcr/something/dip/Linux/
LD_LIBRARY_PATH=${LD_LIBRARY_PATH}:${MCRROOT}/runtime/glnx86
LD_LIBRARY_PATH=${LD_LIBRARY_PATH}:${MCRROOT}/bin/glnx86
LD_LIBRARY_PATH=${LD_LIBRARY_PATH}:${MCRROOT}/sys/os/glnx86
MCRJREVER=`cat ${MCRROOT}/sys/java/jre/glnx86/jre.cfg`
MCRJRE=${MCRROOT}/sys/java/jre/glnx86/jre${MCRJREVER}/lib/i386
LD_LIBRARY_PATH=${LD_LIBRARY_PATH}:${MCRJRE}/native_threads
LD_LIBRARY_PATH=${LD_LIBRARY_PATH}:${MCRJRE}/server
LD_LIBRARY_PATH=${LD_LIBRARY_PATH}:${MCRJRE}/client
LD_LIBRARY_PATH=${LD_LIBRARY_PATH}:${MCRJRE}
XAPPLRESDIR=${MCRROOT}/X11/app-defaults
export LD_LIBRARY_PATH
export XAPPLRESDIR
/home/user/myapp/myapplication <arguments>
```

You would do good creating a little shell script that collects these commands, and provide it with your executable (instead of `run_myapplication.sh`).

Note that, in the shell commands above, the ones that contain `MCRJRE` are needed only if Java is enabled. You can add `-R "-nojvm"` to the `mcc` command to disable Java if your application does not use it. In the same way, add `-R "-nodisplay"` if your application does not use the graphic display.
