# Supplementary figures and images for: Precision Automation of Cell Type Classification and Sub-Cellular Fluorescence Quantification from Laser Scanning Confocal Images
Source: Front Plant Sci. 2016 Feb 9;7:119. doi: 10.3389/fpls.2016.00119 (PMC4746258; doi:10.3389/fpls.2016.00119)

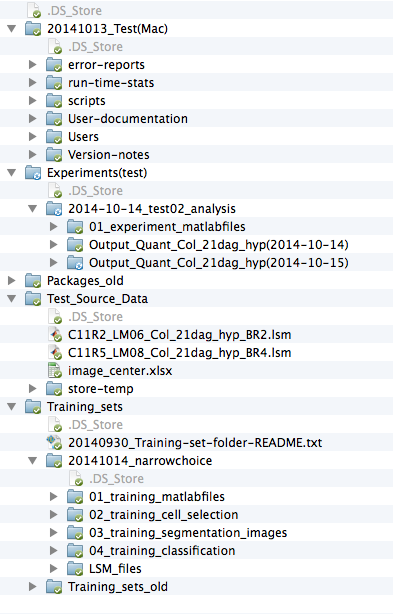

Supplement: Supplementary file 3 [file DataSheet1.ZIP › 20151214_MatlabFiles/UserDocs/Data_handling_Directory_images/MasterDirectory_all.png]

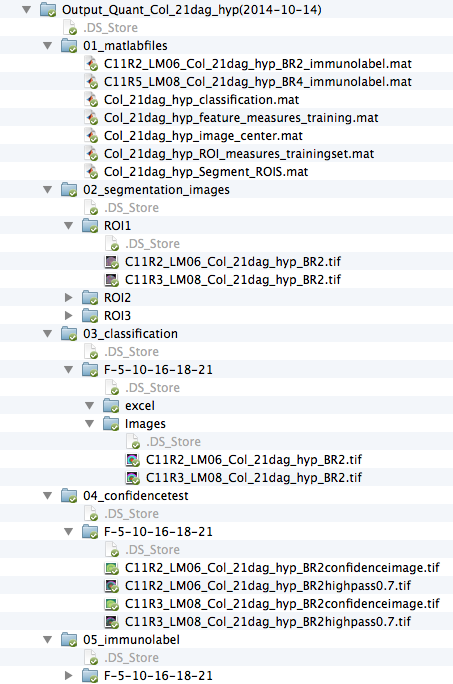

Supplement: Supplementary file 3 [file DataSheet1.ZIP › 20151214_MatlabFiles/UserDocs/Data_handling_Directory_images/Output_directory.png]

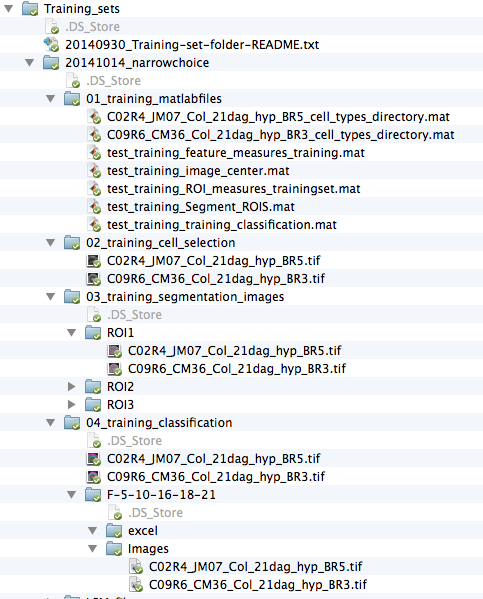

Supplement: Supplementary file 3 [file DataSheet1.ZIP › 20151214_MatlabFiles/UserDocs/Data_handling_Directory_images/training-set-directory.png]

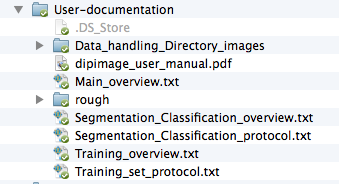

Supplement: Supplementary file 3 [file DataSheet1.ZIP › 20151214_MatlabFiles/UserDocs/Data_handling_Directory_images/User-documentation.png]

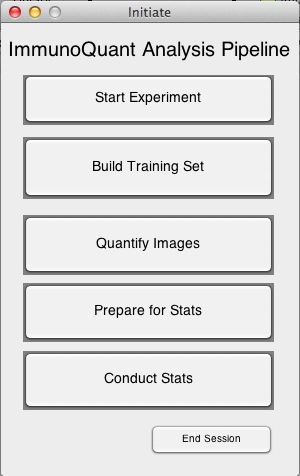

Supplement: Supplementary file 3 [file DataSheet1.ZIP › 20151214_MatlabFiles/UserDocs/Images_pipeline/InitiateGUI20141017.jpg]
